# Synthesis of Tris-Heterocycles via a Cascade IMCR/Aza Diels-Alder + CuAAC Strategy

**DOI:** 10.3389/fchem.2019.00546

**Published:** 2019-08-06

**Authors:** Manuel A. Rentería-Gómez, Alejandro Islas-Jácome, Shrikant G. Pharande, David A. Vosburg, Rocío Gámez-Montaño

**Affiliations:** ^1^Departamento de Química, Universidad de Guanajuato, Guanajuato, Mexico; ^2^Departamento de Química, Universidad Autónoma Metropolitana-Iztapalapa, Mexico City, Mexico; ^3^Department of Chemistry, Harvey Mudd College, Claremont, CA, United States

**Keywords:** nitrogen tris-heterocycles, cascade IMCR process, CuAAC, Ugi-3CR, aza Diels-Alder

## Abstract

6-Triazolylmethyl-pyrrolo[3,4-*b*]pyridin-5-one tris-heterocycles were synthesized in 43–57% overall yields. The two-stage synthesis involved a cascade process (Ugi-3CR/aza Diels-Alder/*N*-acylation/aromatization) followed by a copper-assisted alkyne-azide [3+2] cycloaddition (CuAAC). This efficient and convergent strategy proceeded via complex terminal alkynes functionalized with a fused bis-heterocycle at the α-position. The final products are ideal candidates for SAR studies as they possess two privileged scaffolds in medicinal chemistry: 4-substituted or 1,4-substituted 1*H*-1,2,3-triazoles and pyrrolo[3,4-*b*]pyridin-5-ones.

## Introduction

Polyheterocycles are organic molecules containing three or more heterocyclic moieties, which may be joined by one or more different kinds of connectivity (Ibarra et al., 2018). Nitrogen-containing polyheterocycles are of particular interest in the synthesis of bioactive molecules (Dener et al., 2006; Dolle et al., 2008; Atobe et al., 2013). Tris-heterocyclic molecules have been reported in optics and in coordination chemistry (Stibrany et al., 2003; Burling et al., 2007; Tahara et al., 2009).

**Figure 1:**
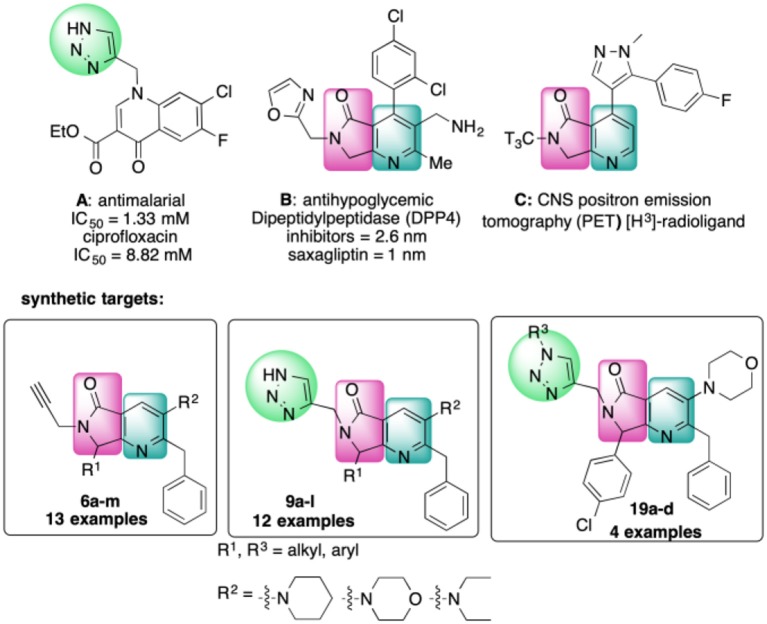
Bioactive bis- and tris-heterocycles and our synthetic targets.

The 4-substituted 1*H*-1,2,3-triazole is a heterocycle of high interest in medicinal chemistry, and it is well-documented that incorporation of this moiety into several bioactive compounds has resulted in advantages such as reduced toxicity or increased antibacterial or antimalarial activity (Shchepin et al., 2008; Zhou et al., 2008) [e.g., Figure 1, compound **A** (Dixit et al., 2012)]. Triazoles may also increase the stability and polarity of compounds by coordination of the N1 and N2 triazole nitrogens to active-site metal atoms in metalloproteases. The N3 nitrogen appears not to be directly involved in binding metals but can form hydrogen bonds with amino acid residues (Kallander et al., 2005; Huang et al., 2011; Röhri et al., 2012; Borkin et al., 2016). Additionally, 4-substituted 1*H*-1,2,3-triazoles are precursors of bioactive disubstituted 1,2,3-triazoles (Duan et al., 2009; Oh et al., 2010; Yan et al., 2010; Hsu et al., 2013; Bakulev and Beryozkina, 2016) and of pyridyl-1*H*-1,2,3-triazolate complexes that have applications in optics and coordination chemistry (Sinn et al., 2014; Prabhath et al., 2015). On the other hand, 1,4-disubstituted 1*H*-1,2,3-triazoles display structural and electronic similarities with the *trans*-amide bond. Their overall dipolar moment and hydrogen-bonding properties are greater than those of an amide bond, making these heterocycles effective peptidomimetics (Tron et al., 2008). These triazoles also function as flat bivalent elements, imitating the rigid conformational constraints of double bonds in alkyl chains. 1,4-Disubstituted 1*H*-1,2,3-triazoles are also capable substitutes for other five-membered nitrogen-containing heterocycles such as imidazoles, pyrazoles, 1,2,4-triazoles, oxazoles, isoxazoles, and oxazolidinones. Finally, these rings can act as more stable isosteres of phosphate linkers (Bonandi et al., 2017).

Common synthetic approaches to 4-substituted 1*H*-1,2,3-triazoles involve a [3+2] cycloaddition between sodium azide (NaN_3_) or hydrazoic acid (HN_3_) and terminal alkynes activated with electron-withdrawing groups (EWGs) (Hartzel and Benson, 1954; Balle et al., 2003; Blass et al., 2006; Koszytkowska-Stawińska et al., 2012). A variation of this classical methodology using trimethylsilyl azide (TMSN_3_) provides a much safer procedure. The use of a copper(I) catalyst allows the use of non-activated terminal alkynes in shorter reaction times and under milder conditions (Jin et al., 2004). For preparing 1,4-disubstituted 1-*H*-1,2,3-triazoles, the classic synthetic approach is the regiospecific copper-assisted [3+2] cycloaddition between terminal alkynes with organic azides (Rostovtsev et al., 2002; Tornøe et al., 2002). The most common technique is *in situ* reduction of copper(II) salts, such as CuSO_4_•5H_2_O or Cu(OAc)_2_, forming copper(I) salts using sodium ascorbate as the reducing agent. A second option is to use a copper(I) salt such as CuCl, CuBr, CuI, [Cu(CH_3_CN)_4_]OTf, or Cu(CH_3_CN)_4_PF_6_ in a deoxygenated environment and in organic solvent, typically with an amine such as TEA, DIEA, DIPEA, or PMDETA (Bock et al., 2006; Hein and Fokin, 2010; Lauria et al., 2014).

On the other hand, the fused heterocycle pyrrolo[3,4-*b*]pyridin-5-one is an aza-analog of isoindolin-1-one natural products and is present in several bioactive molecules; for example: hypoglycemic [Figure 1, compound **B** (Devasthale et al., 2013)], analgesic, anticancer, and therapeutic agents for CNS-related diseases like Alzheimer's, epilepsy, and schizophrenia (Unverferth et al., 2002; Chang et al., 2008; Pajouhesh et al., 2012; Lindsley et al., 2013). The synthesis of analogs with a brain-selective radioligand has also been reported [Figure 1, compound **C** (Wager et al., 2017)].

There are no previous reports of molecules containing 4-substituted or 1,4-disubstituted 1*H*-1,2,3-triazoles and pyrrolo[3,4-*b*]pyridin-5-ones, though there are a few examples of each of these ring systems connected to other heterocycles using multistep approaches [Figure 1, compounds **A**-**C**) (Dixit et al., 2012; Devasthale et al., 2013; Mallemula et al., 2015; Maračić et al., 2015; Wager et al., 2017)].

**Scheme 1:**
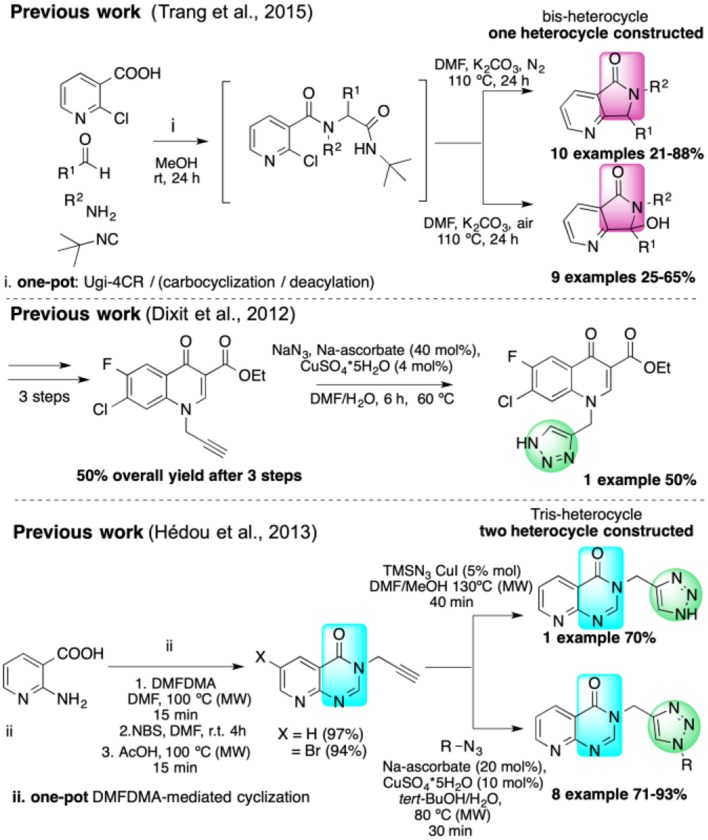
Previous works.

Isocyanide-based multicomponent reactions (IMCRs) are the most efficient strategies to synthesize pyrrolo[3,4-*b*]pyridin-5-ones, and Zhu first reported a one-pot synthesis in 2001 (Sun et al., 2001). We synthesized various annulated polyheterocycles containing this fused bis-heterocycle via IMCR strategies (Islas-Jácome et al., 2011, 2012). However, there are no published reports of pyrrolo[3,4-*b*]pyridin-5-ones linked to other heterocycles in a non-annulated fashion using IMCRs. Recently, Van der Eycken reported a one-pot synthesis of disubstituted pyrrolo[3,4-*b*]pyridin-5-ones by an Ugi four-component reaction (Ugi-4CR)/carbocyclization/deacylation sequence (Scheme 1; Trang et al., 2015). While that work can generate oxidized 7-hydroxy derivatives, we sought a route that would feature improved atom economy, shorter reaction times, and milder conditions in addition to greater structural complexity with more functionalized products (Scheme 1).

Besson reported the two-stage synthesis of tris-heterocycles with pyrido[2,3-*d*]pyrimidin-4(3*H*)-one linked to 1,2,3-triazoles via a one-pot DMFDMA-mediated cyclization followed by a CuAAC (Scheme 1; Hédou et al., 2013). However, only two heterocycles are constructed in this process and the scope of the alkynes is limited.

**Scheme 2:**
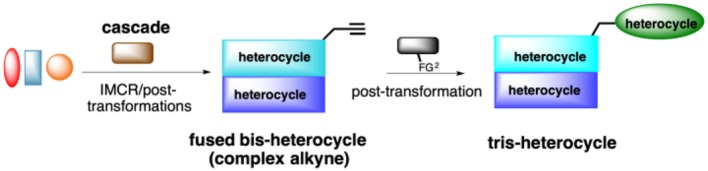
Cascade IMCR /[3+2] cycloaddition strategy.

Our ongoing research program focuses on the design of rapid, convergent and efficient IMCR/post-transformation strategies toward novel molecules containing privileged heterocycles: azepino[4,5-*b*]indol-4-ones (Rentería-Gómez et al., 2016b), 2-tetrazolylmethyl-isoindolin-1-ones (Rentería-Gómez et al., 2016a), tetrazolo[1,5-*a*]quinolines (Unnamatla et al., 2016), 3-tetrazolylmethyl-azepino[4,5-*b*]indol-4-ones (Gordillo-Cruz et al., 2013), 2,3,4,9-tetrahydro-1*H*-β-carbolines (Cárdenas-Galindo et al., 2014), 4-(pyridine-3-yl)pyrimidines (Cortes-García et al., 2016), and spiro[pyrrolidine-3,3′-oxindoles] (Alvárez-Rodríguez et al., 2018). Herein we describe the first cascade IMCR process/post-transformation strategy toward the synthesis of 4-substituted 1*H*-1,2,3-triazoles linked to a fused, bis-heterocyclic peptidomimetic. A key aspect of this work is that the cascade IMCR process rapidly generates a complex alkyne for the subsequent [3+2] cycloaddition (Scheme 2).

## Results and Discussion

**Scheme 3:**
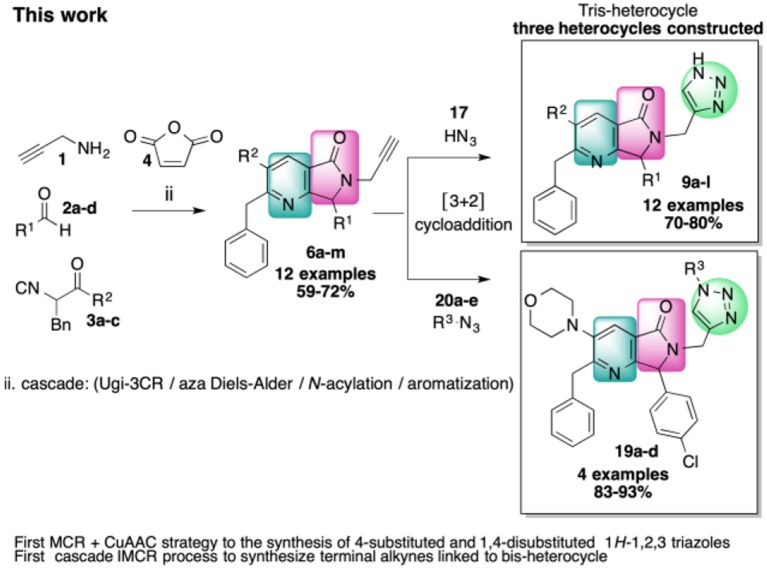
Synthesis of 4-substituted 1*H*-1,2,3-triazoles **9a-l** and 1,4-disubstituted 1*H*-1,2,3-triazoles **19a-l** via an IMCR/aza Diels-Alder/*N*-acylation/aromatization/[3+2] cycloaddition sequence.

In this work, we report the two-step synthesis of compounds **9a**-**m** and **19a-d**, which contain three different heterocycles: pyridine, pyrrolidin-2-one, and 1*H*-1,2,3-triazole (4-substituted and 1,4-disubstituted) (Scheme 3). The use of orthogonal, bifunctional reagents plays a central role in the IMCR/post-transformation strategy, leading to the rapid generation of molecular complexity in both bis-heterocycles **6a**-**m** and final products **9a**-**l**. In the first step, the synthesis of **6a**-**m** occurs by a cascade process combining an Ugi-3CR with aza Diels-Alder, *N*-acylation, and aromatization reactions to give a complex terminal alkyne functionalized at the α-position with a fused bis-heterocycle. Two fused rings were created in the process, resulting in pyrrolo[3,4-*b*]pyridin-5-ones **6a**-**m**.

To optimize the cascade process, we attempted to synthesize complex alkyne **6a** by sequentially reacting propargylamine (**1**), benzaldehyde (**2a**), isocyanide **3a**, and maleic anhydride (**4**) using toluene (Table 1). Performing the reaction without catalyst at 60–80 °C under conventional conditions produced **6a** in 13% yield (entry 1, Table 1). When the reaction was carried out using catalytic amounts of NH_4_Cl at 60–80 °C (Janvier et al., 2002), the product was isolated in 43% yield (entry 2, Table 1). Under microwave conditions, the yield increased to 57% (entry 3, Table 1). In previous reports, we used TsOH for the aromatization process after a Diels-Alder cycloaddition to construct the isoindolin-1-one moiety (Rentería-Gómez et al., 2016a). Unfortunately, bis-heterocycle **6a** was not detected when TsOH was used; only decomposition was observed by TLC.

**Table 1:** IMCR based cascade strategy screening conditions.

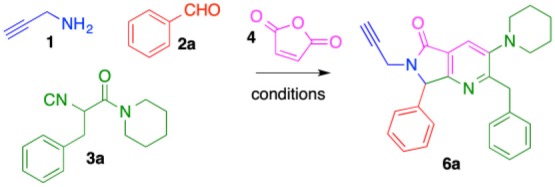					
**Entry^a^**	**Solvent^b^**	**Additive**	**T (^**°**^C)**	**t (h)**	**Yield (%)^g^**
1	PhMe	——-	65–80	12	13
2	PhMe	NH_4_Cl^c^	65–80	12	43
3	PhMe	NH_4_Cl^c^	65–80^d^	1.5	57
4	PhMe	TsOH^e^	65-80^d^	1.5	—
5	PhMe	Sc(OTf)_3_^f^	65–80	12	42
**6**	**PhMe**	**Sc**${\left(OTf\right)}_{3}^{f}$	**65–80**^d^	**1.5**	**69**
7	PhMe	Yb(OTf)_3_^f^	65-80^d^	1.5	62

a *1.0 equiv. **1**, 1.0 equiv. **2a**, 1.2 equiv. **3a**, 1.4 equiv. **4***.

b *1.0 mL solvent*.

c *1.4 equiv*.

d *MW (100 W)*.

e *1.5 equiv*.

f *3.0 mol%*.

g *isolated products*.

*Bold values indicate the best conditions*.

Using catalytic Sc(OTf)_3_ under conventional heating, **6a** was obtained in 42% yield (entry 5, Table 1). Microwave heating with Sc(OTf)_3_ or Yb(OTf)_3_ (Islas-Jácome et al., 2011, 2012) raised the yield to 69 and 62%, respectively (entries 6-7, Table 1). We therefore used the optimal conditions (entry 6: 3 mol% Sc(OTf)_3_, microwave heating 60–80 °C, 1.5 h) to synthesize the series of fused bis-heterocycles **6a**-**l** (Table 2).

**Table 2:** Synthesis of the 6-propargyl-pyrrolo[3,4-*b*]pyridin-5-ones **5a-l**.

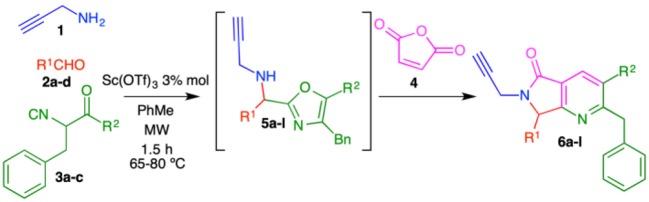			
**Product^a^**	**R^**1**^**	**R^**2**^**	**Yield (%)^b^**
**6a**	Ph	piperidine	64
**6b**	3,4-diOMePh	piperidine	72
**6c**	4-FPh	piperidine	62
**6d**	*n*-hexyl	piperidine	64
**6e**	Ph	morpholine	69
**6f**	3,4-diOMePh	morpholine	66
**6g**	4-FPh	morpholine	66
**6h**	*n*-hexyl	morpholine	67
**6i**	Ph	diethylamine	59
**6j**	3,4-diOMePh	diethylamine	63
**6k**	4-FPh	diethylamine	64
**6l**	*n*-hexyl	diethylamine	66

a *Reactions performed with 1.0 equiv. propargylamine (**1**), 1.0 equiv. aldehyde **2a-d**, 1.2 equiv. isocyanide **3a-c**, 1.4 equiv. maleic anhydride (**4**), 3 mol% Sc(OTf)_3_, 1 mL PhMe*.

b *isolated products*.

The scope of this cascade process was explored using alkyl and aryl aldehydes **2a**-**d** and amide-containing isocyanides **3a**-**c** (Table 2). The role of fluorine atoms in improving bioavailability, lipophilicity and metabolic resistance in bioactive molecules is well-documented (Purser et al., 2008). The products **6c**, **6g**, and **6k** containing fluorine atom was synthesized. Piperidine, morpholine, and diethylamine were incorporated as substituents of isocyanides **3a-c**. These fragments can act as structural bioisosteres, preferably interacting with some amino acids allowing in some cases improve biological activity (Kalinsky and Weinstein, 1954; Sander et al., 2008; Meng et al., 2011; El-Nassan, 2015; Yu et al., 2015; Sato et al., 2017).

**Scheme 4:**
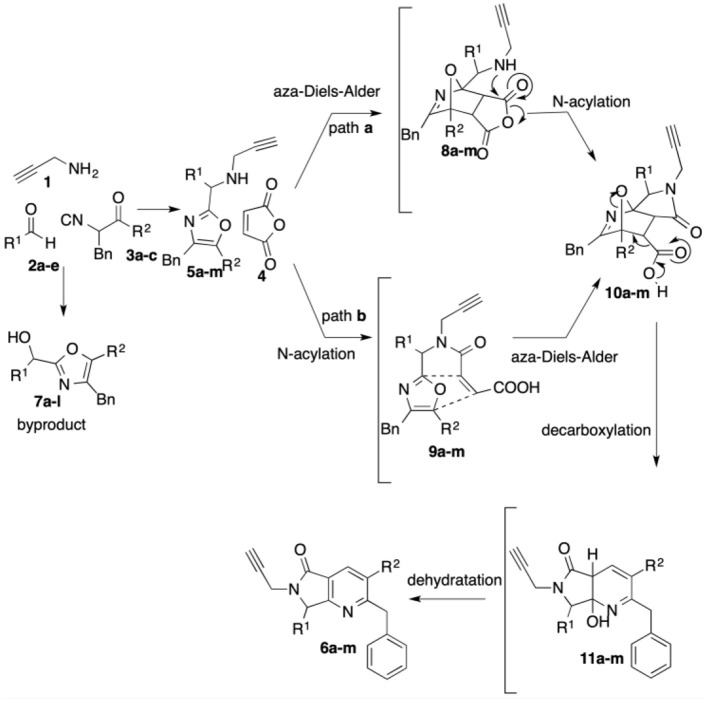
Plausible reaction mechanism.

The highest yield (72%) was obtained for product **6b**, which contains 2,3-dimethoxyphenyl and piperidine as substituents at R^1^ and R^2^, respectively. Contrarily, bis-heterocycle **6i**, with phenyl and diethylamine substituents, was obtained in the lowest yield (59%). In fact, among all products, the diethylamine-containing analogs **6i**-**l** were synthesized in lower yields, which can be attributed to the lower stability of this isocyanide in acidic media. In all cases, the primary byproducts were the corresponding 5-aminooxazoles resulting from Lewis-acid-catalyzed chain-ring tautomerization of the isocyanides **3a**-**c** (Gao et al., 2016). Consistent with reports by Zhu (Cuny et al., 2004; Wang et al., 2007), we also observed, as minor byproducts, the alcohols resulting from isocyanide addition to the aldehydes prior to oxazole formation (**7)** (Scheme 4). The plausible reaction mechanism for the formation of pyrrolo[3,4-*b*]pyridin-5-ones **6a**-**l** is supported by computational calculations performed previously using DFT methods (Scheme 4) (Islas-Jácome et al., 2016).

Conditions were screened for the [3+2] cycloaddition using terminal alkyne **6a** (Table 3). Heating at 100 °C for 12 h with 1.0 equiv. TMSN_3_ and 3 mol% CuI provided tris-heterocycle **9a** in a modest 48% yield (entry 1, Table 2). Increasing the equivalents of the volatile TMSN_3_ to 1.5 or 2.0 raised the yield of **9a** to 69 and 77%, respectively (entries 2-3, Table 2). Using additional CuI (5% mol) and increasing the reaction time to 18 h did not improve the yield (entry 4, Table 2). Microwave heating reduced both the reaction time and the yield to 52%, as high amounts of byproducts were detected (entry 5, Table 2).

**Table 3:** Screening conditions for the [3+2] cycloaddition.

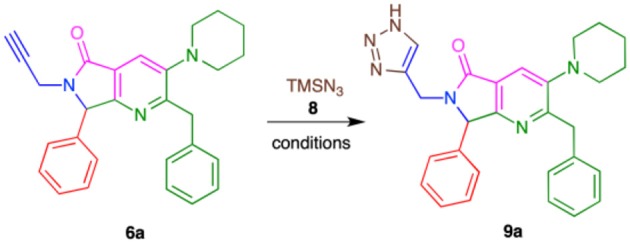					
**Entry**	**TMSN_**3**_ (equiv.)**	**CuI (mol%)**	**T (^**°**^C)**	**t (h)**	**Yield (%)^a^**
1	1.0	3	100	12	48
2	1.5	3	100	12	69
**3**	**2.0**	**3**	**100**	**12**	**77**
4	2.0	5	100	18	75
5	2.0	3	100^b^	0.5	52

a *isolated products*.

b *MW (100 W)*.

*Bold values indicate the best conditions*.

Using the optimized conditions, a series of tris-heterocycles (**9a**-**l**) was synthesized from complex alkynes **6a**-**I** via the [3+2] cycloaddition in good yields (70–80%, Table 4). The highest yields were obtained for the 4-fluorophenyl analogs. Alkynes **6a**-**l** and triazole products **9a**-**l** were fully characterized by IR, ^1^H and ^13^C NMR, and HRMS (see the Supplementary Material for further details). Several attempts to obtain adequate crystals for X-ray analysis were performed without success.

**Table 4:** Synthesis of 4-substituted 1*H*-1,2,3-triazoles **9a-l**.

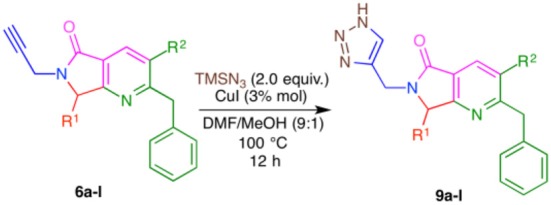			
**Product^a^**	**R^**1**^**	**R^**2**^**	**Yield (%)^b^**
**9a**	Ph	piperidine	77
**9b**	3,4-diOMePh	piperidine	75
**9c**	4-FPh	piperidine	80
**9d**	*n*-hexyl	piperidine	70
**9e**	Ph	morpholine	63
**9f**	3,4-diOMePh	morpholine	73
**9g**	4-FPh	morpholine	80
**9h**	*n*-hexyl	morpholine	75
**9i**	Ph	diethylamine	73
**9j**	3,4-diOMePh	diethylamine	70
**9k**	4-FPh	diethylamine	78
**9l**	*n*-hexyl	diethylamine	75

a *1.0 equiv. alkyne **6a**-**l**, 2.0 equiv. TMSN_3_, 3% mol CuI in MeOH/DMF (9:1 v/v, 0.5 M) at 100 °C for 12 h*.

b *isolated products*.

**Scheme 5:**
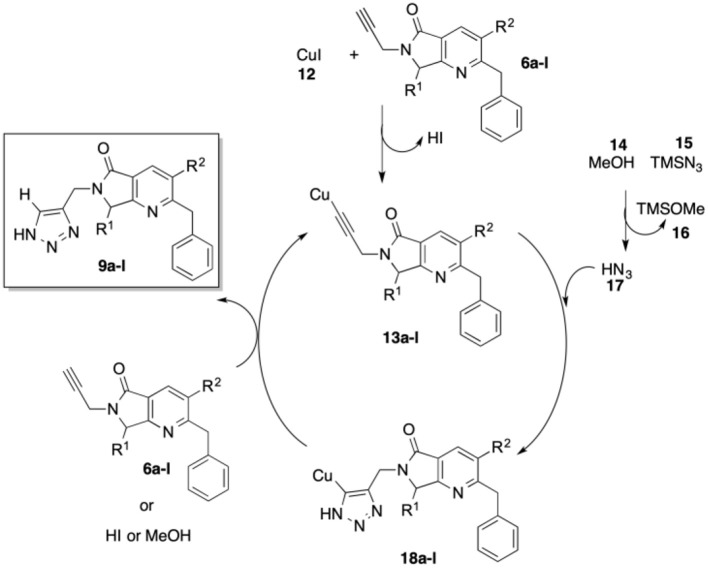
Plausible CuAAC reaction mechanism.

In Scheme 5, we show a plausible reaction mechanism for the alkyne-azide [3+2] cycloaddition to produce 4-substituted 1*H*-1,2,3-triazoles. The reaction likely proceeds through the formation of copper acetylide species **13a**-**l** from terminal alkynes **6a**-**l** with CuI (**12**) and *in situ* generation of HN_3_ (**17**) from the reaction of TMSN_3_ (**15**) and MeOH (**14**). Copper-assisted cycloaddition between **13a**-**l** and HN_3_ (**17**) takes place to form intermediates **18a**-**l**. Protonolysis of the C-Cu bond of **18a**-**l** by terminal alkynes **6a**-**l**, HI, or MeOH affords 4-substituted 1*H*-1,2,3-triazoles **9a**-**l** (Jin et al., 2004).

Having the methodology for the synthesis of compounds **9a-l**, we next explored the scope of use de terminal alkynes linked to bis-heterocycles using organic azides to obtain the 1,4-disubstituted 1,2,3-triazoles (**19a-d**) via CuAAC. Compound **6m** was synthetized (60% yield) and selected as model. Phenyl azides with different stereo-electronic natures (**20a-d**) were prepared from aromatic amines via diazotization with sodium nitrite in water in the presence of p-TsOH followed by reaction with sodium azide at room temperature (Kutonova et al., 2013).

First, the reaction was carried out under constant stirring, at room temperature, using **6m** and azide **20a** obtaining an 85% yield of 1,4-disubstituted 1,2,3-triazole **19a** after 5 h. When the reaction was carried out using ultrasound-assisted irradiation (USI) at room temperature, the product **19a** was obtained in 1.5 h, with a yield of 83%. For this reason, we decided to use the USI protocol for the synthesis of 1,4-disubstituted 1-*H*-1,2,3-triazoles (**19a-d**). The reactions under USI resulted in reduced reaction times (30–90 min) and good yields (83–93%) in the CuAAC for the synthesis of **19a-e** (Table 5).

**Table 5:** Synthesis of 1,4-disubstituted 1*H*-1,2,3-triazoles **19a-e**.

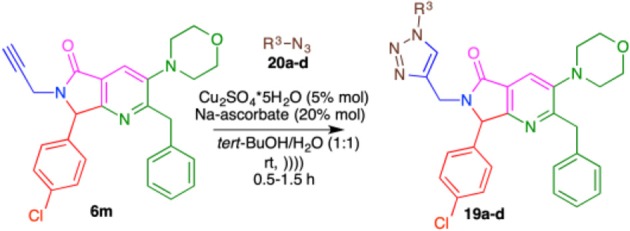			
**Product^a^**	**R^**3**^**	**t (min)**	**Yield (%)^b^**
**19a**	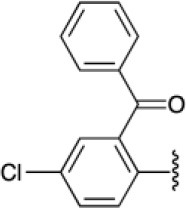	90	83
**19b**	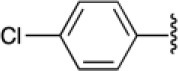	30	89
**19c**	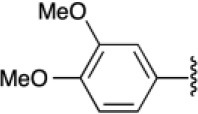	45	93
**19d**	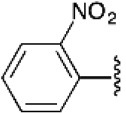	45	90

a 1.0 equiv. azide **20a**-**e**, 5% mol Cu_2_${\text{SO}}_{4}^{*}$5H_2_O, 20% mol Na-ascorbate in H_2_O/tert-BuOH (1:1 v/v, 0.3 M) at rt (USI) for 30-90 min.

b *isolated products*.

## Conclusions

We have developed a new and efficient strategy to synthesize 4-substituted and 1,4-disubstituted 1*H*-1,2,3-triazoles linked to pyrrolo[3,4-*b*]pyridin-5-ones. Molecules containing these heterocycles together are novel. The molecules synthesized contain privileged tris-heterocycles which could have applications in medicinal chemistry and coordination chemistry.

The IMCR based cascade process coupled with CuAAC strategy, as convergent and powerful tool toward the synthesis of bis and tris heterocycles is unreported.

## Data Availability

All datasets generated for this study are included in the manuscript and/or the Supplementary Files.

## Author Contributions

MR-G, RG-M, and DV have made a substantial, direct and intellectual contribution to the work. SP was responsible for performing the initial experiments. AI-J was responsible for designing and analyzing the results. All authors discussed the whole project, wrote the publication, and approved it for publication.

### Conflict of Interest Statement

The authors declare that the research was conducted in the absence of any commercial or financial relationships that could be construed as a potential conflict of interest.
