# Supplementary material for: Synthesis of Tris-Heterocycles via a Cascade IMCR/Aza Diels-Alder + CuAAC Strategy
Source: Front Chem. 2019 Aug 6;7:546. doi: 10.3389/fchem.2019.00546 (PMC6691067; doi:10.3389/fchem.2019.00546)
Supplement: Supplementary file 1 [file Data_Sheet_1.pdf]

SUPPORTING INFORMATION

Synthesis of tris-heterocycles via a cascade IMCR/Diels-Alder + CuAAC strategy

Manuel A. Rentería-Gómez [1], Alejandro Islas-Jácome [2], Shrikant G. Pharande [1], David A. Vosburg [1,3], Rocío Gámez-Montaño [1*]

[1]Departamento de Química, Universidad de Guanajuato, Guanajuato, México
[2]Departamento de Química, Universidad Autónoma Metropolitana-Iztapalapa, Ciudad de México, México
[3]Department of Chemistry, Harvey Mudd College, Claremont, California, USA

* rociogm@ugto.mx (R.G.-M.)
* vosburg@hmc.edu (D.A.V.)

Table contents

Index	Page no.
General information	**S2**
General procedure for the Synthesis and characterization of the 6-propargyl-pyrrolo[3,4-*b*]pyridin-5-ones **6a-m** (**GP-1**)	**S2**
^{1}H NMR, ^{13}C NMR and HRMS spectra of **6a-m**	**S3**
General procedure for the synthesis and characterization of 6-((1*H*-1,2,3-triazol-4-yl)methyl)-pyrrolo[3,4-*b*]pyridin-5-ones **9a-l** (**GP-2**)	**S29**
^{1}H NMR, ^{13}C NMR and HRMS spectra of **9a-l**	**S29**
General procedure for the synthesis and characterization of 6-((1*H*-1,2,3-triazol-4-yl)methyl)-pyrrolo[3,4-*b*]pyridin-5-ones **19a-d** (**GP-3**)	**S55**
^{1}H NMR, ^{13}C NMR and HRMS spectra of **19a-d**	**S55**

General Information. ^{1}H and ^{13}C NMR spectra were acquired on Bruker Advance III spectrometers (500 or 400 MHz). The solvent used was deuterated chloroform ($CDCl_3$). Chemical shifts are reported in parts per million (δ/ppm). The internal reference for ^{1}H NMR spectra is tetramethylsilane (TMS) at 0.0 ppm. The internal reference for ^{13}C NMR spectra is $CDCl_3$ at 77.0 ppm. Coupling constants are reported in Hertz (*J*/Hz). Multiplicities of signals are reported using the standard abbreviations: singlet (s), doublet (d), triplet (t), quartet (q) and multiplet (m). NMR spectra were analyzed using MestreNova software version 10.0.1-14719. IR spectra were acquired on a Perkin Elmer 100 spectrometer. The absorbance peaks are reported in reciprocal centimeters (υ_{max}/cm^{-1}). HRMS spectra were acquired on a Bruker Daltonics Maxis Impact ESI-qTOF MS spectrometer. HRMS samples were ionized by ESI^{+} mode and recorded via the TOF method. Microwave-assisted reactions were performed on a CEM Discover™ Synthesis Unit in closed-vessel mode. Ultrasound irradiated reactions were performed in vials placed into a water bath of a Branson 1510 sonicator cleaner working at 42 kHz ± 6% frequencies. Reaction progress was monitored by thin-layer chromatography (TLC) on precoated silica gel Kieselgel 60 F254 plates and spots were visualized under UV light at 254 or 365 nm. Mixtures of hexanes with ethyl acetate (EtOAc) were used as eluents for TLC and for measuring retention factors (R_f). Flash column chromatography was performed using silica gel (230-400 mesh) and mixtures of hexanes with EtOAc in different proportions (v/v) as mobile phase. Melting points were determined on an electrothermal apparatus and were uncorrected. All starting materials were purchased from Sigma-Aldrich and were used without further purification. Chemical names and drawings were obtained using the ChemBioDraw Ultra 13.0.2.3020 software package. The purity for all the synthesized compounds (up to 99%) was assessed by NMR.

General procedure for the *Synthesis and characterization of the 6-propargyl-pyrrolo[3,4-b]pyridin-5-ones* 6a-m (GP-1): Propargylamine (**1**) (1.0 equiv.) and the corresponding aldehyde **2** (1.0 equiv.) were placed in a 10 mL sealed CEM Discover™ microwave reaction tube and diluted in 1.0 mL of dry toluene. The mixture was then irradiated (MW, 55 W, 65 °C) for 20 min and $Sc(OTf)_3$ (3% mol) was added. The mixture was irradiated (MW, 55 W, 65 °C) for 15 min, and the corresponding isocyanide **3** (1.2 equiv.) was added. The mixture was irradiated (MW, 65 W, 80 °C), but this time for 30 min, and maleic anhydride (**4**) (1.4 equiv.) was added. Finally, this reaction mixture was irradiated (MW, 65 W, 80 °C) for 30 min. Then, the solvent was removed to dryness under vacuum. The residue was diluted in dichloromethane (5.0 mL) and washed with a concentrated aq. solution of $NaHCO_3$ (3 × 25 mL) and brine (3 × 25 mL). The organic layer was dried with Na_2SO_4 and filtered over a pad of celite. The solvent was removed to dryness under vacuum. The crude product was purified by flash chromatography to afford the corresponding pyrrolo[3,4-*b*]pyridin-5-ones **6a-m**.

2-Benzyl-7-phenyl-3-(piperidin-1-yl)-6-(prop-2-yn-1-yl)-6,7-dihydro-5*H*-pyrrolo[3,4-*b*]pyridin-5-one (6a): According to GP-1, propargylamine (47.0 µL, 0.726 mmol), benzaldehyde (74.0 µL, 0.726 mmol), scandium (III) triflate (11.0 mg, 0.021 mmol), 2-isocyano-3-phenyl-1-(piperidin-1-yl)propan-1-one (211.0 mg, 0.871 mmol), and maleic anhydride (99.0 mg, 1.02 mmol) were reacted together in dry toluene (1.0 mL) to afford the product **6a**. Yield 64% (195.0 mg); yellow oil; $\boldsymbol{R_f}$ **=** 0.35 (hexanes/EtOAc, 7:3); **FT-IR (ATR)** υ_{max}/cm^{-1} 2114 (C≡C), 1658 (C=O); **^{1}H NMR** (500 MHz, $CDCl_3$, 25 °C): δ = 1.52–1.61 (m, 2H), 1.64–1.75 (m, 4H), 2.24 (t, *J* = 2.5 Hz, 1H), 2.70–2.82 (m, 4H), 3.51 (dd, *J* = 17.6, 2.5 Hz, 1H), 4.18 (d, *J* = 13.7 Hz, 1H), 4.27 (d, *J* = 13.7 Hz, 1H), 4.93 (dd, *J* = 17.6, 2.5 Hz, 1H), 5.66 (s, 1H), 7.09–7.24 (m, 7H), 7.32–7.40 (m, 3H), 7.82 (s, 1H); **^{13}C NMR** (126 MHz, $CDCl_3$, 25 °C): δ = 23.9, 26.4, 29.6, 39.8, 54.3, 64.5, 72.3, 78.1, 123.3 (2), 126.0, 128.0, 128.1, 128.6, 128.8, 128.9, 135.2, 139.5, 149.4, 159.8, 162.4, 166.7; **HRMS (ESI$^+$):** *m/z* calcd. for $C_{28}H_{28}N_3O^+$ $[M + H]^+$ 422.2227, found 422.2252.

^{1}H NMR **6a**

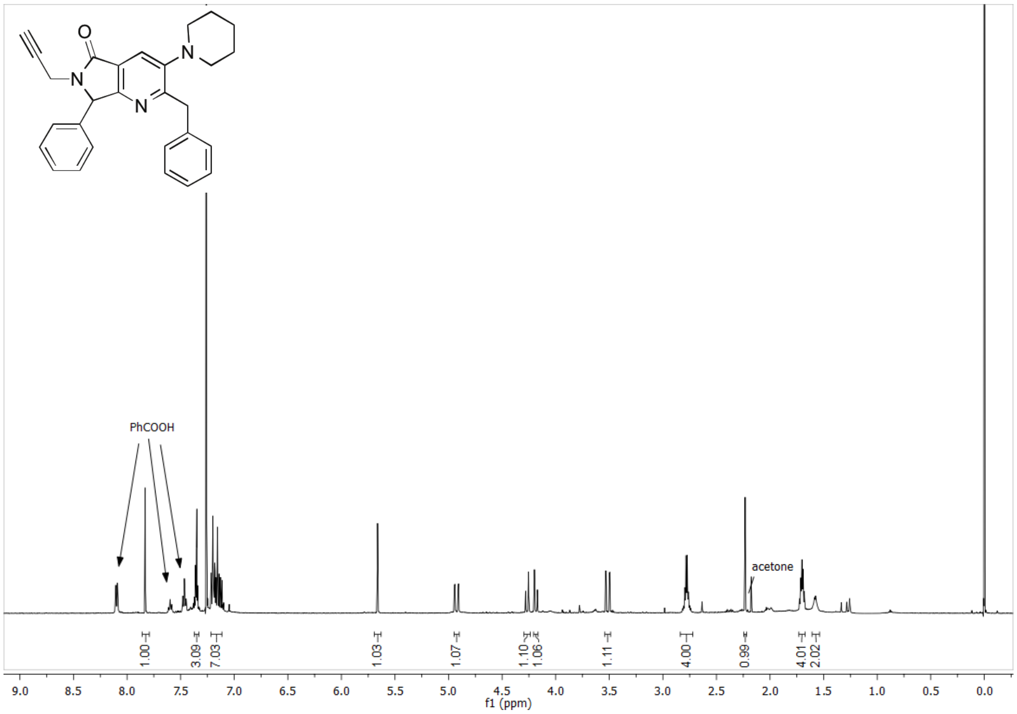

^{13}C NMR **6a**

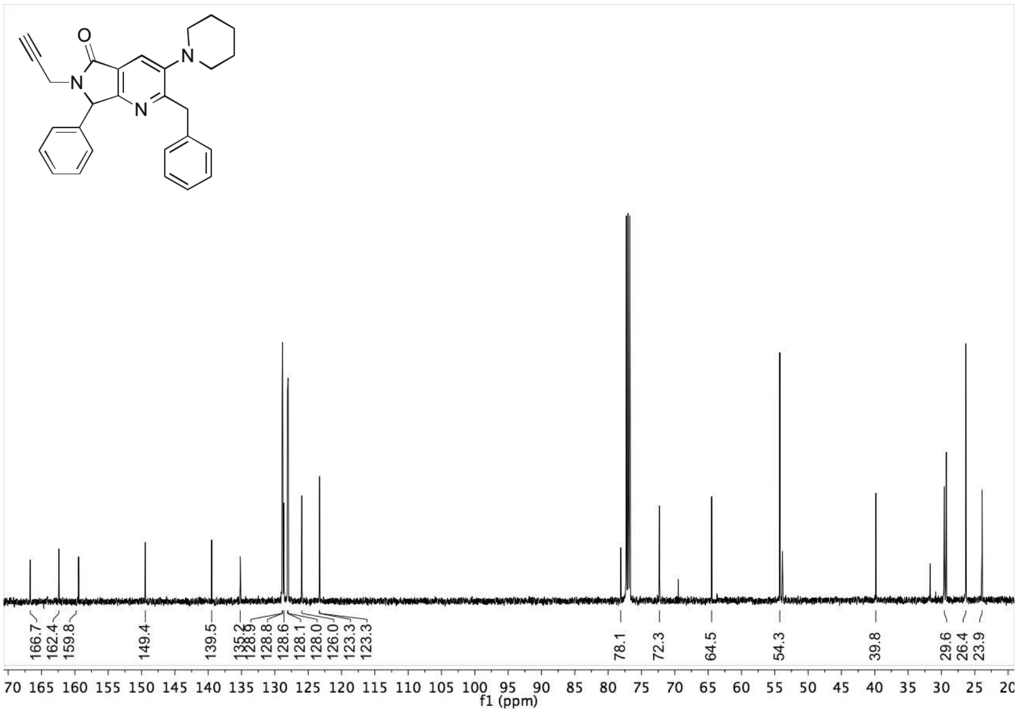

HRMS **6a**

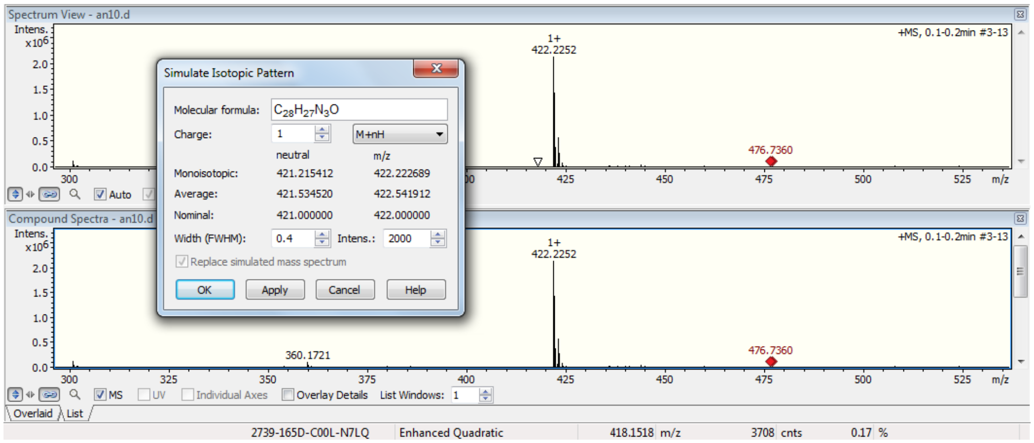

2-Benzyl-7-(3,4-dimethoxyphenyl)-3-(piperidin-1-yl)-6-(prop-2-yn-1-yl)-6,7-dihydro-5*H*-pyrrolo[3,4-*b*]pyridin-5-one (6b): According to GP-1, propargylamine (47.0 µL, 0.726 mmol), 3,4-dimethoxybenzaldehyde (112.0 mg, 0.726 mmol), scandium triflate (11.0 mg, 0.021 mmol), 2-isocyano-3-phenyl-1-(piperidin-1-yl)propan-1-one (211.0 mg, 0.871 mmol), and maleic anhydride (99.0 mg, 1.02 mmol) were reacted together in dry toluene (1.0 mL) to afford the pyrrolo[3,4-*b*]pyridin-5-one **6b**. Yield 72% (211.0 mg); yellow oil; R_f **=** 0.65 (hexanes/EtOAc, 1:1); **FT-IR** (ATR) υ_{max}/cm^{-1} 2118 (C≡C), 1692 (C=O); ^{1}**H NMR** (500 MHz, $CDCl_3$, 25 °C): δ = 1.53–1.62 (m, 2H), 1.68–1.75 (m, 4H), 2.25 (s, 1H), 2.73–2.84 (m, 4H), 3.54 (dd, *J* = 17.6, 2.4 Hz, 1H), 3.75 (s, 3H), 3.88 (s, 3H), 4.22 (d, *J* = 13.6 Hz, 1H), 4.27 (d, *J* = 13.7 Hz, 1H), 4.90 (dd, *J* = 17.5, 2.5 Hz, 1H), 6.56 (s, 1H), 5.60 (s, 1H), 6.82–6.88 (m, 2H), 7.10–7.24 (m, 5H), 7.83 (s, 1H); ^{13}**C NMR** (126 MHz, $CDCl_3$, 25 °C): δ = 23.9, 26.4, 29.6, 39.8, 54.3, 64.5, 72.3, 78.1, 123.3 (2), 126.0, 128.0, 128.1, 128.6, 128.8, 128.9, 135.2, 139.5, 149.4, 159.8, 162.4, 166.7; **HRMS (ESI^{+}):** *m/z* calcd. for $C_{30}H_{32}N_3O_3^+$ $[M + H]^+$ 482.2438, found 482.2435.

^{1}H NMR **6b**

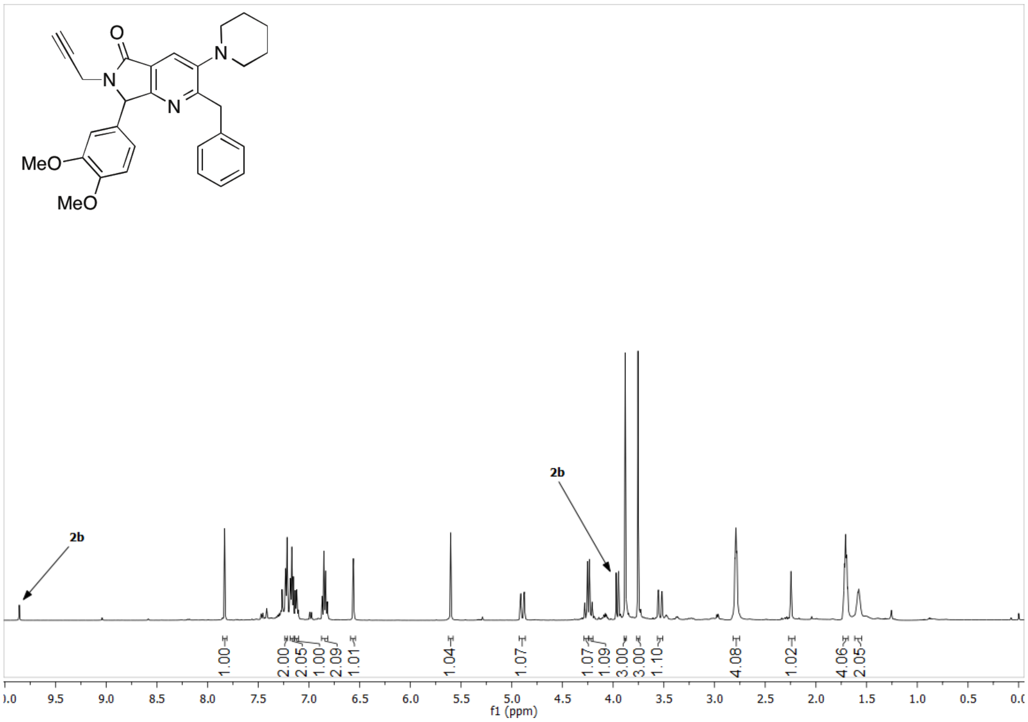

^{13}C NMR **6b**

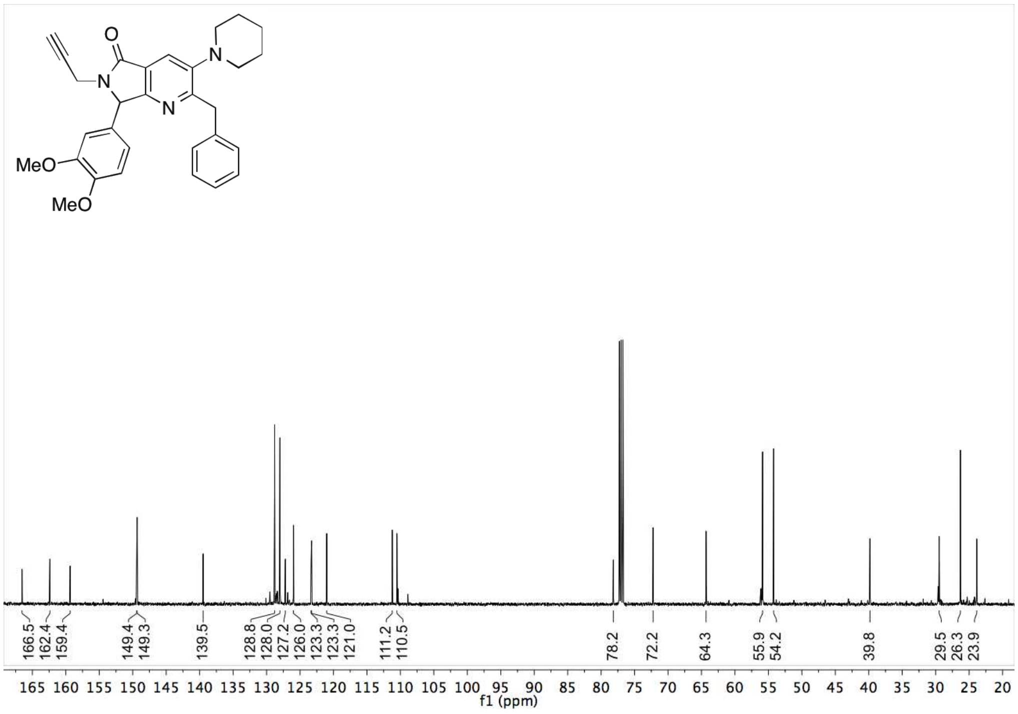

HRMS **6b**

Mass Spectrum SmartFormula Report

Analysis Info

Analysis Name	D:\Data\Monica Rincon\Eduardo_Gonzalez\20170228_5b_Pos.d	Acquisition Date	2/28/2017 12:17:59 PM
Method	tune_low_cres_110117MS.m	Operator	CBS_UAM_I
Sample Name	20170228_5b_Pos	Instrument	micrOTOF 213750.00410
Comment	20170228_5b_Pos		

Acquisition Parameter

Source Type	ESI	Ion Polarity	Positive	Set Nebulizer	3.0 Bar
Focus	Active	Set Capillary	4500 V	Set Dry Heater	210 °C
Scan Begin	50 m/z	Set End Plate Offset	-500 V	Set Dry Gas	10.0 l/min
Scan End	3000 m/z	Set Charging Voltage	0 V	Set Divert Valve	Source
		Set Corona	0 nA	Set APCI Heater	0 °C

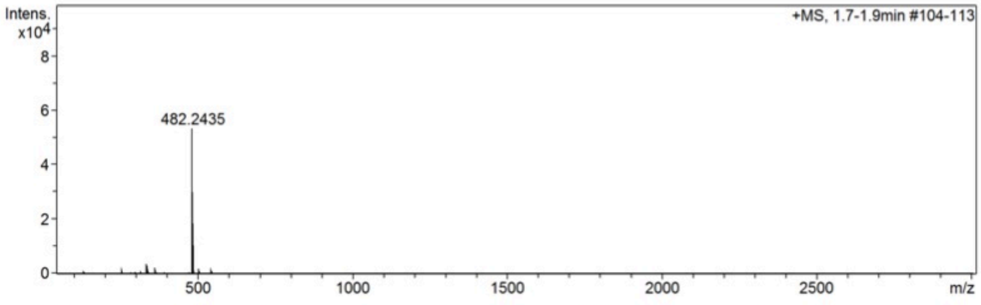

Meas. m/z	#	Ion Formula	m/z	err [ppm]	mSigma	# Sigma	Score	rdb	e⁻ Conf	N-Rule
334.1904	1	C21H24N3O	334.1914	-3.0	35.6	1	100.00	11.5	even	ok
482.2435	1	C30H32N3O3	482.2438	-0.6	4.8	1	100.00	16.5	even	ok

2-Benzyl-7-(4-fluorophenyl)-3-(piperidin-1-yl)-6-(prop-2-yn-1-yl)-6,7-dihydro-5*H*-pyrrolo[3,4-*b*]pyridin-5-one (6c): According to GP-1, propargylamine (47.0 µL, 0.726 mmol), 4-fluorobenzaldehyde (78.0 µL, 0.726 mmol), scandium triflate (11.0 mg, 0.021 mmol), 2-isocyano-3-phenyl-1-(piperidin-1-yl)propan-1-one (211.0 mg, 0.871 mmol), and maleic anhydride (99.0 mg, 1.02 mmol) were reacted together in dry toluene (1.0 mL) to afford the pyrrolo[3,4-*b*]pyridin-5-one **6c.** Yield 62% (229.0 mg); yellow oil; $\boldsymbol{R_f}$ **=** 0.54 (hexanes/EtOAc, 3:2); **FT-IR (ATR)** υ_{max}/cm^{-1} 1696 (C=O); **^{1}H NMR** (500 MHz, $CDCl_3$, 25 °C): δ = 1.55–1.60 (m, 2H), 1.68–1.73 (m, 4H), 2.24 (s, 1H), 2.75–2.82 (m, 4H), 3.51 (dd, *J* = 17.6 Hz, 1H), 4.19 (d, *J* = 13.7, 1H), 4.26 (d, *J* = 13.8, 1H), 4.91 (d, *J* = 17.7 Hz, 1H), 5.64 (s, 1H), 7.03–7.07 (m, 2H), 7.12–7.18 (m, 5H), 7.19–7.22 (m, 2H), 7.82 (s, 1H); **^{13}C NMR** (126 MHz, $CDCl_3$, 25 °C): δ = 23.9, 26.3, 29.5, 39.8, 54.2, 63.7, 72.5, 77.9, 115.8, 116.0, 123.2, 123.3, 126.0, 128.0, 128.8, 129.9 (2), 130.9 (2), 139.4, 149.5, 159.1, 161.9, 162.5, 163.9, 166.5; **HRMS (ESI$^+$):** *m/z* calcd. for $C_{28}H_{27}FN_3O^+$ $[M + H]^+$ 440.2133, found 440.2125.

^{1}H NMR **6c**

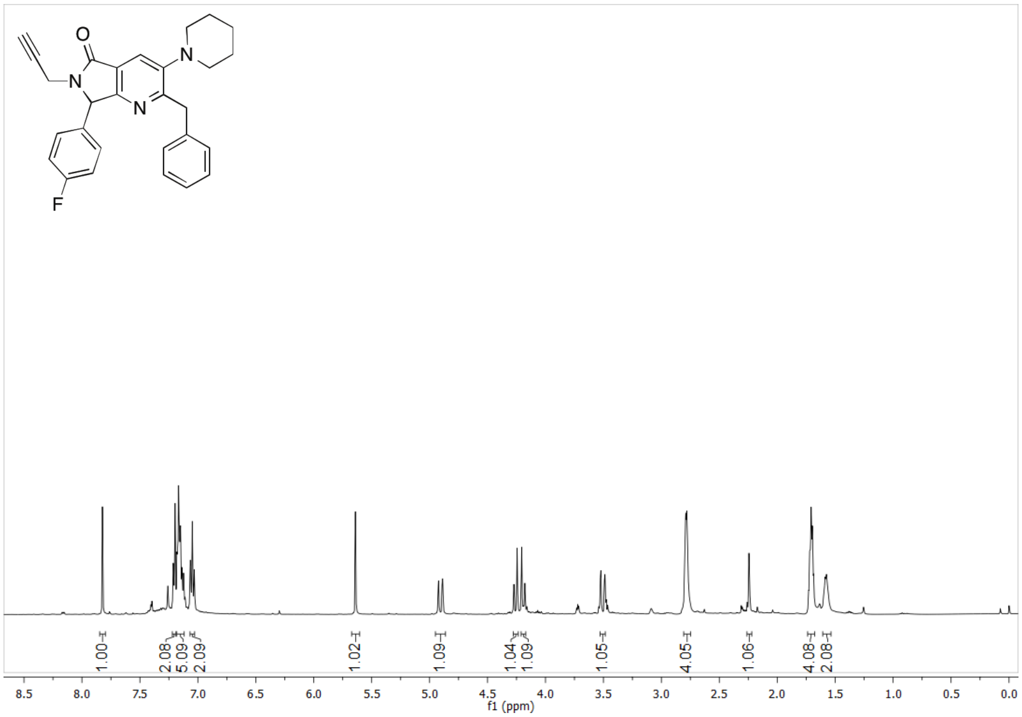

^{13}C NMR **6c**

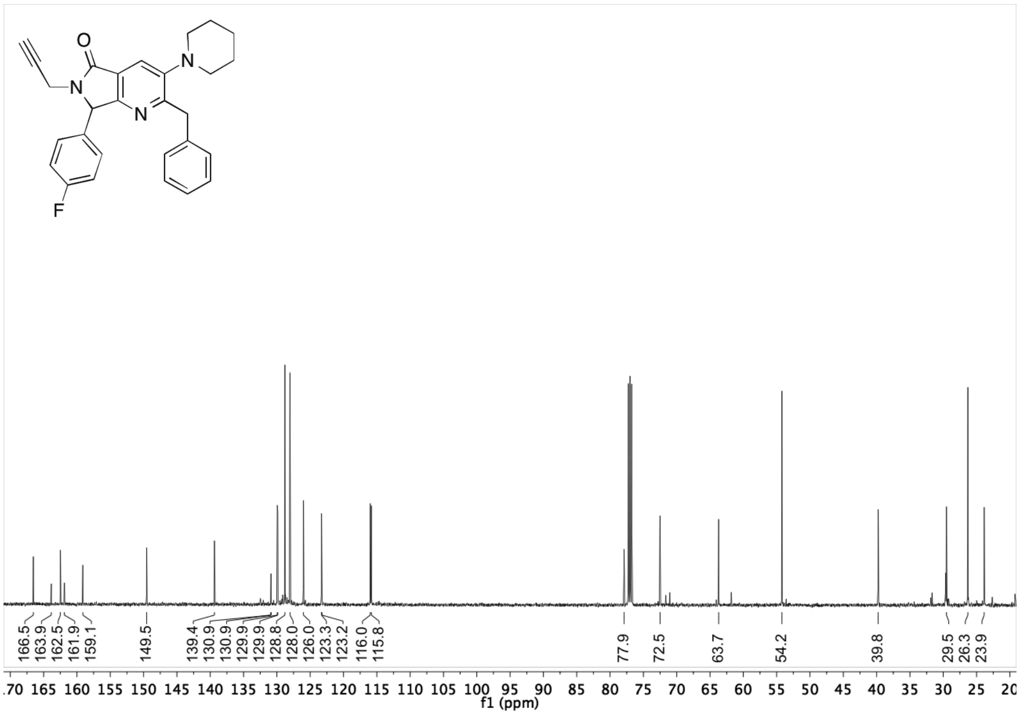

HRMS **6c**

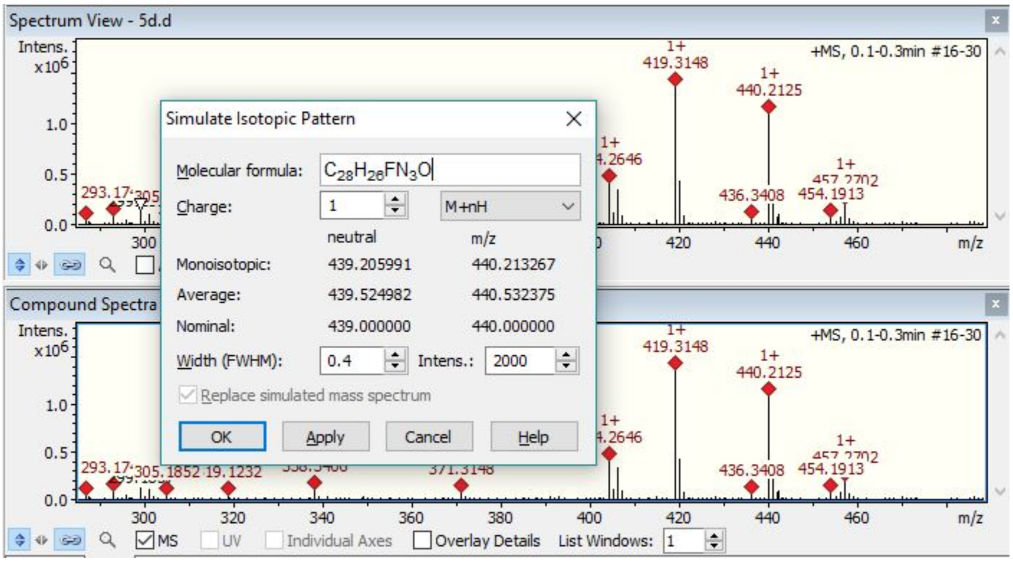

2-Benzyl-7-hexyl-3-(piperidin-1-yl)-6-(prop-2-yn-1-yl)-6,7-dihydro-5*H*-pyrrolo[3,4-*b*]pyridin-5-one (6d): According to GP-1, propargylamine (47.0 µL, 0.726 mmol), heptanaldehyde (102.0 µL, 0.726 mmol), scandium triflate (11.0 mg, 0.021 mmol), 2-isocyano-3-phenyl-1-(piperidin-1-yl)propan-1-one (211.0 mg, 0.871 mmol), and maleic anhydride (99.0 mg, 1.02 mmol) were reacted together in dry toluene (1.0 mL) to afford the pyrrolo[3,4-*b*]pyridin-5-one **6d**. Yield 64% (199.0 mg); yellow oil; $\boldsymbol{R_f}$ = 0.33 (hexanes/EtOAc, 4:1); **FT-IR (ATR)** υ_{max}/cm^{-1} 2118 (C≡C), 1699 (C=O); **^{1}H NMR** (400 MHz, $CDCl_3$, 25 °C): δ = 0.78–0.88 (m, 4H), 1.14–1.21 (m, 7H), 1.55–1.61 (m, 2H), 1.68–1.75 (m, 4H), 1.90–1.99 (m, 1H), 2.16–2.23 (m, 1H), 2.24 (s, 1H), 2.72–2.85 (m, 4H), 3.93 (d, J = 17.7 Hz, 1H), 4.24 (d, J = 13.9 Hz, 1H), 4.40 (d, J = 13.9 Hz, 1H), 4.68–4.75 (m, 1H), 4.85 (d, J = 17.8 Hz, 1H), 7.15–7.33 (m, 5H), 7.77 (s, 1H); **^{13}C NMR** (101 MHz, $CDCl_3$, 25 °C): δ = 14.0, 22.5, 22.6, 23.9, 26.3, 29.1, 29.3, 29.6, 31.5, 39.7, 54.2, 60.2, 72.1, 77.9, 123.1, 123.9, 126.0, 128.1, 128.9, 139.7, 149.1, 159.3, 161.6, 166.8; **HRMS (ESI^{+}):** *m/z* calcd. for $C_{28}H_{36}N_3O^+$ $[M + H]^+$ 430.2853, found 430.2886.

^{1}H NMR **6d**

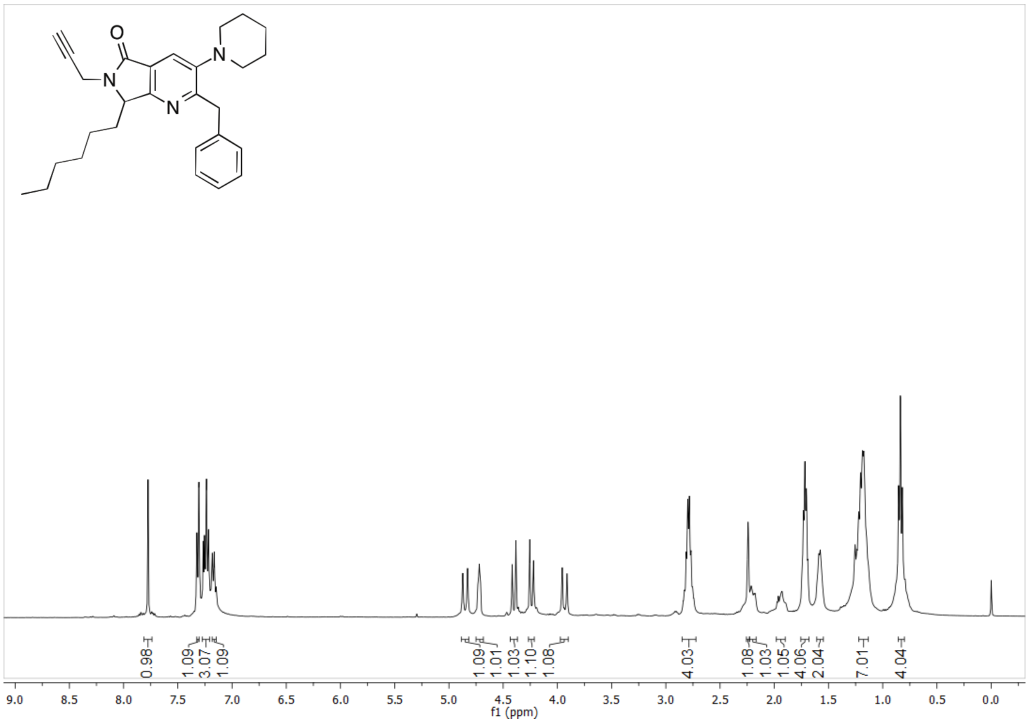

^{13}C NMR **6d**

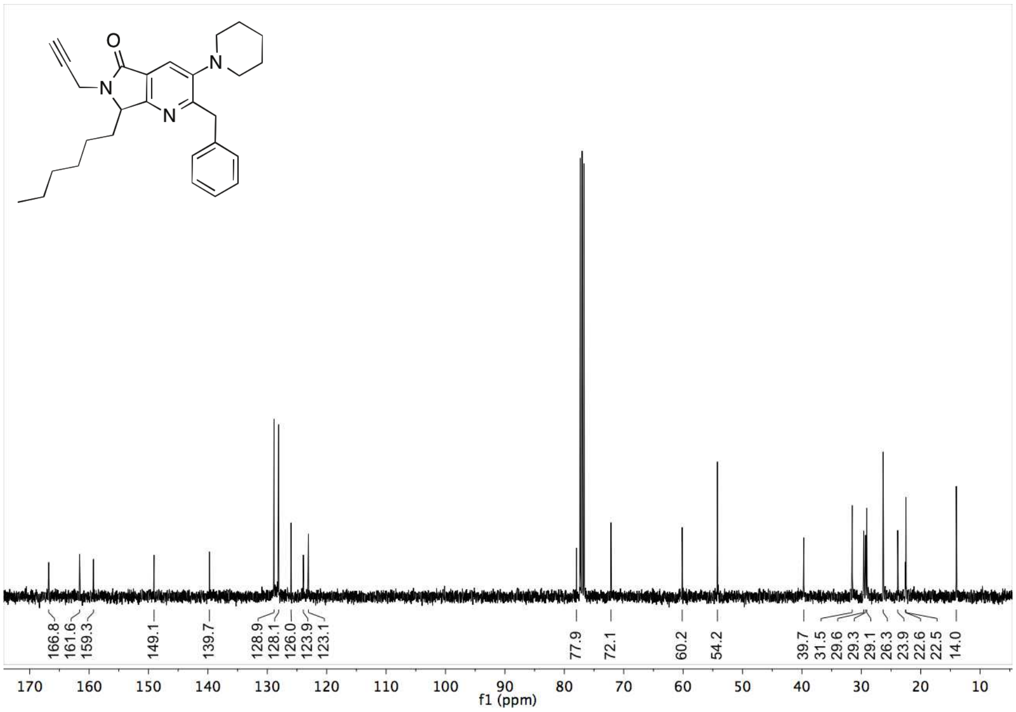

HRMS **6d**

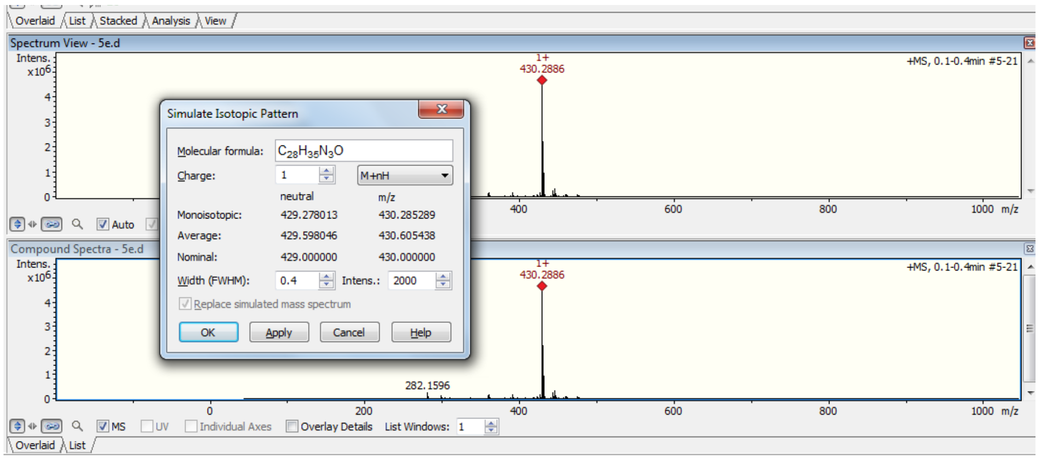

2-Benzyl-3-morpholino-7-phenyl-6-(prop-2-yn-1-yl)-6,7-dihydro-5*H*-pyrrolo[3,4-*b*]pyridin-5-one (6e): According to GP-1, propargylamine (47.0 µL, 0.726 mmol), benzaldehyde (74.0 µL, 0.726 mmol), scandium triflate (11.0 mg, 0.021 mmol), 2-isocyano-1-morpholino-3-phenylpropan-1-one (213.0 mg, 0.871 mmol), and maleic anhydride (99.0 mg, 1.02 mmol) were reacted together in dry toluene (1.0 mL) to afford the pyrrolo[3,4-*b*]pyridin-5-one **6e**. Yield 69% (211.0 mg); yellow oil; $\boldsymbol{R_f}$ = 0.34 (hexanes/EtOAc, 3:2); **FT-IR (ATR)** υ_{max}/cm^{-1} 2119 (C≡C), 1697 (C=O); ^{1}H NMR (500 MHz, $CDCl_3$, 25 °C): δ = 2.26 (t, *J* = 2.5 Hz, 1H), 2.77–2.84 (m, 4H), 3.52 (dd, *J* = 17.6, 2.5 Hz, 1H), 3.77–3.80 (m, 4H), 4.21 (d, *J* = 13.8 Hz, 1H), 4.30 (d, *J* = 13.9 Hz, 1H), 4.93 (dd, *J* = 17.6, 2.6 Hz, 1H), 5.69 (s, 1H), 7.11–7.17 (m, 5H), 7.33–7.37 (m, 3H), 7.89 (s, 1H); ^{13}**C NMR** (126 MHz, $CDCl_3$, 25 °C): δ = 29.5. 39.9, 52.9, 64.4, 66.9, 72.4, 77.9, 123.5, 123.8, 126.0, 127.9, 128.0, 128.6, 128.8, 134.9, 139.1, 147.8, 160.2, 162.2, 166.2; **HRMS (ESI^{+}):** *m/z* calcd. for $C_{27}H_{26}N_3O_2^{+}$ $[M + H]^{+}$ 424.2019, found 424.2049.

^{1}H NMR **6e**

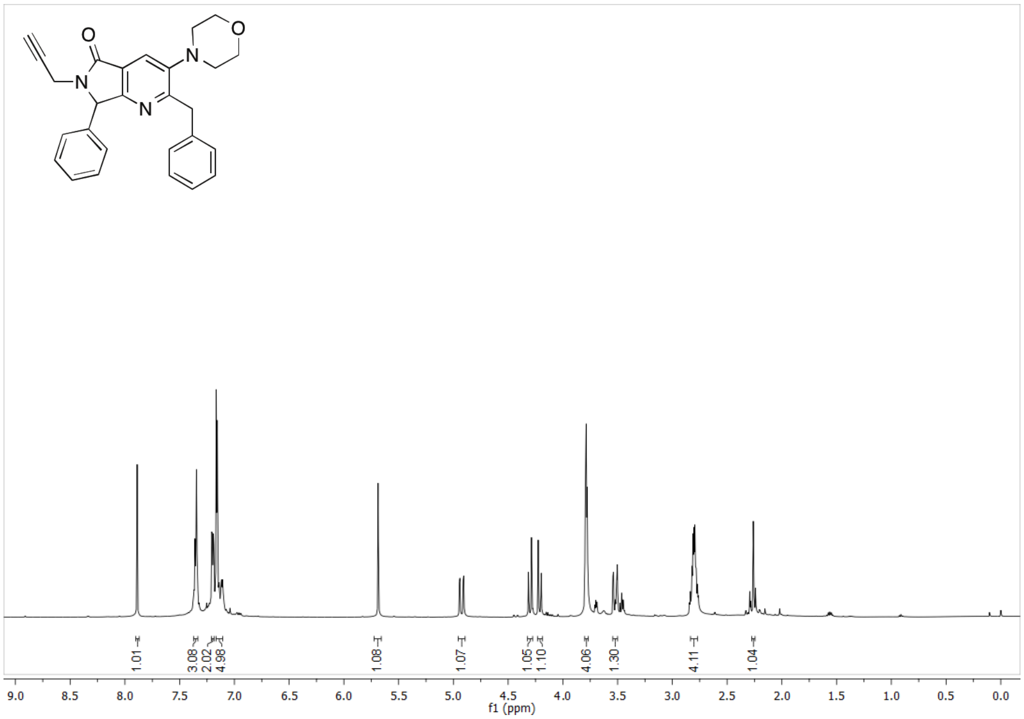

^{13}C NMR **6e**

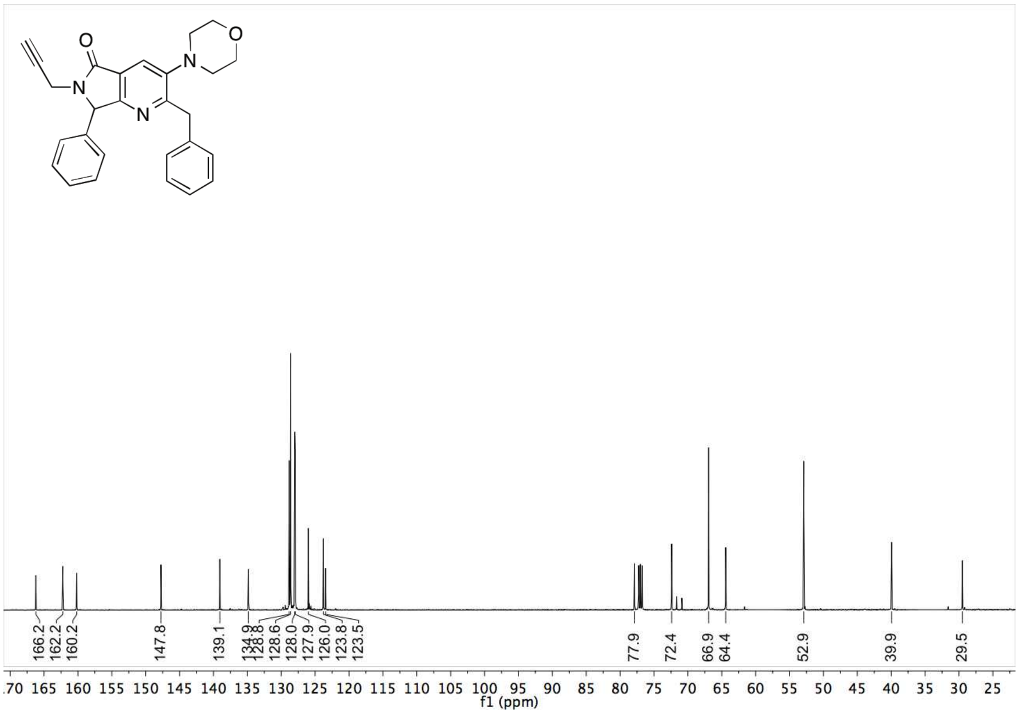

HRMS **6e**

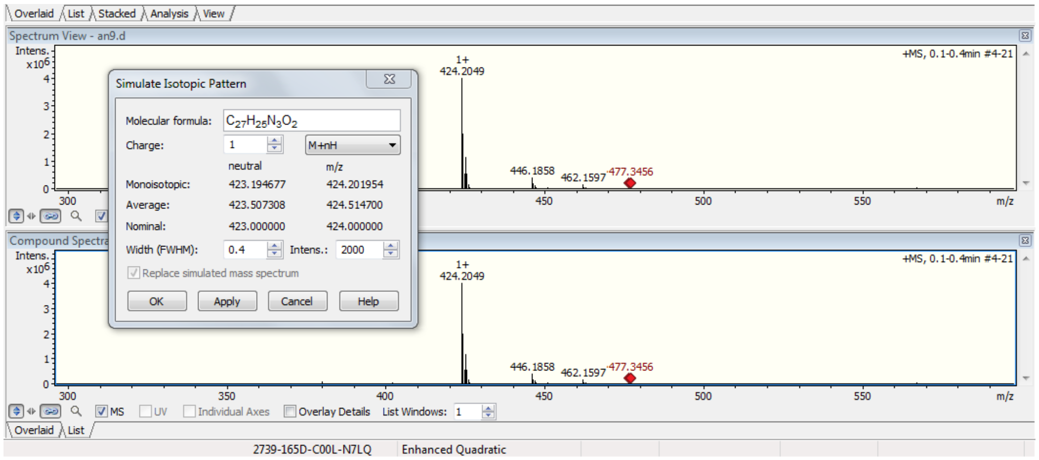

2-Benzyl-7-(3,4-dimethoxyphenyl)-3-morpholino-6-(prop-2-yn-1-yl)-6,7-dihydro-5*H*-pyrrolo[3,4-*b*]pyridin-5-one (6f): According to GP-1, propargylamine (47.0 µL, 0.726 mmol),3,4-dimethoxybenzaldehyde (121.0 mg, 0.726 mmol), scandium triflate (11.0 mg, 0.021 mmol), 2-isocyano-1-morpholino-3-phenylpropan-1-one (213.0 mg, 0.871 mmol), and maleic anhydride (99.0 g, 1.02 mmol) were reacted together in dry toluene (1.0 mL) to afford the pyrrolo[3,4-*b*]pyridin-5-one **6f**. Yield 66% (231.0 mg); yellow oil; $\boldsymbol{R_f}$ = 0.54 (hexanes/EtOAc, 1:1); **FT-IR (ATR)** υ_{max}/cm^{-1} 2127 (C≡C), 1699 (C=O); **^{1}H NMR** (400 MHz, $CDCl_3$, 25 °C): δ = 2.26 (s, 1H), 2.78–2.86 (m, 4H), 3.55 (d, *J* = 17.5 Hz, 1H), 3.78 (s, 3H), 3.80–3.83 (m, 4H), 3.89 (s, 3H), 4.25 (d, *J* = 13.8 Hz, 1H), 4.31 (d, *J* = 13.1 Hz, 1H), 4.92 (d, *J* = 17.6 Hz, 1H), 5.64 (s, 1H), 6.58 (s, 1H), 6.81–6.91 (m, 2H), 7.12–7.22 (m, 5H), 7.89 (s, 1H); **^{13}C NMR** (101 MHz, $CDCl_3$, 25 °C): δ = 29.5. 40.1, 53.0, 55.9 (2), 64.4, 67.1, 72.3, 78.1, 110.4, 111.2, 121.0, 123.6, 123.9, 126.2, 126.9, 128.2, 128.7, 139.2, 147.8, 149.4 (2), 160.3, 162.4, 166.2; **HRMS (ESI$^+$)**: *m/z* calcd. for $C_{29}H_{30}N_3O_4^+$ $[M + H]^+$ 484.2230, found 484.2252.

^{1}H NMR **6f**

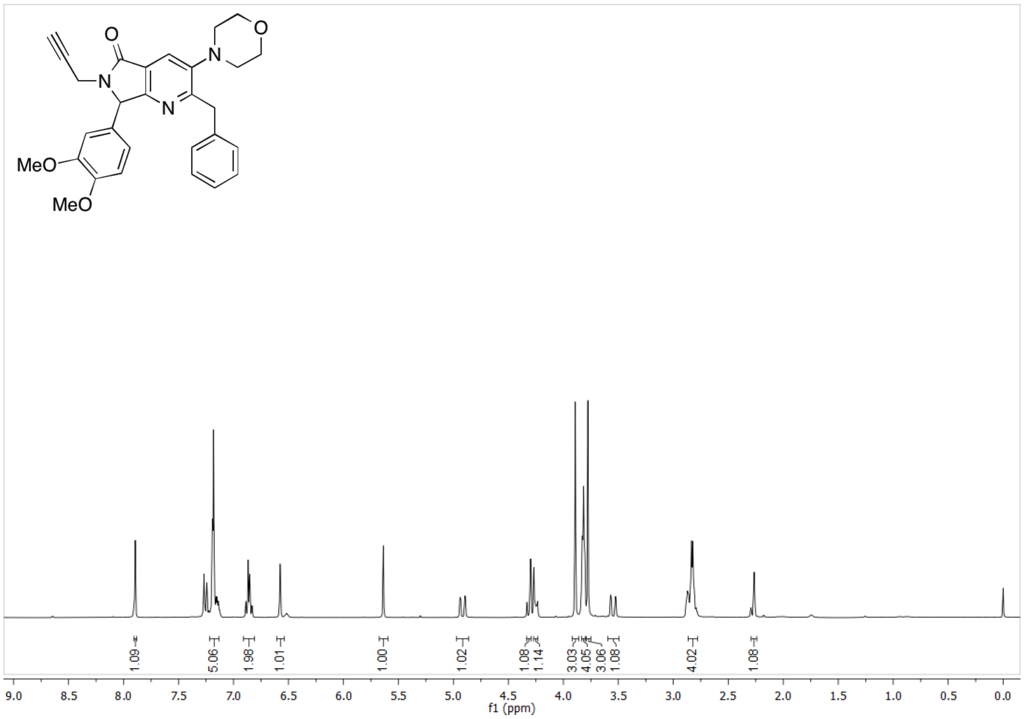

^{13}C NMR **6f**

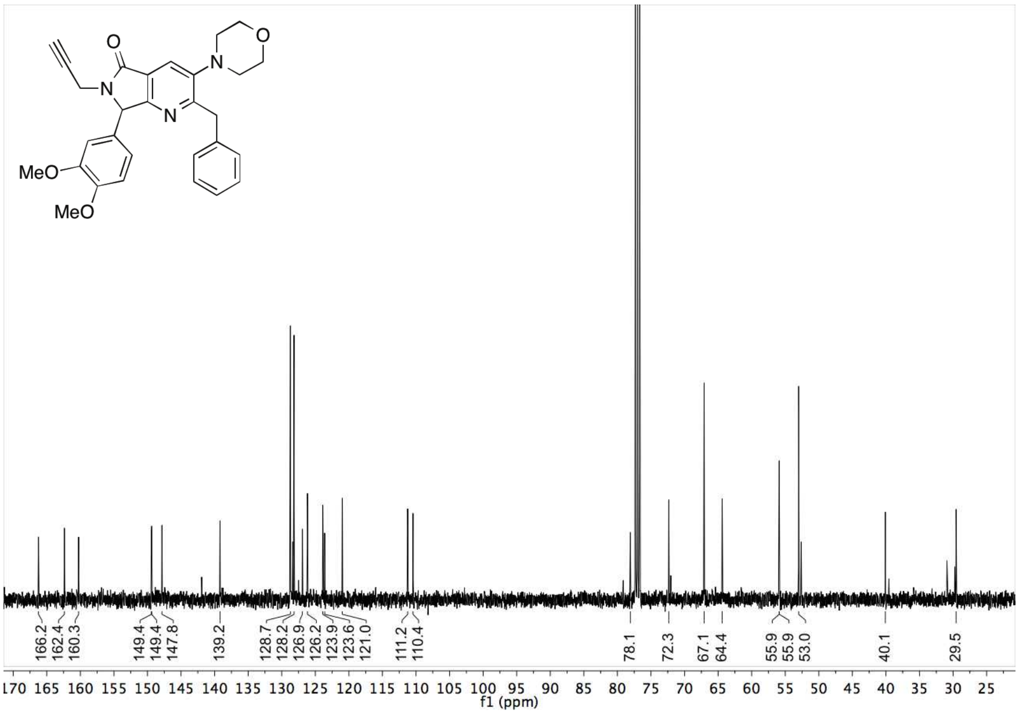

HRMS **6f**

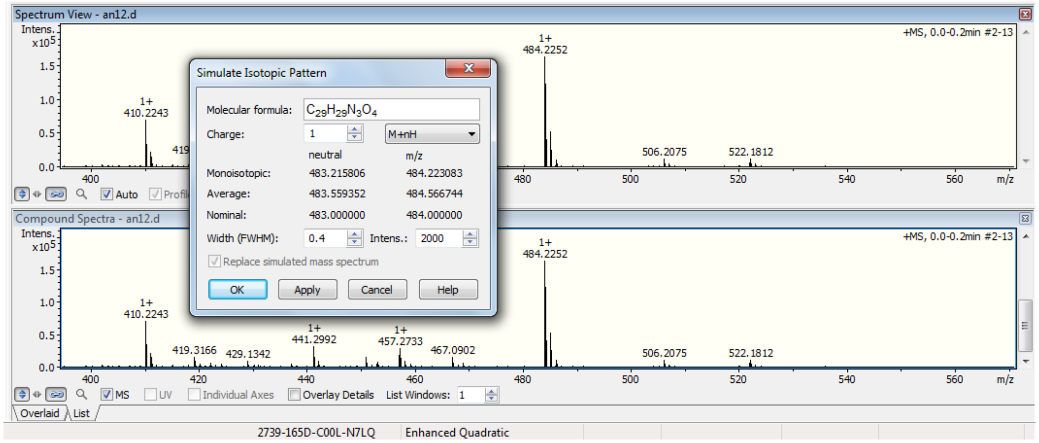

2-Benzyl-7-(4-fluorophenyl)-3-morpholino-6-(prop-2-yn-1-yl)-6,7-dihydro-5*H*-pyrrolo[3,4-*b*]pyridin-5-one (6g): According to GP-1, propargylamine (47.0 μL, 0.726 mmol),4-fluorobenzaldehyde (78.0 μL, 0.726 mmol), scandium triflate (11.0 mg, 0.021 mmol), 2-isocyano-1-morpholino-3-phenylpropan-1-one (213.0 mg, 0.871 mmol), and maleic anhydride (99.0 mg, 1.02 mmol) were reacted together in dry toluene (1.0 mL) to afford the pyrrolo[3,4-*b*]pyridin-5-one **6g**. Yield 66% **(**211.0 mg); yellow oil; $\boldsymbol{R_f}$ = 0.54 (hexanes/EtOAc, 3:2); **FT-IR (ATR)** υ_{max}/cm^{-1} 2119 (C≡C), 1696 (C=O); **^{1}H NMR** (400 MHz; $CDCl_3$, 25 °C): δ = 2.27 (t, *J* = 2.66 Hz, 1H), 2.77–2.88 (m, 4H), 3.52 (dd, *J* = 17.7, 2.5, Hz, 1H), 3.79–3.84 (m, 4H), 4.23 (d, *J* = 13.9 Hz, 1H), 4.30 (d, *J* = 13.9 Hz, 1H), 4.93 (dd, *J* = 17.6, 2.5 Hz, 1H), 5.68 (s, 1H), 7.04–7.09 (m, 2H), 7.13–7.21 (m, 7H), 7.89 (s, 1H); **^{13}C NMR** (101 MHz, $CDCl_3$, 25 °C): δ = 29.5, 40.0, 53.0, 63.7, 67.1, 72.6, 77.8, 115.9, 116.1, 123.5, 123.9, 126.2, 128.2, 128.7, 129.8, 129.9, 130.6 (2), 139.0, 148.0, 160.0, 161.7, 162.5, 164.1, 166.2; **HRMS (ESI$^+$):** *m/z* calcd. for $C_{27}H_{25}FN_3O_2^+$ [M + H]$^+$ 442.1952, found 442.1952.

^{1}H NMR **6g**

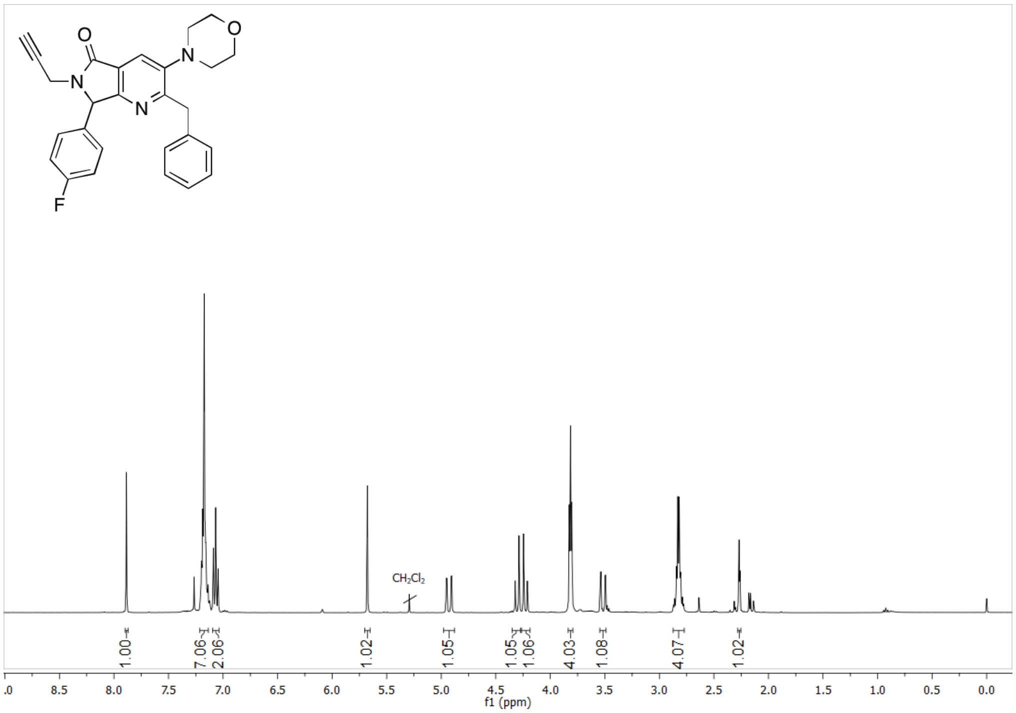

^{13}C NMR **6g**

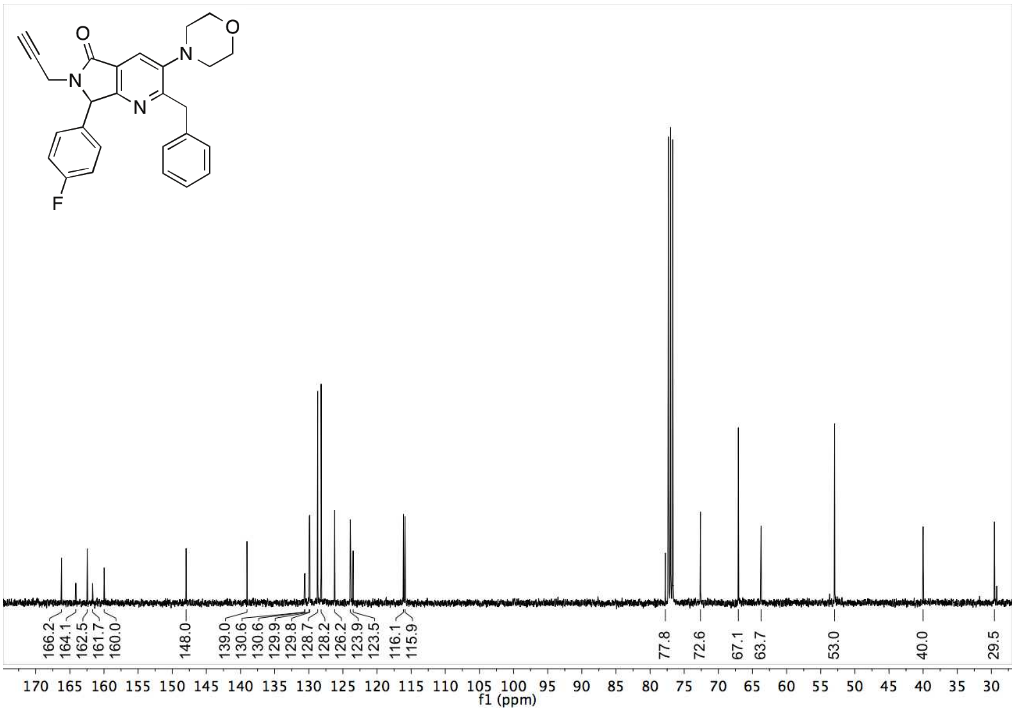

HRMS **6g**

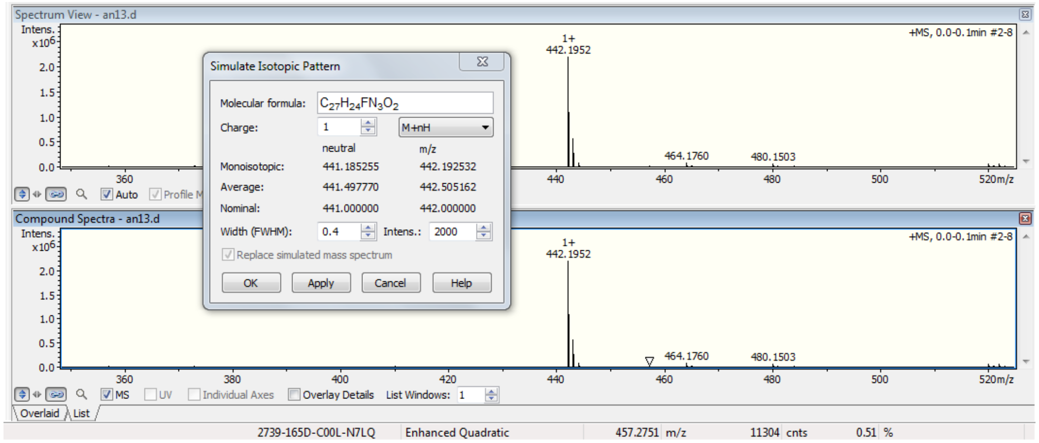

2-Benzyl-7-hexyl-3-morpholino-6-(prop-2-yn-1-yl)-6,7-dihydro-5*H*-pyrrolo[3,4-*b*]pyridin-5-one (6h): According to GP-1, propargylamine (47.0 μL, 0.726 mmol), heptanaldehyde (102.0 μL, 0.726 mmol), scandium triflate (11.0 mg, 0.021 mmol), 2-isocyano-1-morpholino-3-phenylpropan-1-one (213.0 mg, 0.871 mmol), and maleic anhydride (99.0 mg, 1.02 mmol) were reacted together in dry toluene (1.0 mL) to afford the pyrrolo[3,4-*b*]pyridin-5-one **6h**. Yield 67% (209.0 mg); yellow oil; $\boldsymbol{R_f}$ = 0.32 (hexanes/EtOAc, 4:1); **FT-IR (ATR)** υ_{max}/cm^{-1} 2118 (C≡C), 1694 (C=O); **^{1}H NMR** (400 MHz, $CDCl_3$, 25 °C): δ = 0.80–0.88 (m, 4H), 1.11–1.24 (m, 7H) 1.91–2.01 (m, 1H), 2.16–2.25 (m, 1H), 2.27 (s, 1H), 2.78–2.88 (m, 4H), 3.79–3.86 (m, 4H), 3.95 (d, *J* = 17.7 Hz, 1H), 4.29 (d, *J* = 14.0 Hz, 1H), 4.43 (d, *J* = 14.0 Hz, 1H), 4.72– 4.72 (m, 1H), 4.87 (d, *J* = 17.7, 1H), 7.16–7.29 (m, 5H), 7.84 (s, 1H); **^{13}C NMR** (101 MHz, $CDCl_3$, 25 °C): δ = 13.9, 22.4, 22.52, 29.0, 29.2, 29.6, 31.5, 39.8, 52.9, 60.2, 67.0, 72.2, 77.8, 123.6, 124.2, 126.1, 128.2, 128.7, 139.3, 147.4, 160.1, 161.5, 166.4; **HRMS (ESI$^+$):** *m/z* calcd. for $C_{27}H_{34}N_3O_2^+$ [M + H]$^+$ 432.2645, found 432.2704.

^{1}H NMR **6h**

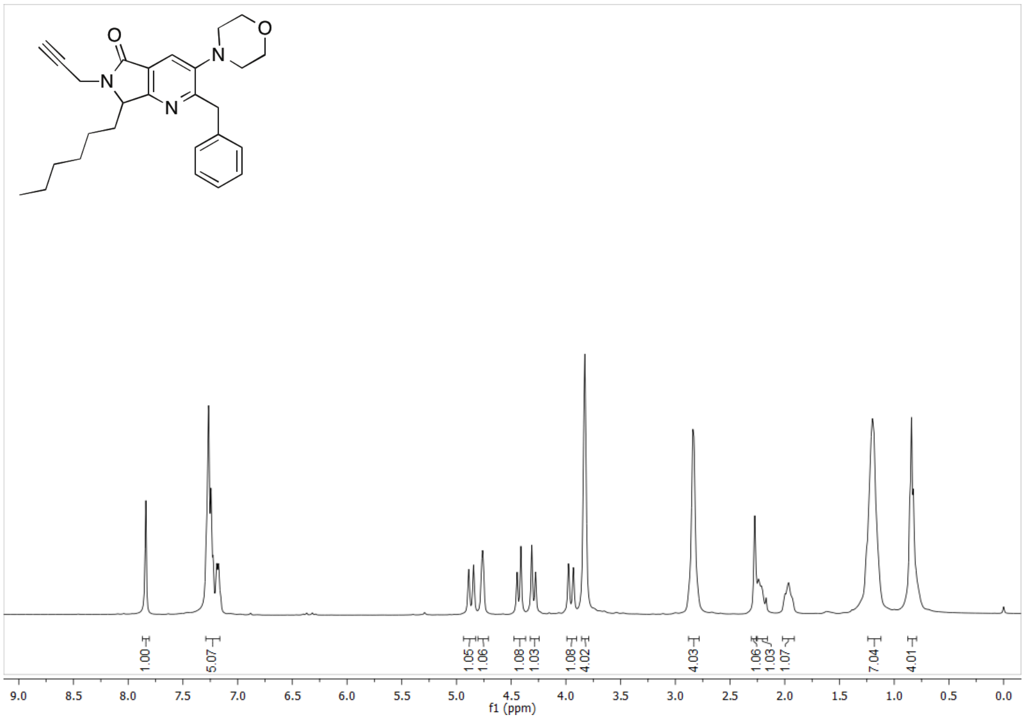

^{13}C NMR **6h**

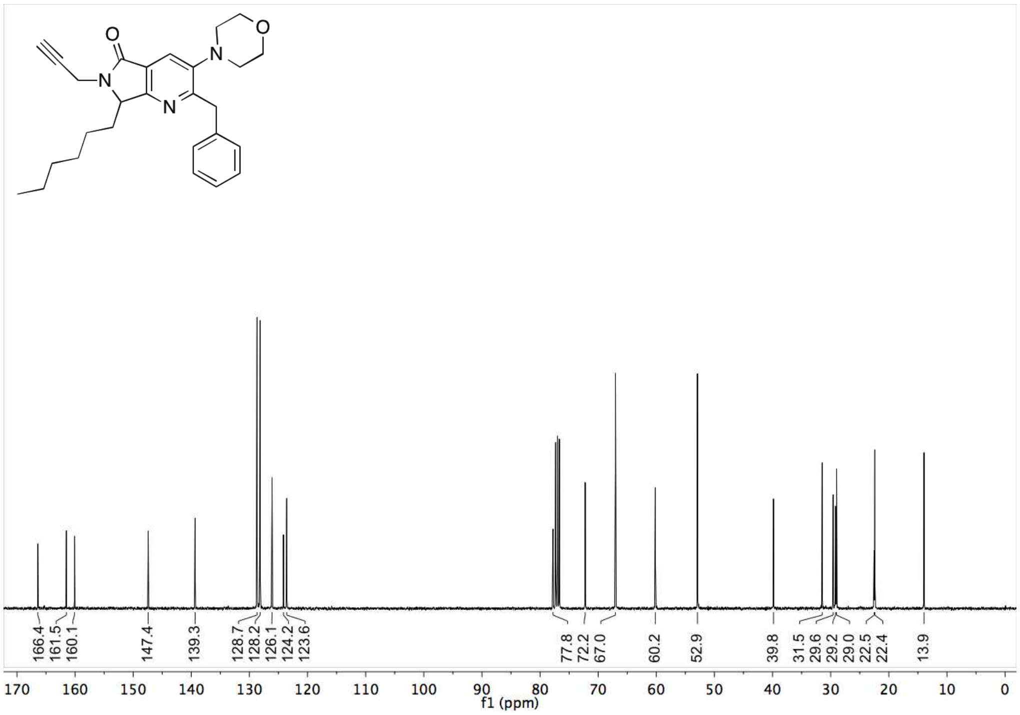

HRMS **6h**

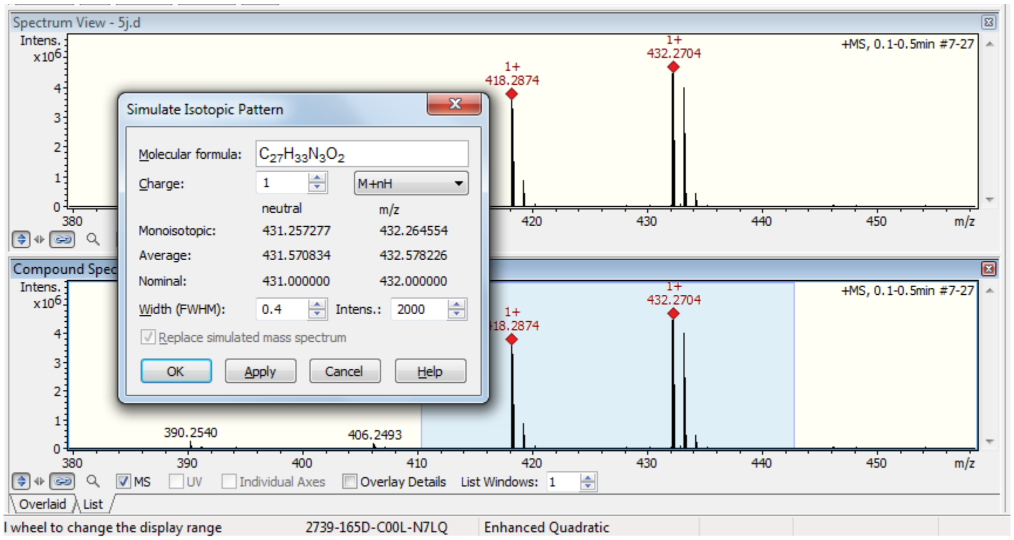

2-Benzyl-3-(diethylamino)-7-phenyl-6-(prop-2-yn-1-yl)-6,7-dihydro-5*H*-pyrrolo[3,4-*b*]pyridin-5-one (6i): According to GP-1, propargylamine (47.0 μL, 0.726 mmol), benzaldehyde (74.0 μL, 0.726 mmol), scandium triflate (11.0 mg, 0.021 mmol), *N*,*N*-diethyl-2-isocyano-3-phenylpropanamide (200.0 mg, 0.871 mmol), and maleic anhydride (99.0 mg, 1.02 mmol) were reacted together in dry toluene (1.0 mL) to afford the pyrrolo[3,4-*b*]pyridin-5-one **6i**. Yield 59% (175.0 mg); yellow gum; $\boldsymbol{R_f}$ = 0.26 (hexanes/EtOAc, 4:1); **FT-IR (ATR)** υ_{max}/cm^{-1} 2119 (C≡C), 1698 (C=O); **^{1}H NMR** (500 MHz, $CDCl_3$, 25 °C): δ = 0.90 (t, *J* = 7.1 Hz, 6H), 1.64–1.77 (m, 4H), 2.26 (t, *J* = 2.5 Hz, 1H), 2.96 (q, *J* = 7.1 Hz, 4H), 3.53 (dd, *J* = 17.6, 2.5 Hz, 1H), 4.20 (d, *J* = 13.8 Hz 1H), 4.30 (d, *J* = 13.8 Hz, 1H), 4.94 (dd, *J* = 17.6, 2.6 Hz, 1H), 5.69 (s, 1H), 7.09–7.16 (m, 5H), 7.18–7.21 (m, 2H), 7.33–7.38 (m, 3H), 7.87 (s, 1H); **^{13}C NMR** (101 MHz, $CDCl_3$, 25 °C): δ = 11.9, 29.5, 39.6, 47.6, 64.4, 72.5, 77.8, 123.2, 125.9, 126.0, 127.9, 128.1, 128.3, 128.8, 128.9, 130.0, 134.7, 139.2, 146.6, 159.4, 163.7, 166.6, 170.4; **HRMS (ESI$^+$):** *m/z* calcd. for $C_{27}H_{28}N_3O^+$ [M + H]$^+$ 410.2226, found 410.2265.

^{1}H NMR **6i**

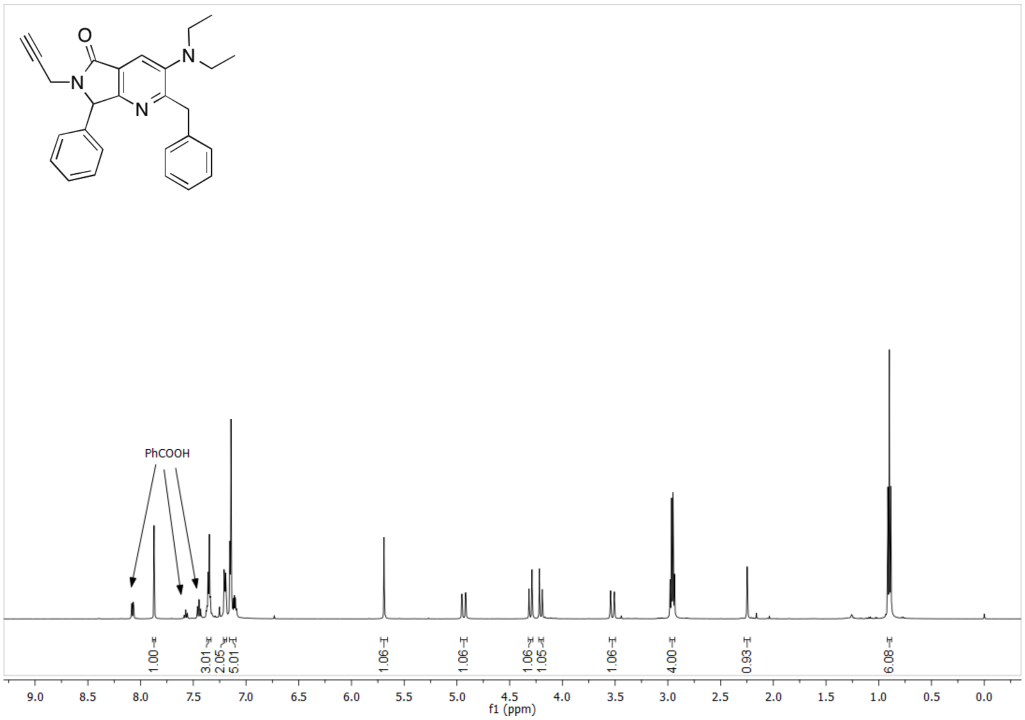

^{13}C NMR **6i**

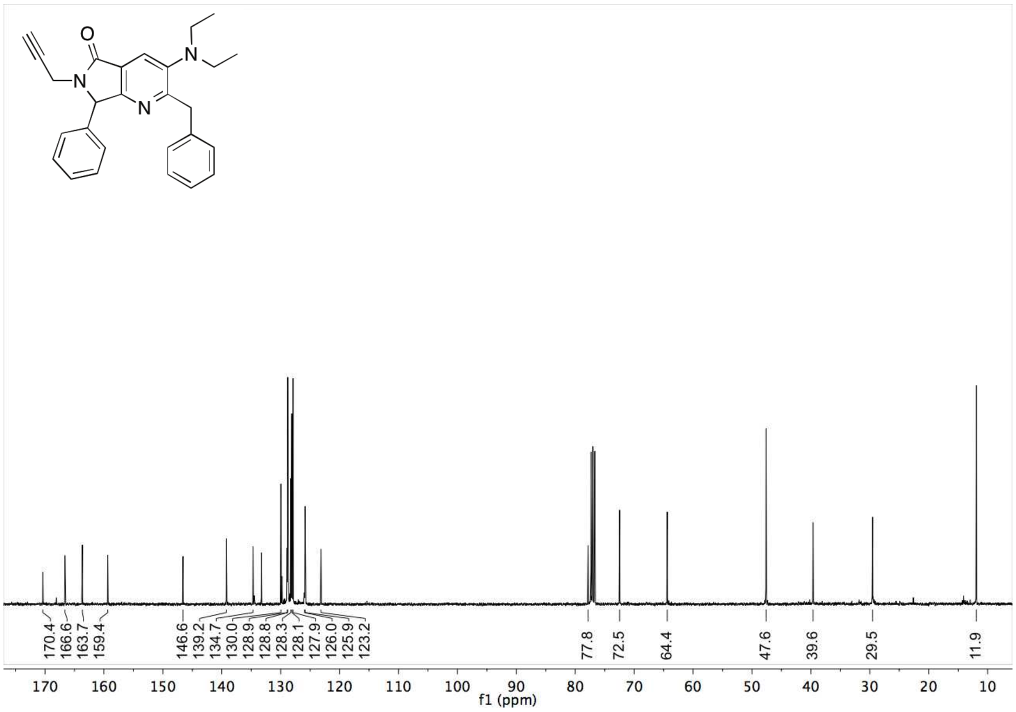

HRMS **6i**

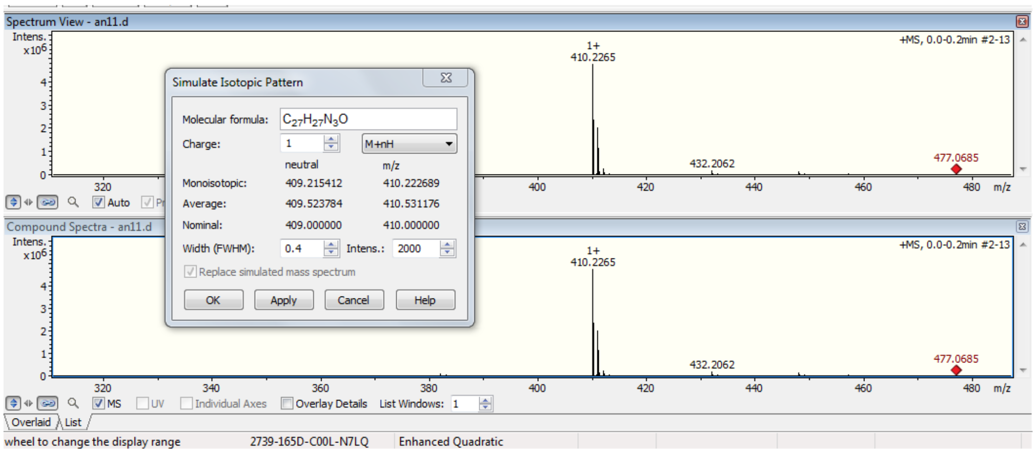

2-Benzyl-3-(diethylamino)-7-(3,4-dimethoxyphenyl)-6-(prop-2-yn-1-yl)-6,7-dihydro-5*H*-pyrrolo[3,4-*b*]pyridin-5-one (6j): According to GP-1, propargylamine (47.0 µL, 0.726 mmol), 3,4-dimethoxybenzaldehyde (120.0 mg, 0.726 mmol), scandium triflate (11.0 mg, 0.021 mmol), *N*,*N*-diethyl-2-isocyano-3-phenylpropanamide (200.0 mg, 0.871 mmol), and maleic anhydride (99.0 mg, 1.02 mmol) were reacted together in dry toluene (1.0 mL) to afford the pyrrolo[3,4-*b*]pyridin-5-one **6j**. Yield 63% (214.0 mg); yellow gum; $\boldsymbol{R_f}$ = 0.28 (hexanes/EtOAc, 4:1); **FT-IR (ATR)** υ_{max}/cm^{-1} 2118 (C≡C), 1696 (C=O); **^{1}H NMR** (500 MHz, $CDCl_3$, 25 °C): δ = 0.90 (t, *J* = 7.0 Hz, 6H), 2.36 (s, 1H), 2.97 (q, *J* = 7.0 Hz, 4H), 3.55 (dd, *J* = 17.8, 2.4 Hz, 1H), 3.77 (s, 3H), 3.89 (s, 3H), 4.23 (d, *J* = 13.8 Hz, 1H), 4.30 (d, *J* = 13.8 Hz, 1H), 4.91 (d, *J* = 17.6, 2.5 Hz, 1H), 5.62 (s, 1H), 6.57 (s, 1H), 6.81–6.90 (m, 2H), 7.11–7.19 (m, 5H), 7.86 (s, 1H); **^{13}C NMR** (126 MHz, $CDCl_3$, 25 °C): δ = 12.0. 29.5, 39.9, 47.7, 55.9, 64.4, 72.3, 78.2, 110.5, 111.2, 120.9, 123.1, 125.7, 125.9, 127.2, 127.9, 128.9, 139.5, 146.5, 149.3, 159.6, 163.9,166.6; **HRMS (ESI$^+$):** *m/z* calcd. for $C_{29}H_{32}N_3O_3^+$ [M + H]$^+$ 470.2438, found 470.2546.

^{1}H NMR **6j**

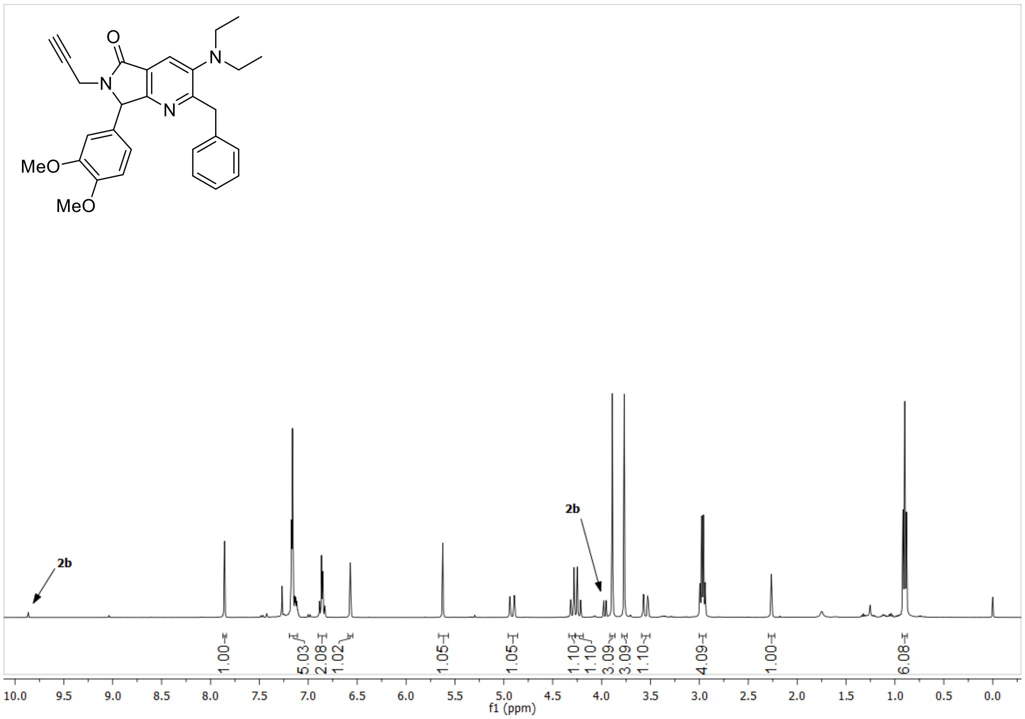

^{13}C NMR **6j**

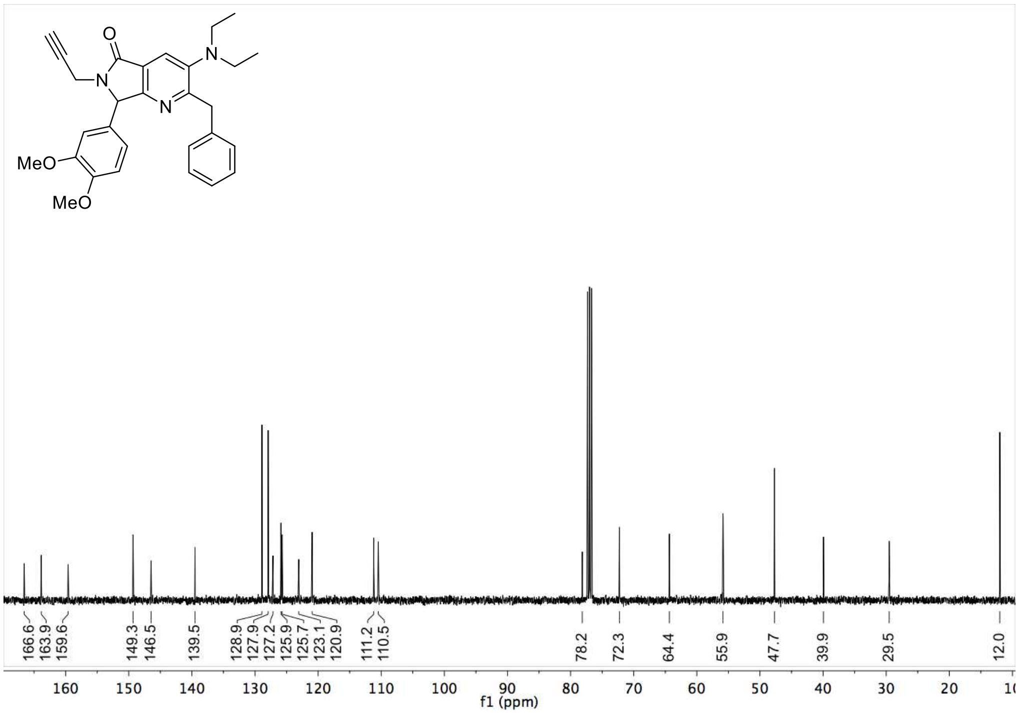

HRMS **6j**

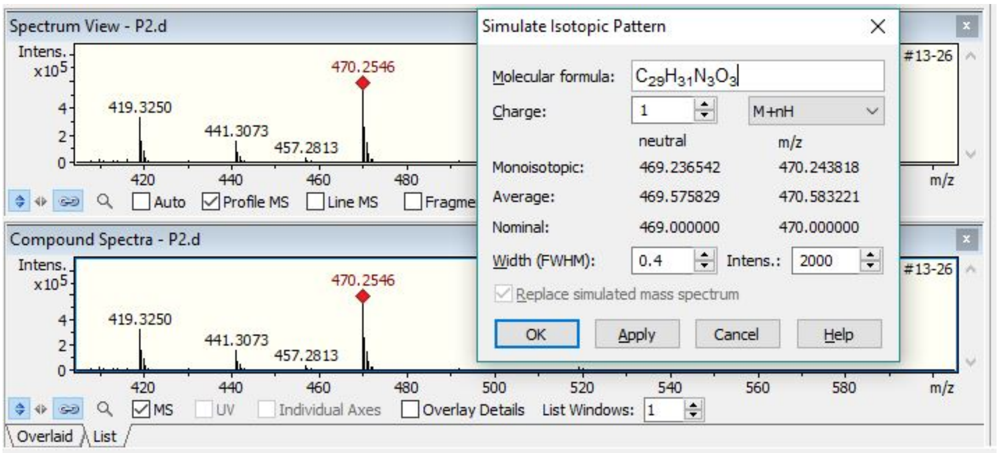

2-Benzyl-3-(diethylamino)-7-(4-fluorophenyl)-6-(prop-2-yn-1-yl)-6,7-dihydro-5*H*-pyrrolo[3,4-*b*]pyridin-5-one (6k): According to GP-1, propargylamine (47.0 µL, 0.726 mmol), 4-flourobenzaldehyde (78.0 µL, 0.726 mmol), scandium triflate (11.0 mg, 0.021 mmol), *N*,*N*-diethyl-2-isocyano-3-phenylpropanamide (200.0 mg, 0.871 mmol), and maleic anhydride (99.0 mg, 1.02 mmol) were reacted together in dry toluene (1.0 mL) to afford the pyrrolo[3,4-*b*]pyridin-5-one **6k**. Yield 64% (198.0 mg); yellow gum; $\boldsymbol{R_f}$ = 0.42 (hexanes/EtOAc, 4:1); **FT-IR (ATR)** υ_{max}/cm^{-1} 2118 (C≡C), 1698 (C=O); **^{1}H NMR** (500 MHz, $CDCl_3$, 25 °C): δ = 0.91 (t, *J* = 7.1, 6H), 2.26 (s, 1H), 2.97 (q, *J* =7.1, 4H), 3.52 (d, *J* = 17.6, 2.4 Hz, 1H), 4.21 (d, *J* = 13.9 Hz, 1H), 4.29 (d, *J* = 13.9 Hz, 1H), 4.92 (d, *J* = 17.6, 2.5 Hz, 1H), 5.67 (s, 1H), 7.03–7.20 (m, 5H), 7.86 (s, 1H); **^{13}C NMR** (126 MHz; $CDCl_3$, 25 °C): δ = 12.0, 29.5, 39.8, 47.6, 63.7, 72.5, 77.8, 115.8, 116.0, 123.0, 125.8, 125.9, 127.9, 128.9, 129.9 (2), 130.8 (2), 139.3, 146.6, 159.3, 161.6, 163.9, 164.1, 166.6; **HRMS (ESI$^+$):** *m/z* calcd. for $C_{27}H_{27}FN_3O^+$ [M + H]$^+$ 428.2132, found 428.2210.

^{1}H NMR **6k**

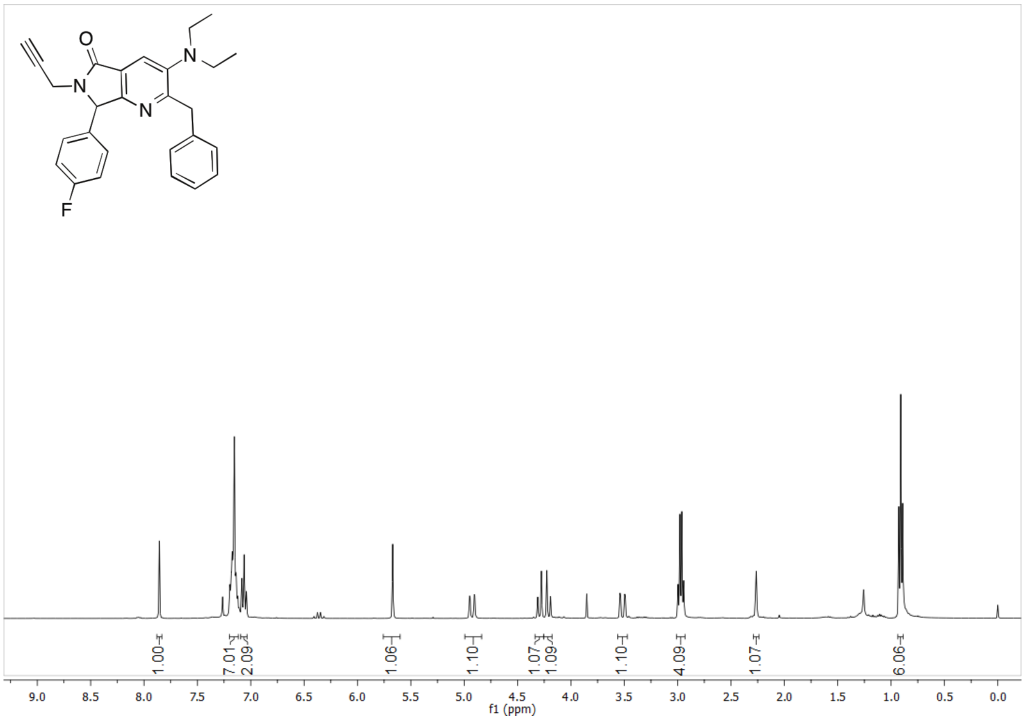

^{13}C NMR **6k**

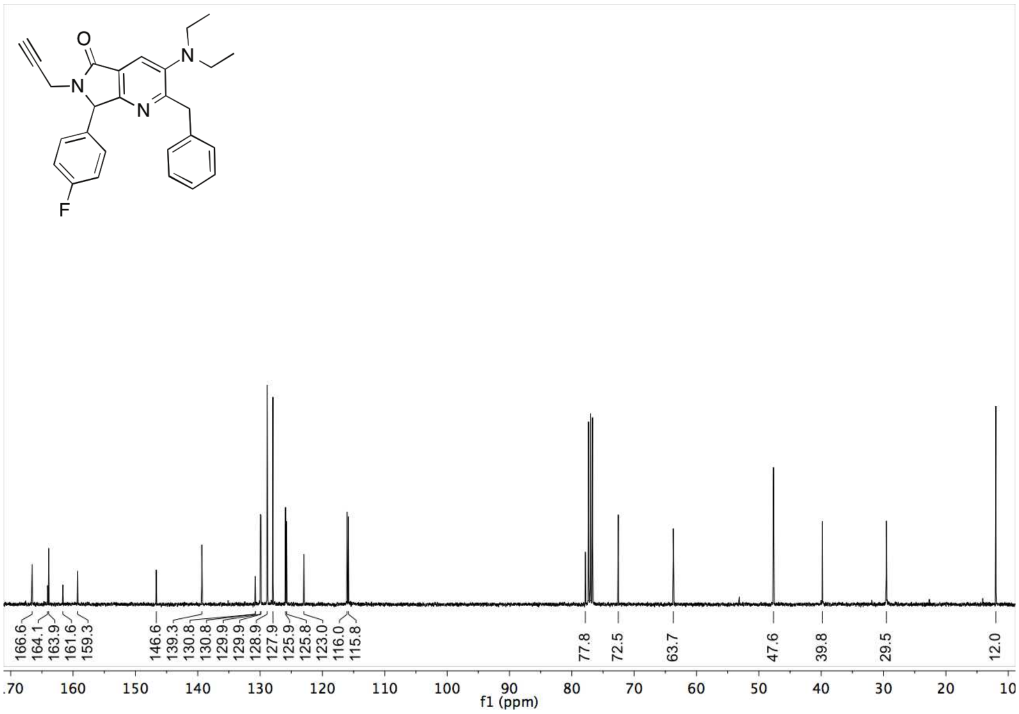

HRMS **6k**

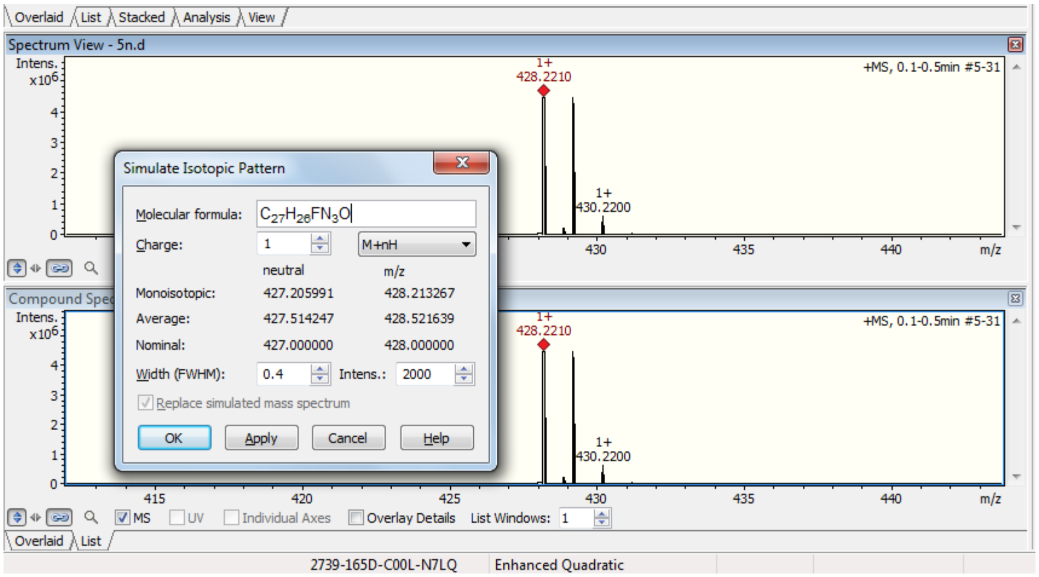

2-Benzyl-3-(diethylamino)-7-hexyl-6-(prop-2-yn-1-yl)-6,7-dihydro-5*H*-pyrrolo[3,4-*b*]pyridin-5-one (6l): According to GP-1, propargylamine (47.0 μL, 0.726 mmol), 4-flourobenzaldehyde (102.0 μL, 0.726 mmol), scandium triflate (11.0 mg, 0.021 mmol), *N*,*N*-diethyl-2-isocyano-3-phenylpropanamide (200.0 mg, 0.871 mmol), and maleic anhydride (99.0 mg, 1.02 mmol) were reacted together in dry toluene (1.0 mL) to afford the pyrrolo[3,4-*b*]pyridin-5-one **6l**. Yield 66% (199.0 mg); yellow gum; ***R*** R_f = 0.64 (hexanes/EtOAc, 7:3); **FT-IR (ATR)** υ_{max}/cm^{-1} 2118 (C≡C), 1698 (C=O); **^{1}H NMR** (500 MHz, $CDCl_3$, 25 °C): δ = 0.81–0.87 (m, 4H), 0.91 (t, *J* = 7.0 6H), 1.14–1.23 (m, 7H), 1.89–1.98 (m, 1H), 2.14–2.23 (m, 1H), 2.26 (s, 1H), 2.97 (q, *J* = 7.0 Hz, 4H), 3.95 (dd, *J* = 17.8, 2.4 Hz, 1H), 4.25 (d, *J* = 14.0 Hz, 1H), 4.44 (d, *J* = 14.0 Hz, 1H), 4.69–4.78 (m, 1H), 4.86 (dd, *J* = 17.8, 2.5 Hz, 1H), 7.18–7.27 (m, 5H), 7.81 (s, 1H); **^{13}C NMR** (126 MHz, $CDCl_3$, 25 °C): δ = 12.1, 14.0, 22.5, 22.7, 29.1, 29.3, 29.6, 31.6, 39.7, 47.9, 60.2, 72.1, 78.0, 123.7, 125.6, 125.9, 128.0, 129.0, 139.8, 146.0, 159.7, 163.2, 166.8; **HRMS (ESI$^+$):** *m/z* calcd. for $C_{27}H_{36}N_3O^+$ [M + H]$^+$ 418.2852, found 418.2921.

^{1}H NMR **6l**

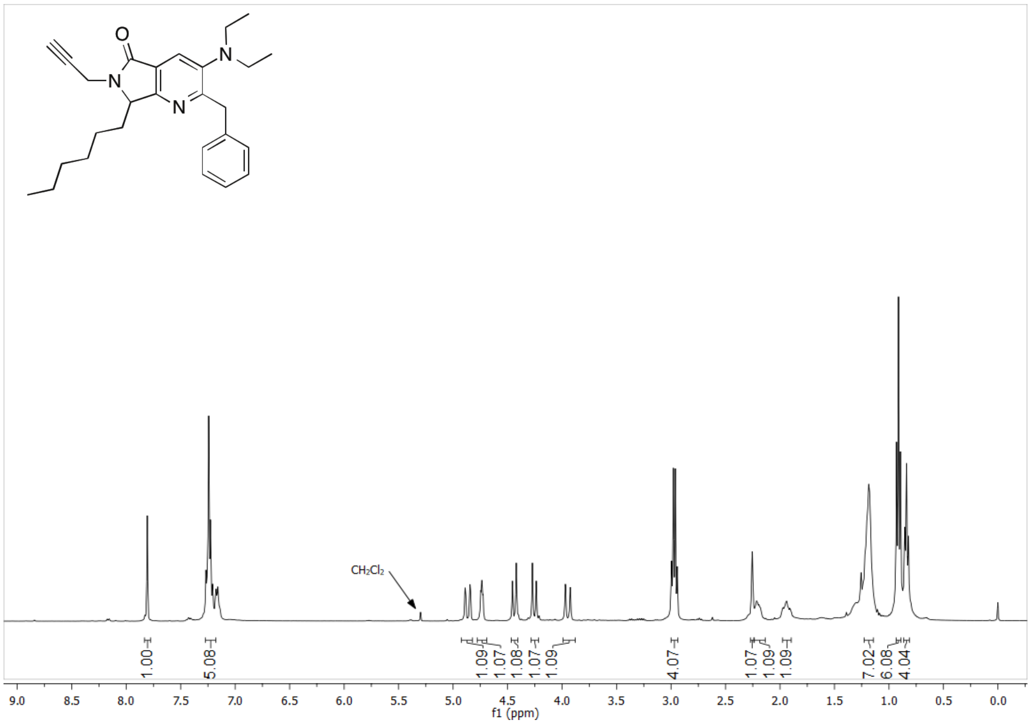

^{13}C NMR **6l**

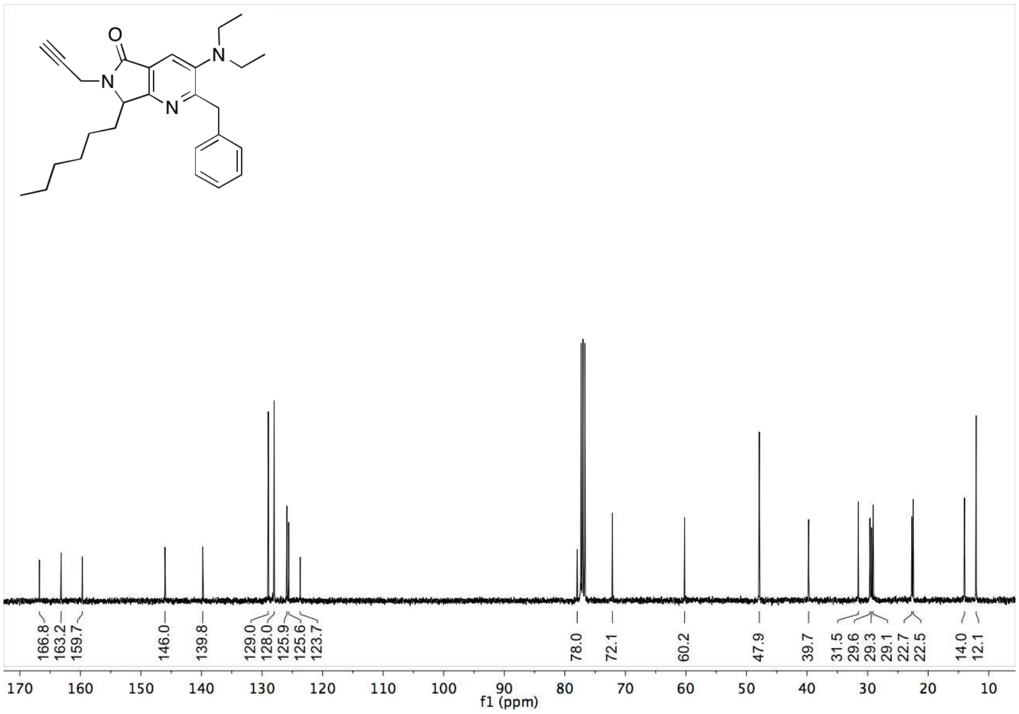

HRMS **6l**

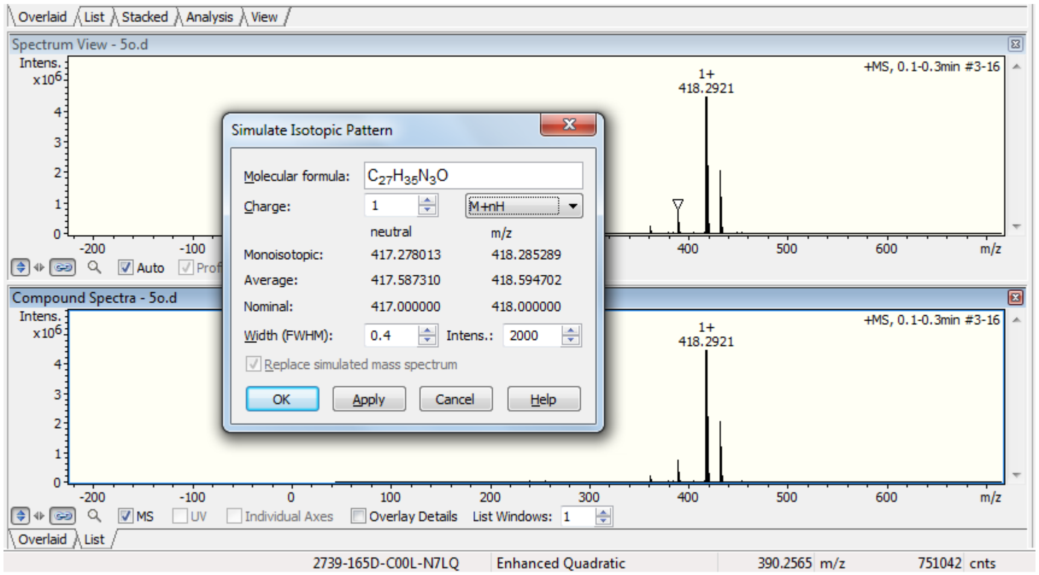

2-Benzyl-7-(4-chlorophenyl)-3-morpholino-6-(prop-2-yn-1-yl)-6,7-dihydro-5*H*-pyrrolo[3,4-*b*]pyridin-5-one (6m): According to GP-1, propargylamine (23.0 μL, 0.355 mmol),4-chlorobenzaldehyde (50.0 mg, 0.355 mmol), scandium triflate (8.0 mg, 0.017 mmol), 2-isocyano-1-morpholino-3-phenylpropan-1-one (104.0 mg, 0.426 mmol), and maleic anhydride (49.0 mg, 0.498 mmol) were reacted together in dry toluene (1.0 mL) to afford the pyrrolo[3,4-*b*]pyridin-5-one **6m**. Yield 60% (98.0 mg); white solid; **m.p.** 69-71 °C ***R**$_f$* = 0.54 (hexanes/EtOAc, 3:2); **FT-IR (ATR)** υ_{max}/cm^{-1} 2117 (C≡C), 1695 (C=O); **^{1}H NMR** (400 MHz; CDCl$_3$, 25 °C): δ = 2.28 (t, *J* = 2.5 Hz, 1H), 2.78–2.95 (m, 4H), 3.52 (dd, *J* = 17.7, 2.5, Hz, 1H), 3.76–3.86 (m, 4H), 4.24 (d, *J* = 13.9 Hz, 1H), 4.33 (d, *J* = 13.8 Hz, 1H), 4.95 (dd, *J* = 17.6, 2.6 Hz, 1H), 5.68 (s, 1H), 7.14–7.23 (m, 7H), 7.35–7.39 (m, 2H), 7.90 (s, 1H); **^{13}C NMR** (101 MHz, CDCl$_3$, 25 °C): δ = 29.7, 40.1, 53.0, 63.8, 67.1, 72.7, 77.8, 123.5, 124.0, 126.2, 128.2, 128.7, 129.2, 129.4, 133.6, 134.7, 139.1, 148.1, 159.8, 162.6, 166.3; **HRMS (ESI$^+$):** *m/z* calcd. for $C_{27}H_{25}ClN_3O_2^+$ [M + H]$^+$ 458.1629, found 458.1642.

^{1}H NMR **6m**

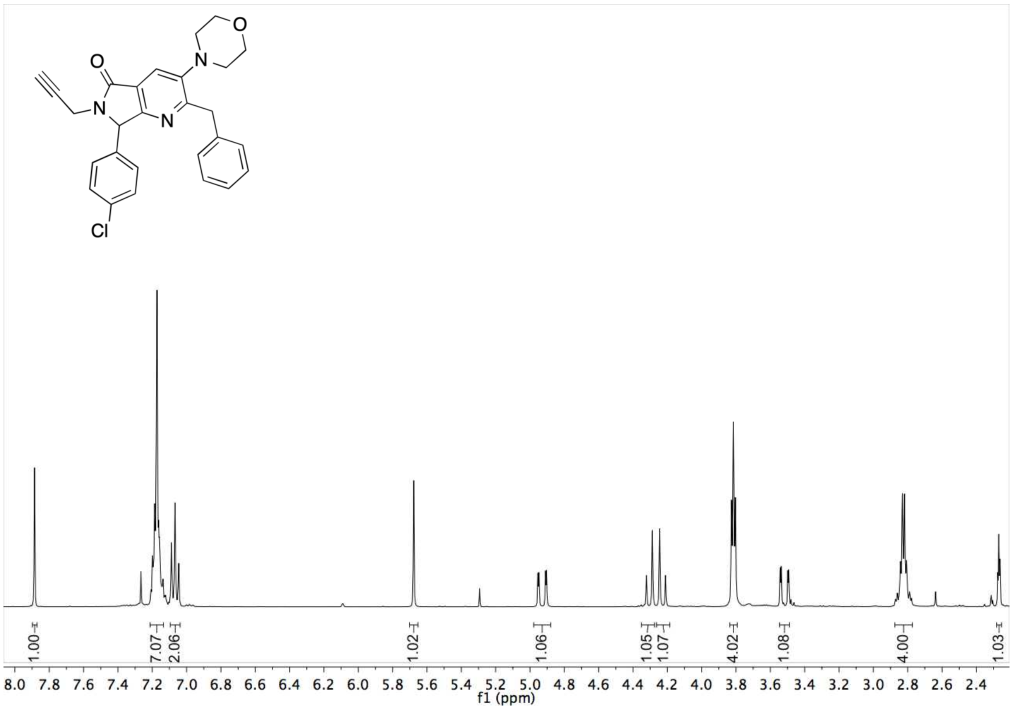

^{13}C NMR **6m**

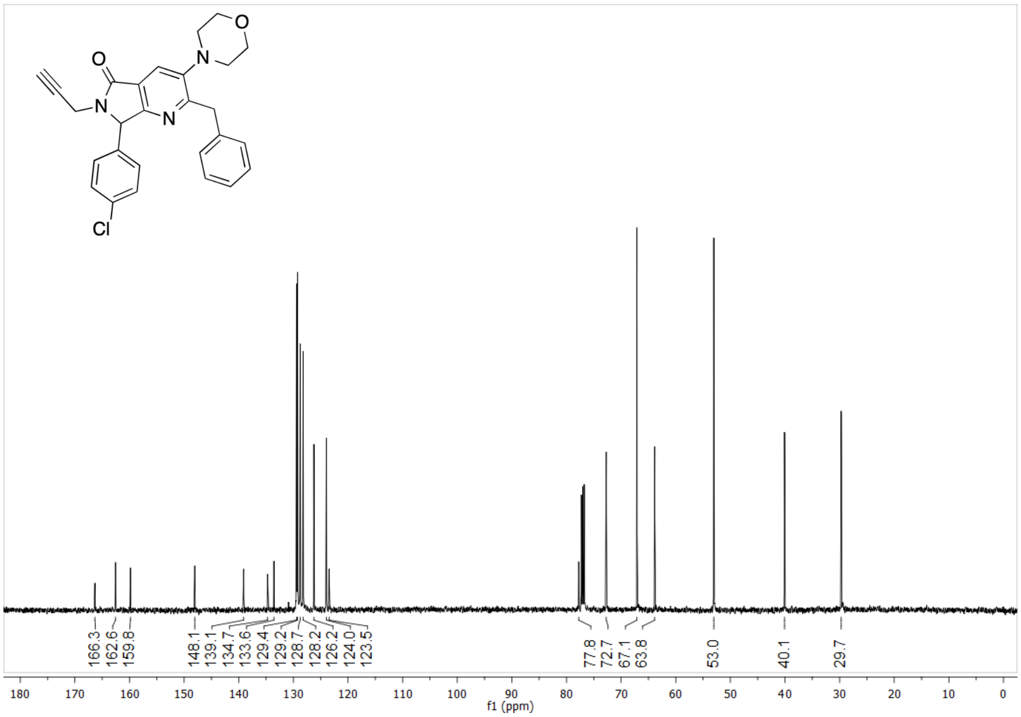

HRMS **6m**

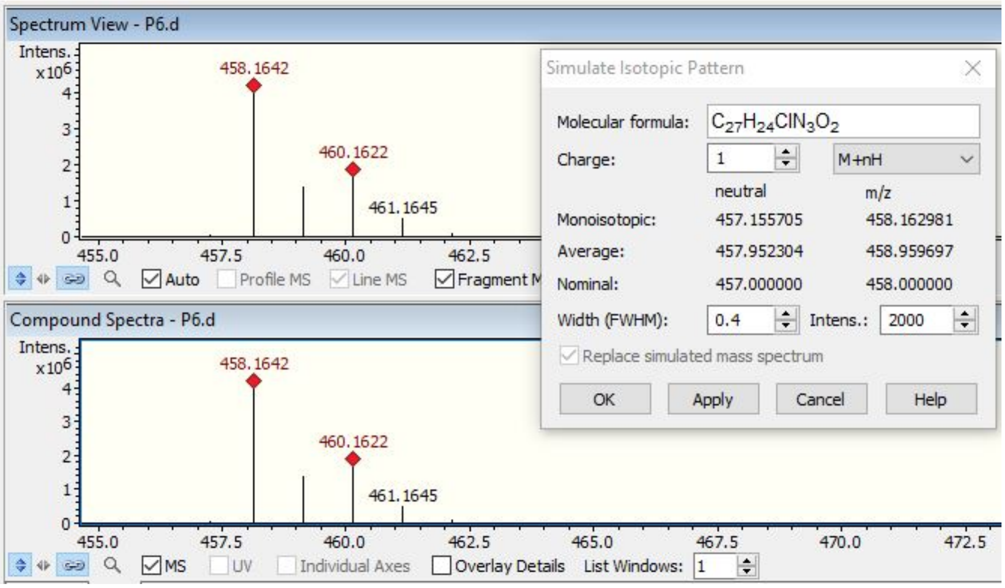

General procedure for the synthesis and characterization of 6-((1*H*-1,2,3-triazol-4-yl)methyl)-pyrrolo[3,4-b]pyridin-5-ones 9a-l (GP-2): To a 0.5 M solution of the corresponding pyrrolo[3,4-*b*]pyridin-5-one **6** (1.0 equiv.) in DMF/MeOH (9:1 v/v) under nitrogen atmosphere in a round-bottomed flask equipped with a magnetic stirring bar were added $TMSN_3$ (**8**)(2.0 equiv.) and CuI (3% mol) sequentially. The reaction mixture was stirred at 100 °C and monitored by TLC. Once the starting material disappeared, the reaction mixture was diluted in water (5.0 mL) and extracted with dichloromethane (2 x 10 mL). The organic layer was washed with water (2 x 10 mL) and brine (2 x 10 mL). The new organic layer was dried over anhydrous Na_2SO_4 and concentrated under vacuum to afford the crude product. The residue was purified by flash chromatography to afford the corresponding 6-((1*H*-1,2,3-triazol-4-yl)methyl)-pyrrolo[3,4-*b*]pyridin-5-ones **9a-l.**

6-((1*H*-1,2,3-Triazol-4-yl)methyl)-2-benzyl-7-phenyl-3-(piperidin-1-yl)-6,7-dihydro-5*H*-pyrrolo[3,4-*b*]pyridin-5-one (9a): According to GP-2, pyrrolo[3,4-*b*]pyridin-5-one **6a** (200 mg, 0.474 mmol), $TMSN_3$ (126.0 μL, 0.949 mmol), and CuI (3.0 mg, 0.016 mmol), were reacted together in a mixture 0.5 M of DMF/MeOH (9/1 v/v) to afford the 6-((1*H*-1,2,3-triazol-4-yl)methyl)-pyrrolo[3,4-*b*]pyridin-5-one **9a**. Yield 77% (169.0 mg); orange solid; $\boldsymbol{R_f}$ = 0.30 (hexanes/EtOAc, 1:1); **m.p.** 178-179 °C; **FT-IR (ATR)** υ_{max}/cm^{-1} 1637 (C=O); **^{1}H NMR** (500 MHz, $CDCl_3$, 25 °C): δ = 1.55–1.59 (m, 2H), 1.67–1.71 (m, 4H), 2.74–2.82 (m, 4H), 4.11–4.19 (m, 2H), 4.27 (d, *J* = 13.8 Hz, 1H), 5.27 (d, *J* = 15.5 Hz, 1H), 5.50 (s, 1H), 7.08–7.19 (m, 7H), 7.31–7.35 (m, 3H), 7.55 (s, 1H), 7.86 (s, 1H); **^{13}C NMR** (126 MHz, $CDCl_3$, 25 °C): δ = 23.8, 26.3, 34.8, 39.6, 54.2, 65.0, 123.6 (2), 126.0, 128.0, 128.2, 128.7, 128.9, 134.9, 139.2, 149.6, 159.5, 162.3, 167.4; **HRMS (ESI$^+$):** *m/z* calcd. for $C_{28}H_{29}N_6O^+$ $[M + H]^+$ 465.2397, found 465.2051.

^{1}H NMR **9a**

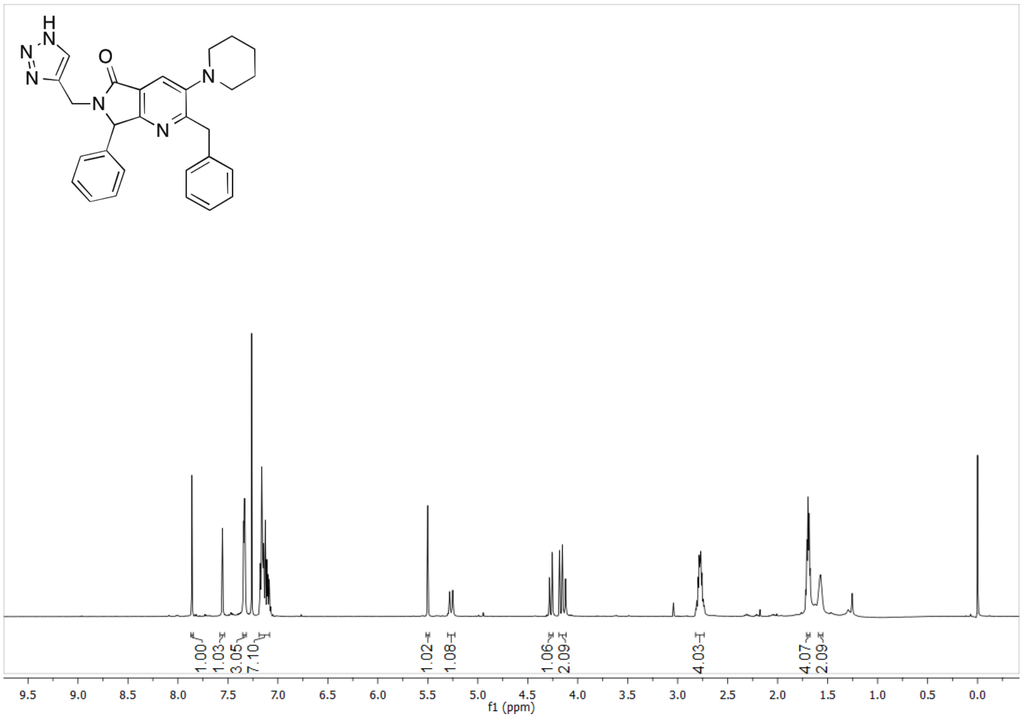

^{13}C NMR **9a**

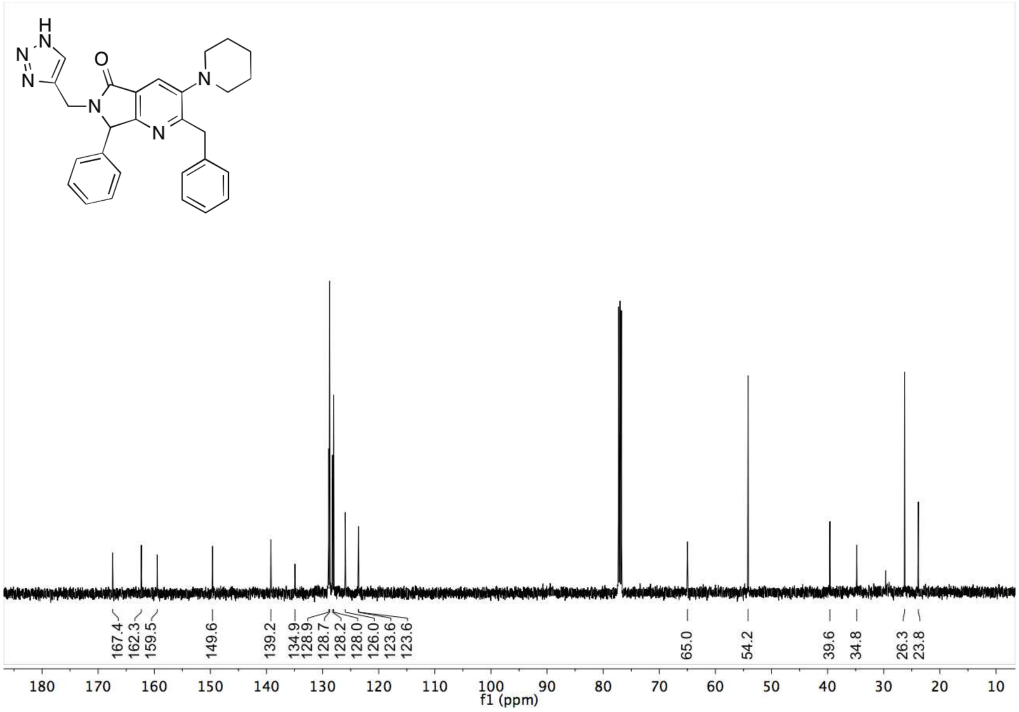

HRMS **9a**

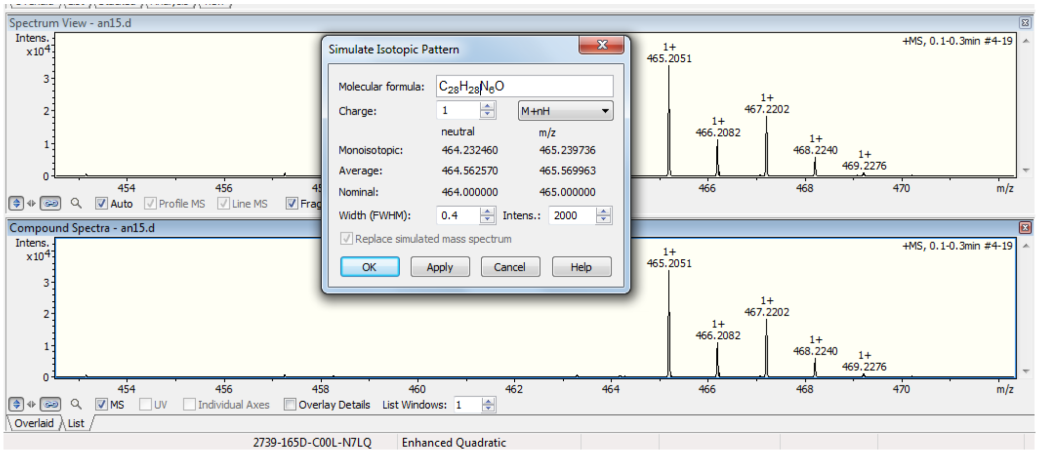

6-((1*H*-1,2,3-Triazol-4-yl)methyl)-2-benzyl-7-(3,4-dimethoxyphenyl)-3-(piperidin-1-yl)-6,7-dihydro-5*H*-pyrrolo[3,4-*b*]pyridin-5-one (9b): According to GP-2, pyrrolo[3,4-*b*]pyridin-5-one **6b** (200.0 mg, 0.415 mmol), $TMSN_3$ (110.0 µL, 0.830 mmol), and CuI (3.0 mg, 0.016 mmol), were reacted together in a mixture 0.5 M of DMF/MeOH (9/1 v/v) to afford the 6-((1*H*-1,2,3-triazol-4-yl)methyl)-pyrrolo[3,4-*b*]pyridin-5-one **9b**. Yield 75% (162.0 mg); yellow solid; $\boldsymbol{R_f}$ = 0.35 (hexanes/EtOAc, 1:1); **m.p.** 199-200 °C; **FT-IR (ATR)** υ_{max}/cm^{-1} 1666 (C=O); **^{1}H NMR** (500 MHz, $CDCl_3$, 25 °C): δ = 1.54–1.60 (m, 2H), 1.67–1.72 (m, 4H), 2.75–2.81 (m, 4H), 3.69 (s, 3H), 3.86 (s, 3H), 4.13 (d, *J* = 15.5 Hz, 1H), 4.22 (d, *J* = 13.9 Hz, 1H), 4.27 (d, *J* = 14.0 Hz, 1H), 5.27 (d, *J* = 15.5 Hz, 1H), 5.49 (s, 1H), 6.55 (s, 1H), 6.78 (d, *J* = 8.2 Hz, 1H), 6.82 (d, *J* = 8.2 Hz, 1H), 7.06–7.19 (m, H), 7.56 (s, 1H), 7.88 (s, 1H); **^{13}C NMR** (126 MHz, $CDCl_3$, 25 °C): δ = 23.8, 26.3, 34.7, 39.7, 54.2, 55.9, 64.9, 110.7, 111.2, 121.1, 123.6, 126.0, 127.1, 128.0, 128.8, 139.3, 149.4, 149.6, 159.5, 162.3, 167.3; **HRMS (ESI$^+$):** *m/z* calcd. for $C_{30}H_{33}N_6O_3^+$ $[M + H]^+$ 525.2609, found 425.2581.

^{1}H NMR **9b**

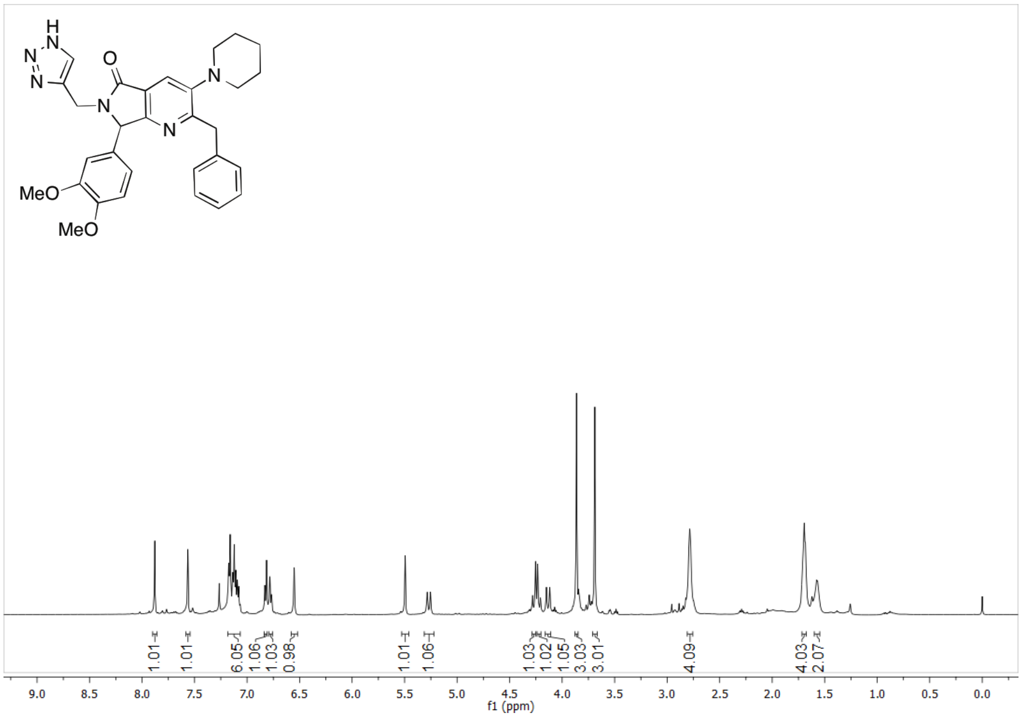

^{13}C NMR **9b**

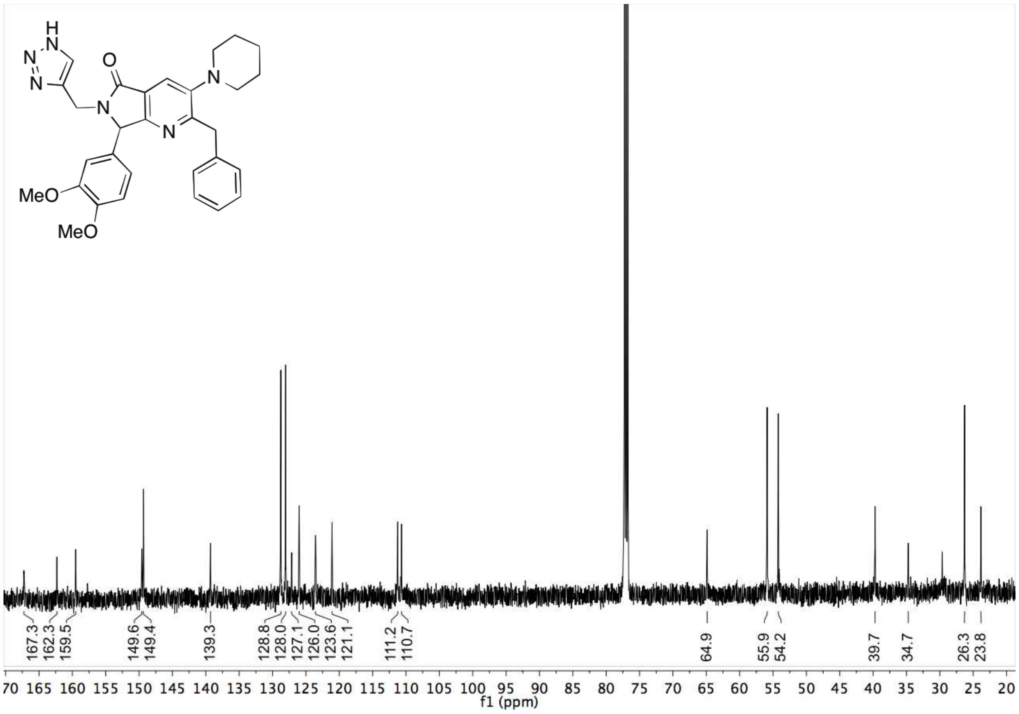

HRMS **9b**

Mass Spectrum SmartFormula Report

Analysis Info

		Acquisition Date	2/28/2017 12:31:55 PM
Analysis Name	D:\Data\Monica Rincon\Eduardo_Gonzalez\20170228_6b_Pos.d		
Method	tune_low_cres_110117MS.m	Operator	CBS_UAM_I
Sample Name	20170228_6b_Pos	Instrument	micrOTOF 213750.00410
Comment	20170228_6b_Pos		

Acquisition Parameter

Source Type	ESI	Ion Polarity	Positive	Set Nebulizer	3.0 Bar
Focus	Active	Set Capillary	4500 V	Set Dry Heater	210 °C
Scan Begin	50 m/z	Set End Plate Offset	-500 V	Set Dry Gas	10.0 l/min
Scan End	3000 m/z	Set Charging Voltage	0 V	Set Divert Valve	Source
		Set Corona	0 nA	Set APCI Heater	0 °C

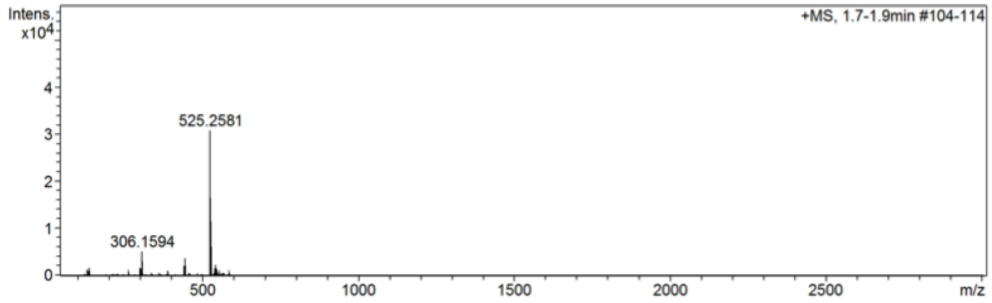

Meas. m/z	#	Ion Formula	m/z	err [ppm]	mSigma	# Sigma	Score	rdb	e⁻ Conf	N-Rule
306.1594	1	C19H20N3O	306.1601	-2.2	17.9	1	100.00	11.5	even	ok
444.2254	1	C23H26N9O	444.2255	0.1	9.5	1	100.00	15.5	even	ok
	2	C22H30N5O5	444.2241	2.9	16.9	2	45.59	10.5	even	ok
525.2581	1	C30H33N6O3	525.2609	-5.2	13.8	2	35.05	17.5	even	ok

6-((1*H*-1,2,3-Triazol-4-yl)methyl)-2-benzyl-7-(4-fluorophenyl)-3-(piperidin-1-yl)-6,7-dihydro-5*H*-pyrrolo[3,4-*b*]pyridin-5-one (9c): According to GP-2, pyrrolo[3,4-*b*]pyridin-5-one **6c** (200.0 mg, 0.455 mmol), $TMSN_3$ (121.0 µL, 0.910 mmol), and CuI (3.0 mg, 0.016 mmol), were reacted together in a mixture 0.5 M of DMF/MeOH (9/1 v/v) to afford the 6-((1*H*-1,2,3-triazol-4-yl)methyl)-pyrrolo[3,4-*b*]pyridin-5-one **9c**. Yield 80% (175.0 mg); yellow solid; $\boldsymbol{R_f}$ = 0.46 (hexanes/EtOAc, 1:1); **m.p.** 187-188 °C; **FT-IR (ATR)** υ_{max}/cm^{-1} 1659 (C=O); **^{1}H NMR** (500 MHz, $CDCl_3$, 25 °C): δ = 1.54–1.60 (m, 4H), 1.67–1.72 (m, 4H), 2.73–2.82 (m, 4H), 4.08 (d, *J* = 15.5 Hz, 1H), 4.19 (d, *J* = 13.9 Hz, 1H), 4.27 (d, *J* = 13.9 Hz, 1H), 5.28 (d, *J* = 15.5 Hz, 1H), 5.52 (s, 1H), 6.95–7.02 (m, 2H), 7.07–7.17 (m, 7H), 7.55 (s, 1H), 7.89 (s, 1H); **^{13}C NMR** (126 MHz; $CDCl_3$, 25 °C): δ = 23.8, 26.3, 34.8, 39.6, 54.2, 64.2, 115.8, 116.0, 123.5, 123.6, 126.0, 128.1, 128.7, 130.0, 130.1, 130.7, 130.8, 139.2, 149.7, 159.2, 161.9, 162.4, 163.8, 167.3; **HRMS (ESI$^+$):** *m/z* calcd. for $C_{28}H_{28}FN_6O^+$ $[M + H]^+$ 483.2303, found 483.2300.

^{1}H NMR **9c**

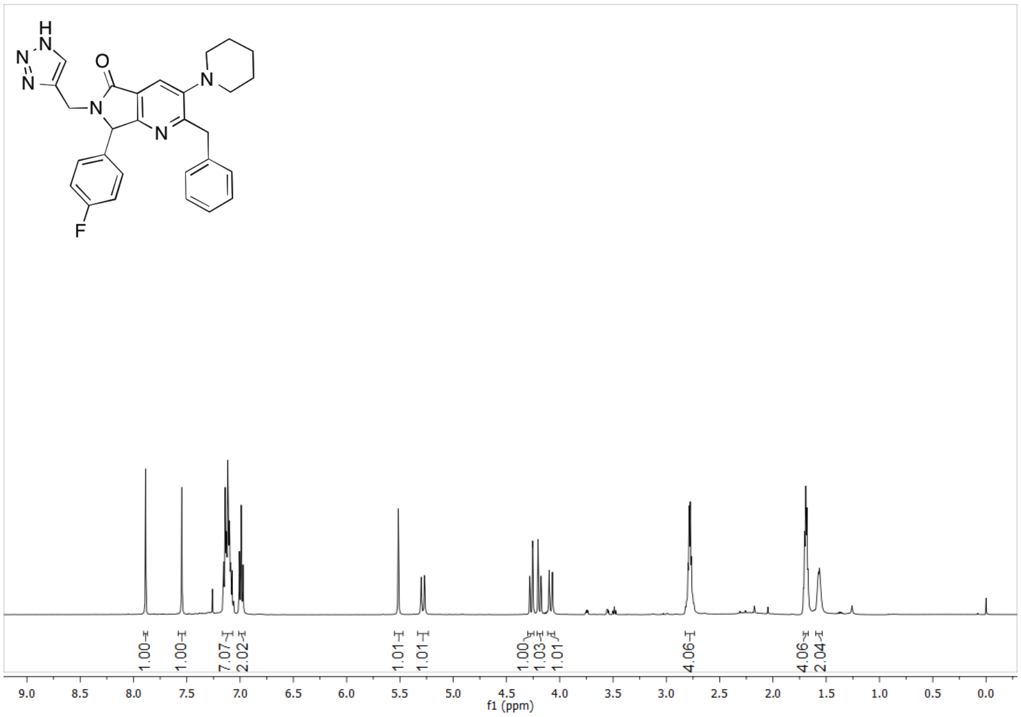

^{13}C NMR **9c**

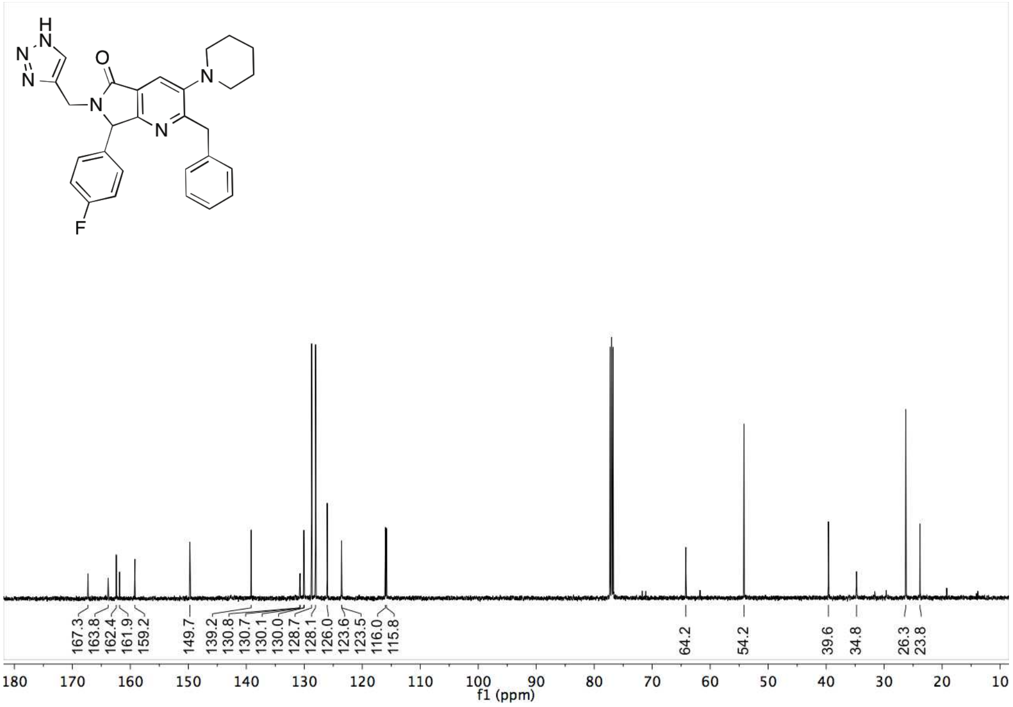

HRMS **9c**

Mass Spectrum SmartFormula Report

Analysis Info

		Acquisition Date	2/28/2017 12:55:04 PM
Analysis Name	D:\Data\Monica Rincon\Eduardo_Gonzalez\20170228_6c_Pos.d		
Method	tune_low_cres_110117MS.m	Operator	CBS_UAM_I
Sample Name	20170228_6c_Pos	Instrument	micrOTOF 213750.00410
Comment	20170228_6c_Pos		

Acquisition Parameter

Source Type	ESI	Ion Polarity	Positive	Set Nebulizer	3.0 Bar
Focus	Active	Set Capillary	4500 V	Set Dry Heater	210 °C
Scan Begin	50 m/z	Set End Plate Offset	-500 V	Set Dry Gas	10.0 l/min
Scan End	3000 m/z	Set Charging Voltage	0 V	Set Divert Valve	Source
		Set Corona	0 nA	Set APCI Heater	0 °C

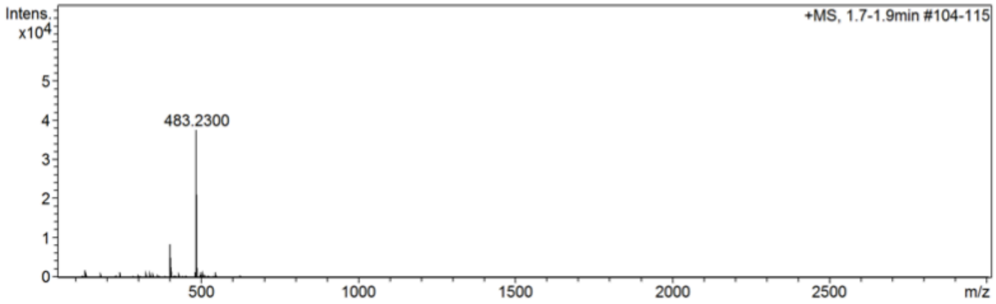

Meas. m/z	#	Ion Formula	m/z	err [ppm]	mSigma	# Sigma	Score	rdb	e⁻ Conf	N-Rule
402.1968	1	C25H25FN3O	402.1976	2.0	3.9	1	98.26	14.5	even	ok
	2	C28H24N3	402.1965	-0.9	14.7	2	100.00	18.5	even	ok
483.2300	1	C28H28FN6O	483.2303	0.7	5.9	1	100.00	17.5	even	ok

6-((1*H*-1,2,3-Triazol-4-yl)methyl)-2-benzyl-7-hexyl-3-(piperidin-1-yl)-6,7-dihydro-5*H*-pyrrolo[3,4-*b*]pyridin-5-one (9d): According to GP-2, pyrrolo[3,4-*b*]pyridin-5-one **6d** (200.0 mg, 0.465 mmol), $TMSN_3$ (123.0 µL, 0.931 mmol), and CuI (3.0 mg, 0.016 mmol), were reacted together in a mixture 0.5 M of DMF/MeOH (9/1 v/v) to afford the 6-((1*H*-1,2,3-triazol-4-yl)methyl)-pyrrolo[3,4-*b*]pyridin-5-one **9d**. Yield 70% (154.0 mg); orange solid; ***R**$_f$* = 0.45 (hexanes/EtOAc, 1:1); **m.p.** 60-61 °C; **FT-IR (ATR)** υ_{max}/cm^{-1} 1670 (C=O); **^{1}H NMR** (500 MHz, $CDCl_3$, 25 °C): δ = 0.67–0.73 (m, 1H), 0.82 (t, *J* = 7.1 Hz, 3H), 1.00–1.06 (m, 1H), 1.10–1.19 (m, 6H), 1.55–1.62 (m, 2H), 1.69–1.74 (m, 4H), 1.94–2.02 (m, 1H), 2.15–2.22 (m, 1H), 2.76–2.83 (m, 4H), 4.24 (d, *J* = 13.9 Hz, 1H), 4.40 (d, *J* = 14.0 Hz, 1H), 4.45 (d, *J* = 15.5 Hz, 1H), 4.49–4.53 (m, 1H), 5.28 (d, *J* = 15.5 Hz, 1H), 7.11–7.29 (m, 5H), 7.68 (s, 1H), 7.82 (s, 1H); **^{13}C NMR** (126 MHz, $CDCl_3$, 25 °C): δ = 14.0, 22.3, 22.5, 23.9, 26.4, 29.1 (2), 31.5, 34.8, 39.7, 54.3, 60.5, 123.3, 124.2, 126.1, 128.1, 128.9, 139.6, 149.2, 159.3, 161.7, 167.6; **HRMS (ESI$^+$):** *m/z* calcd. for $C_{28}H_{37}N_6O^+$ $[M + H]^+$ 473.3023, found 473.3014.

^{1}H NMR **9d**

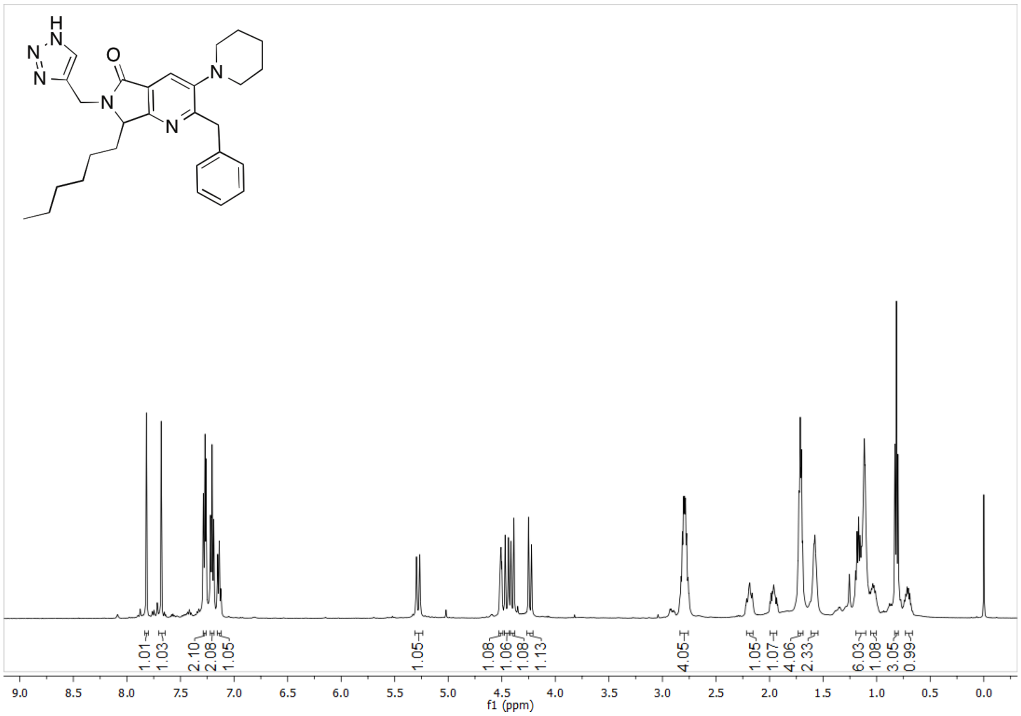

^{13}C NMR **9d**

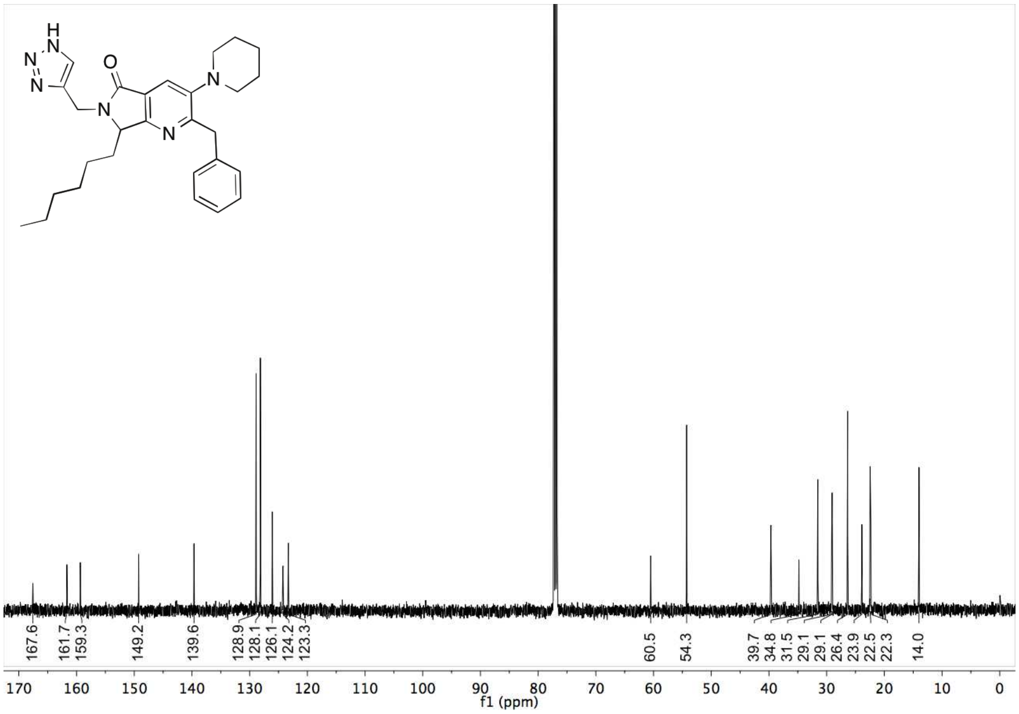

HRMS **9d**

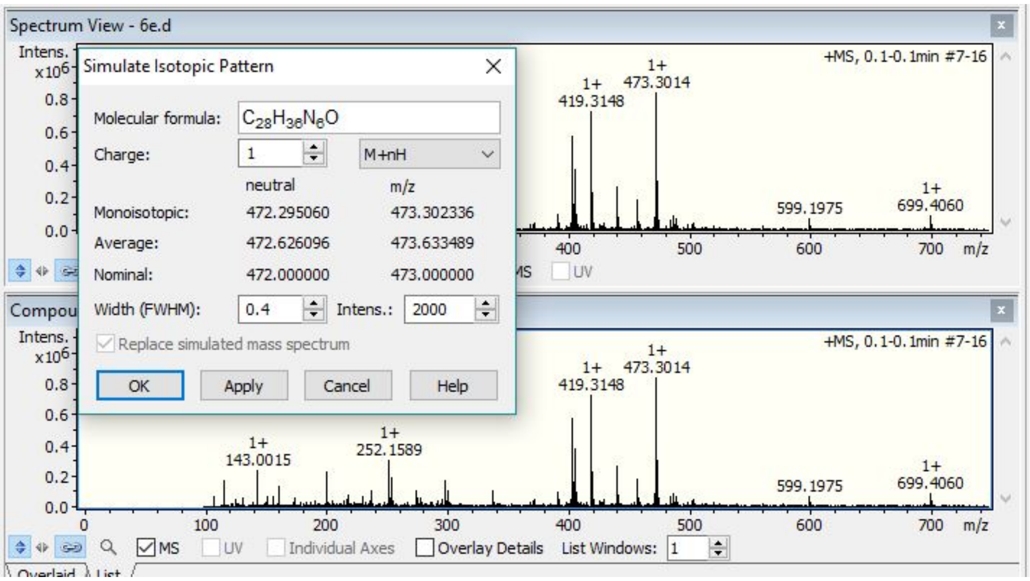

6-((1*H*-1,2,3-Triazol-4-yl)methyl)-2-benzyl-3-morpholino-7-phenyl-6,7-dihydro-5*H*-pyrrolo[3,4-*b*]pyridin-5-one (9e): According to GP-2, pyrrolo[3,4-*b*]pyridin-5-one **6e** (200.0 mg, 0.472 mmol), $TMSN_3$ (125.0 µL, 0.944 mmol), and CuI (3.0 mg, 0.016 mmol), were reacted together in a mixture 0.5 M of DMF/MeOH (9/1 v/v) to afford the 6-((1*H*-1,2,3-triazol-4-yl)methyl)-pyrrolo[3,4-*b*]pyridin-5-one **9e**. Yield 63% (160.0 mg); yellow solid; $\boldsymbol{R_f}$ = 0.25 (hexanes/EtOAc, 3:7); **m.p.** 187-188 °C; **FT-IR (ATR)** υ_{max}/cm^{-1} 1672 (C=O); **^{1}H NMR** (500 MHz, $CDCl_3$, 25 °C): δ = 2.76–2.87 (m, 4H), 3.76–3.83 (m, 4H), 4.12 (d, *J* = 15.4 Hz, 1H), 4.20 (d, *J* = 14.0 Hz, 1H), 4.30 (d, *J* = 14.0 Hz, 1H), 5.29 (d, *J* = 15.4 Hz), 5.54 (s, 1H), 7.06–7.16 (m, 7H), 7.29–7.34 (m, 3H), 7.52 (s, 1H), 7.95 (s, 1H); **^{13}C NMR** (126 MHz, $CDCl_3$, 25 °C): δ = 34.9, 39.9, 52.9, 65.1, 67.0, 123.8, 124.1, 126.1, 128.1, 128.2, 128.6, 128.8, 128.9, 134.8, 139.0, 142.3, 148.0, 160.4, 162.3, 167.1; **HRMS (ESI$^+$):** *m/z* calcd. for $C_{27}H_{27}N_6O_2^+$ $[M + H]^+$ 467.2190, found 467.2201.

^{1}H NMR **9e**

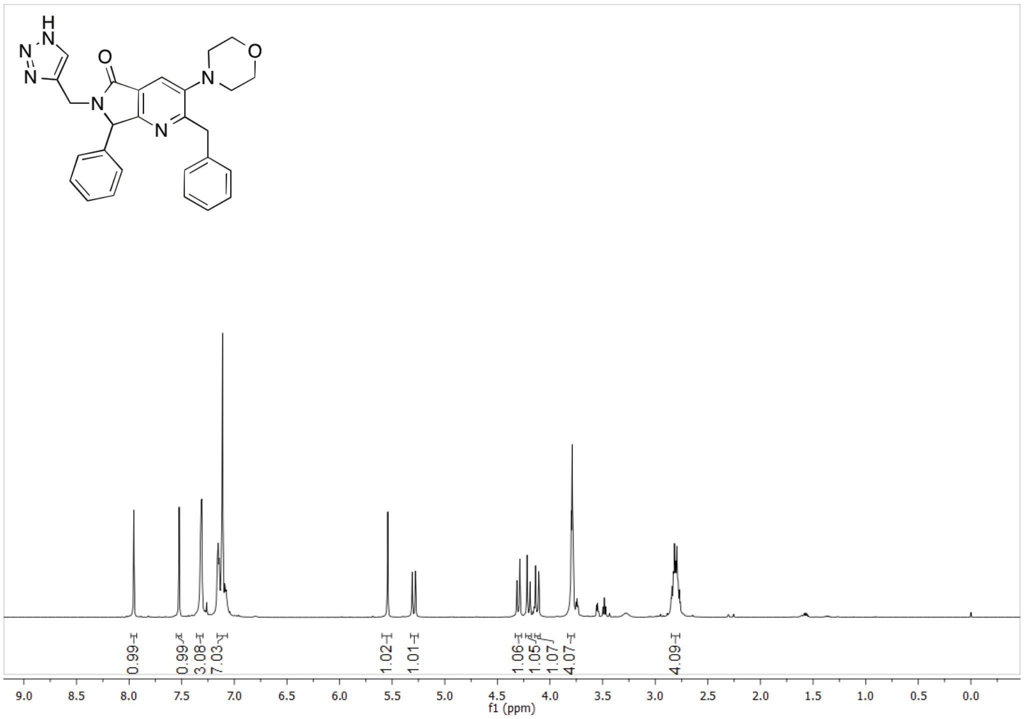

^{13}C NMR **9e**

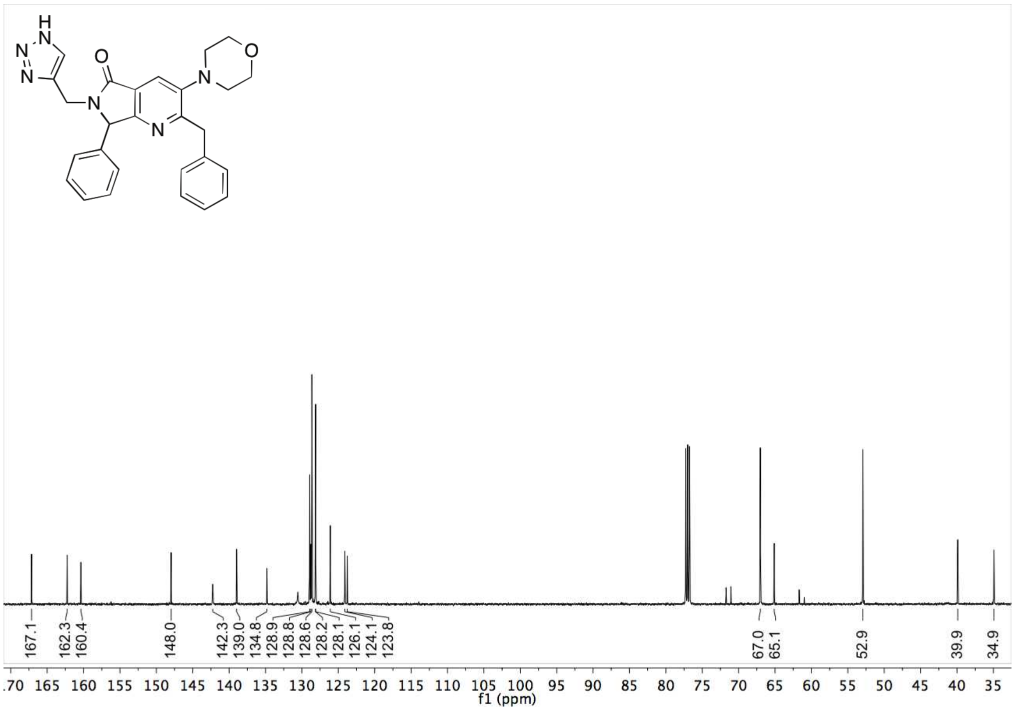

HRMS **9e**

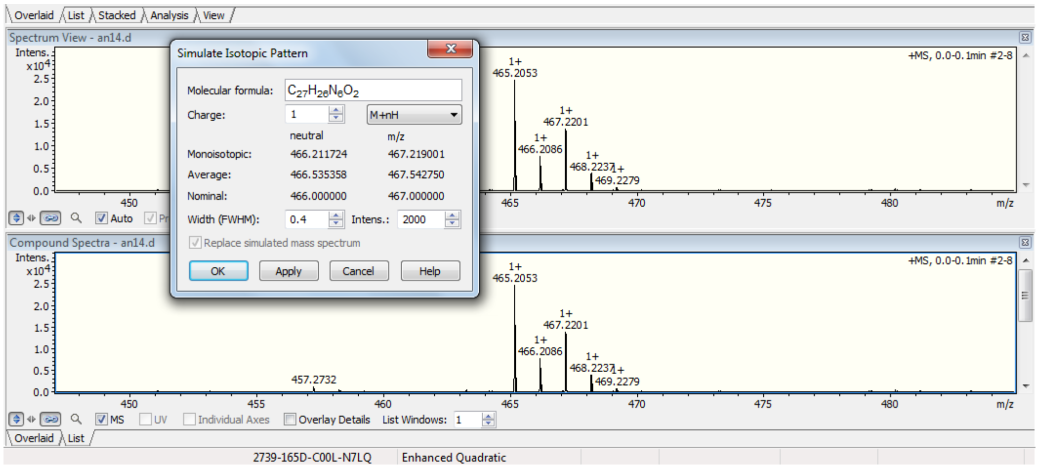

6-((1*H*-1,2,3-Triazol-4-yl)methyl)-2-benzyl-7-(3,4-dimethoxyphenyl)-3-morpholino-6,7-dihydro-5*H*-pyrrolo[3,4-*b*]pyridin-5-one (9f): According to GP-2, pyrrolo[3,4-*b*]pyridin-5-one **6f** (200.0 mg, 0.413 mmol), $TMSN_3$ (109.0 µL, 0.827 mmol), and CuI (3.0 mg, 0.016 mmol), were reacted together in a mixture 0.5 M of DMF/MeOH (9/1 v/v) to afford the 6-((1*H*-1,2,3-triazol-4-yl)methyl)-pyrrolo[3,4-*b*]pyridin-5-one **9f**. Yield 73% (158.0 mg); yellow solid; $\boldsymbol{R_f}$ = 0.12 (hexanes/EtOAc, 3:7); **m.p.** 216-217 °C; **FT-IR (ATR)** υ_{max}/cm^{-1} 1663 (C=O); **^{1}H NMR** (400 MHz, $CDCl_3$, 25 °C): δ = 2.79–2.86 (m, 4H), 3.72 (s, 3H), 3.79–3.84 (m, 4H), 3.86 (s, 3H), 4.16 (d, *J* = 15.6 Hz, 1H), 4.24 (d, *J* = 14.3 Hz, 1H), 4.31 (d, *J* = 13.9 Hz, 1H), 5.29 (d, *J* = 15.5 Hz, 1H), 5.52 (m, 1H), 4.87 (d, *J* = 17.76, 1H), 5.52 (s, 1H), 6.56 (s, 1H), 6.79–6.86 (m, 2H), 7.06–7.18 (m, 5H), 7.59 (s, 1H), 7.95 (s, 1H); **^{13}C NMR** (100 MHz, $CDCl_3$, 25 °C): δ = 34.8, 39.9, 52.9, 55.8, 65.0, 67.0, 110.5, 111.2, 121.1, 123.8, 124.1, 126.2, 126.8, 128.2, 128.6, 139.0, 148.0, 149.3 (2), 160.3, 162.3, 167.0; HRMS (ESI^+): *m/z* calcd. for $C_{29}H_{31}N_6O_4^+$ $[M + H]^+$ 527.2401, found 527.2397.

^{1}H NMR **9f**

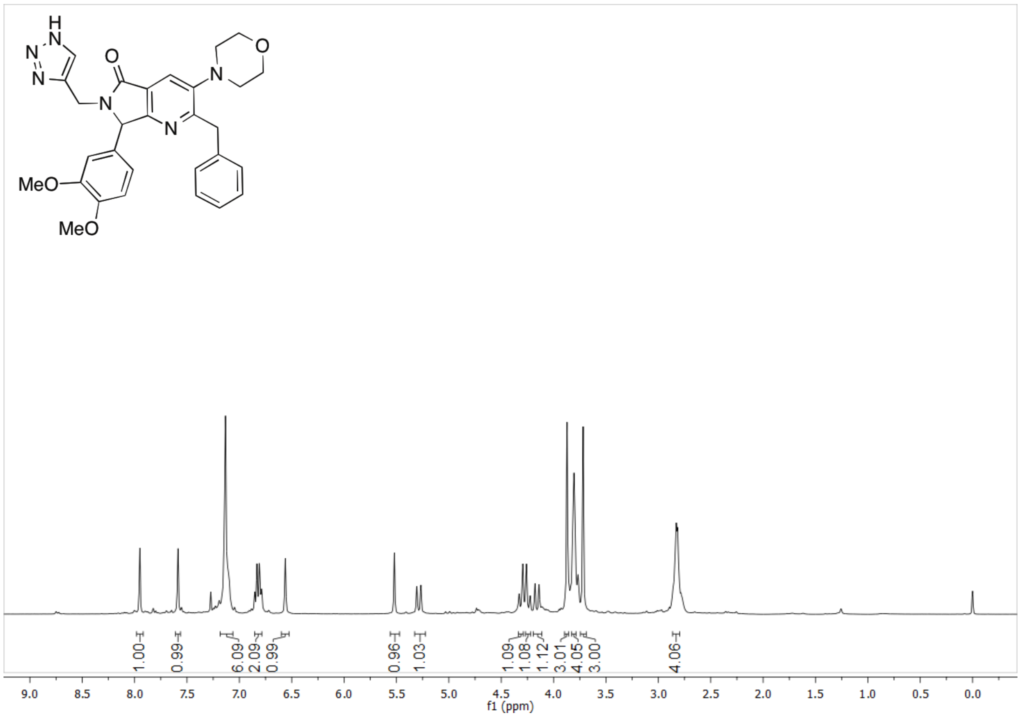

^{13}C NMR **9f**

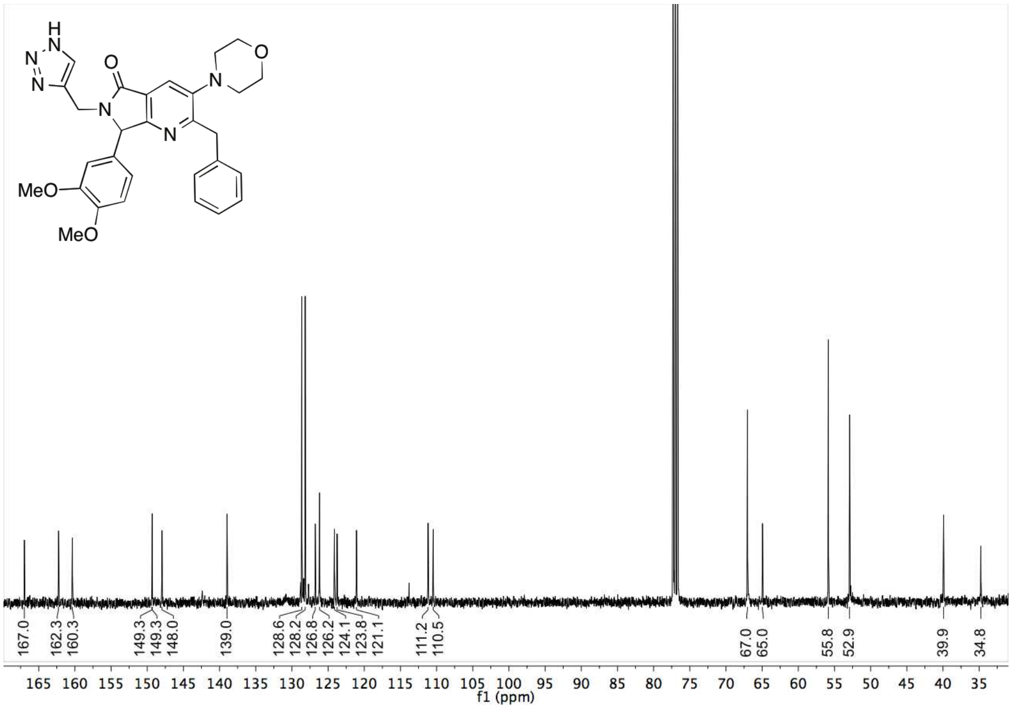

HRMS **9f**

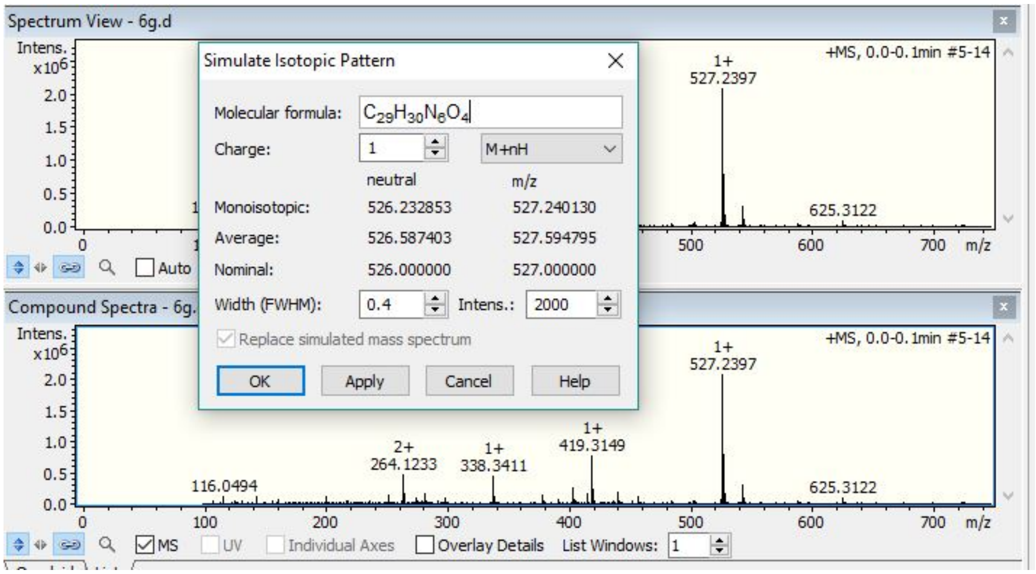

6-((1*H*-1,2,3-Triazol-4-yl)methyl)-2-benzyl-7-(4-fluorophenyl)-3-morpholino-6,7-dihydro-5*H*-pyrrolo[3,4-*b*]pyridin-5-one (9g): According to GP-2, pyrrolo[3,4-*b*]pyridin-5-one **6g** (200.0 mg, 0.453 mmol), $TMSN_3$ (126.0 µL, 0.906 mmol), and CuI (3.0 mg, 0.016 mmol), were reacted together in a mixture 0.5 M of DMF/MeOH (9/1 v/v) to afford the 6-((1*H*-1,2,3-triazol-4-yl)methyl)-pyrrolo[3,4-*b*]pyridin-5-one **9g**. Yield 80% **(**175.0 mg); yellow oil; $\boldsymbol{R_f}$ = 0.20 (hexanes/EtOAc, 3:7); **m.p.** 166-167 °C; **FT-IR (ATR)** υ_{max}/cm^{-1} 1663 (C=O); **^{1}H NMR** (500 MHz, $CDCl_3$, 25 °C): δ = 2.77–2.86 (m, 4H), 3.78–3.82 (m, 4H), 4.11 (d, *J* = 15.5 Hz, 1H), 4.22 (d, *J* = 14.1 Hz, 1H), 4.30 (d, *J* = 14.0 Hz, 1H), 5.29 (d, *J* = 15.5 Hz, 1H), 5.54 (s, 1H), 6.98–7.03 (m, 2H), 7.09–7.16 (m, 7H), 7.57 (s, 1H), 7.95 (s, 1H); **^{13}C NMR** (126 MHz, $CDCl_3$, 25 °C): δ = 34.9. 39.9, 52.9, 64.3, 67.0, 115.9, 116.0, 123.7, 124.1, 126.2, 128.2, 128.6, 129.9, 130.0, 130.6 (2), 138.9, 148.1, 160.1, 161.9, 162.4, 163.9, 167.0. **HRMS (ESI$^+$):** *m/z* calcd. for $C_{27}H_{26}FN_6O_2^+$ $[M + H]^+$ 485.2096, found 485.2095.

^{1}H NMR **9g**

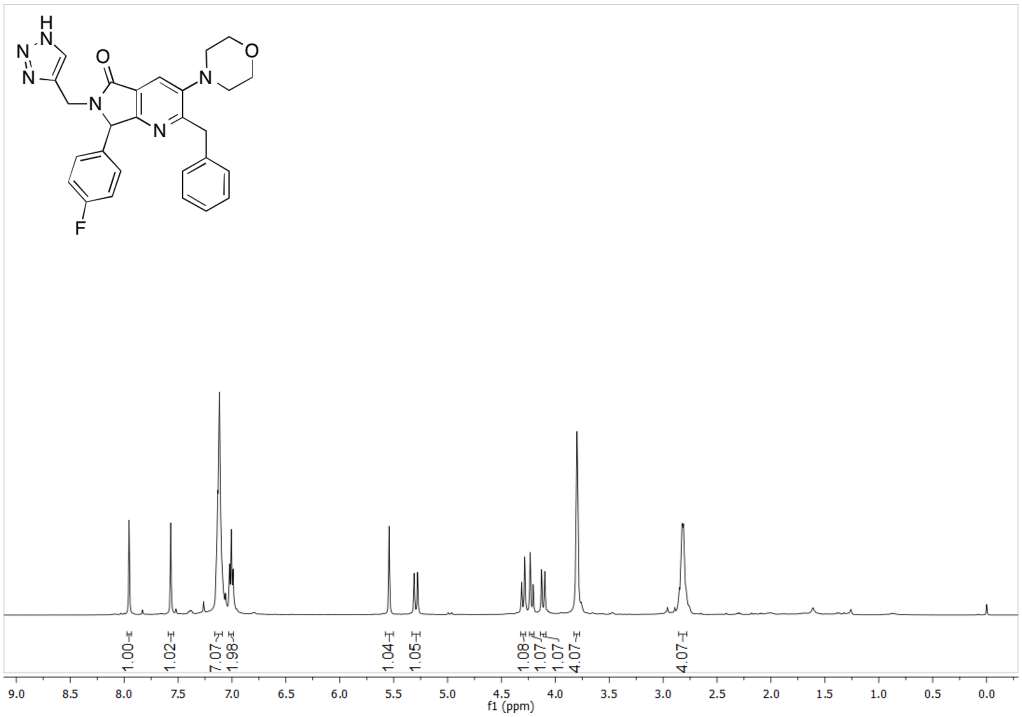

^{13}C NMR **9g**

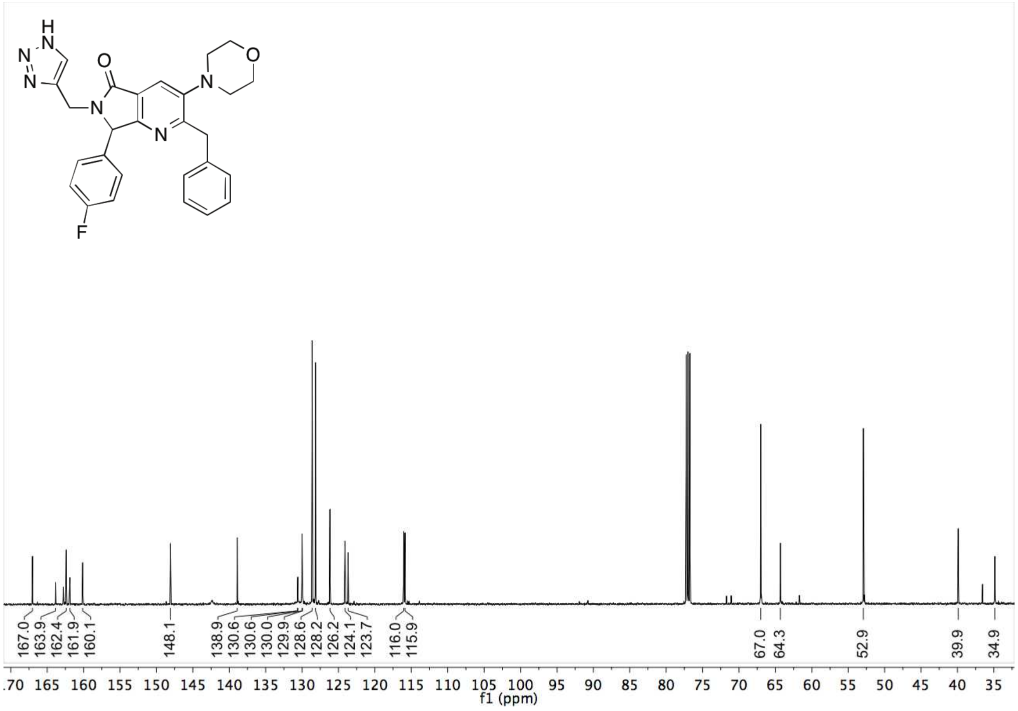

HRMS **9g**

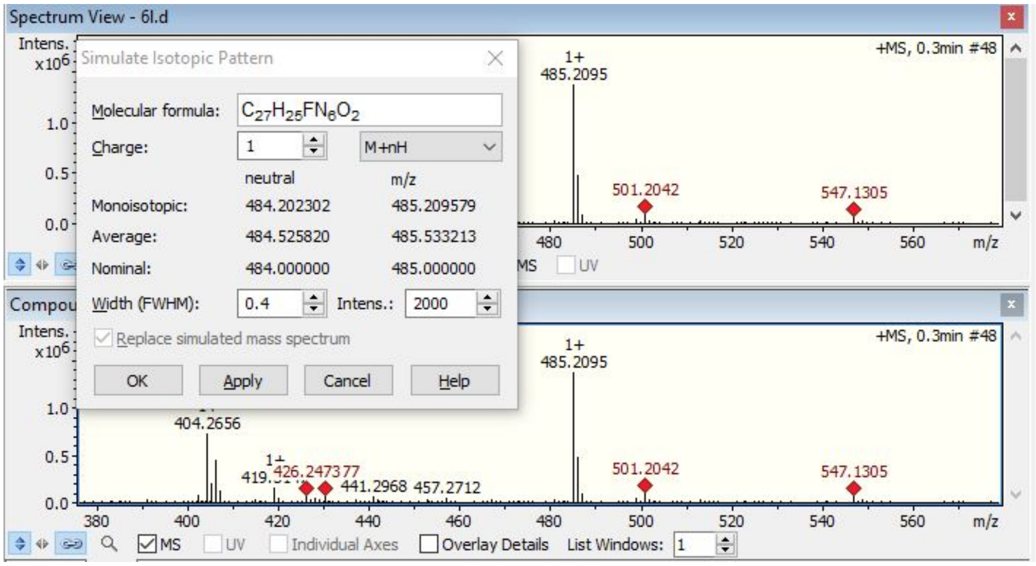

6-((1*H*-1,2,3-Triazol-4-yl)methyl)-2-benzyl-7-hexyl-3-morpholino-6,7-dihydro-5*H*-pyrrolo[3,4-*b*]pyridin-5-one (9h): According to GP-2, pyrrolo[3,4-*b*]pyridin-5-one **6h** (200.0 mg, 0.463 mmol), $TMSN_3$ (123.0 µL, 0.927 mmol), and CuI (3.0 mg, 0.016 mmol), were reacted together in a mixture 0.5 M of DMF/MeOH (9/1 v/v) to afford the 6-((1*H*-1,2,3-triazol-4-yl)methyl)-pyrrolo[3,4-*b*]pyridin-5-one **9h**. Yield 75%, (164.0 mg); orange oil; $\boldsymbol{R_f}$ = 0.17 (hexanes/EtOAc, 1:1); **FT-IR (ATR)** υ_{max}/cm^{-1} 1661 (C=O); **^{1}H NMR** (500 MHz, $CDCl_3$, 25 °C): δ = 0.67–0.78 (m, 6H), 0.82 (t, *J* = 7.1 Hz, 3H), 1.01–1.09 (m, 6H), 1.10–1.22 (m, 6H), 1.97–2.02 (m, 1H), 2.16–2.26 (m, 1H), 2.88–2.79 (m, 4H), 3.86–3.79 (m, 4H), 4.29 (d, *J* = 14.1 Hz, 1H), 4.42 (d, *J* = 14.1 Hz, 1H), 4.48 (d, *J* = 15.5 Hz, 1H), 4.54–4.58 (m, 1H), 5.31 (d, *J* = 15.5 Hz, 1H), 7.12–7.25 (m, 5H), 7.69 (s, 1H), 7.89 (s, 1H); **^{13}C NMR** (126 MHz, $CDCl_3$, 25 °C): δ = 14.0, 22.4, 22.5, 29.1 (2), 31.5, 35.0, 39.9, 53.1, 60.6, 67.2, 123.9, 124.5, 126.3 (2), 128.8, 139.4, 147.7, 160.3, 161.7, 167.3; **HRMS (ESI$^+$):** *m/z* calcd. for $C_{27}H_{35}N_6O_2^+$ $[M + H]^+$ 475.2816, found 475.2815.

^{1}H NMR **9h**

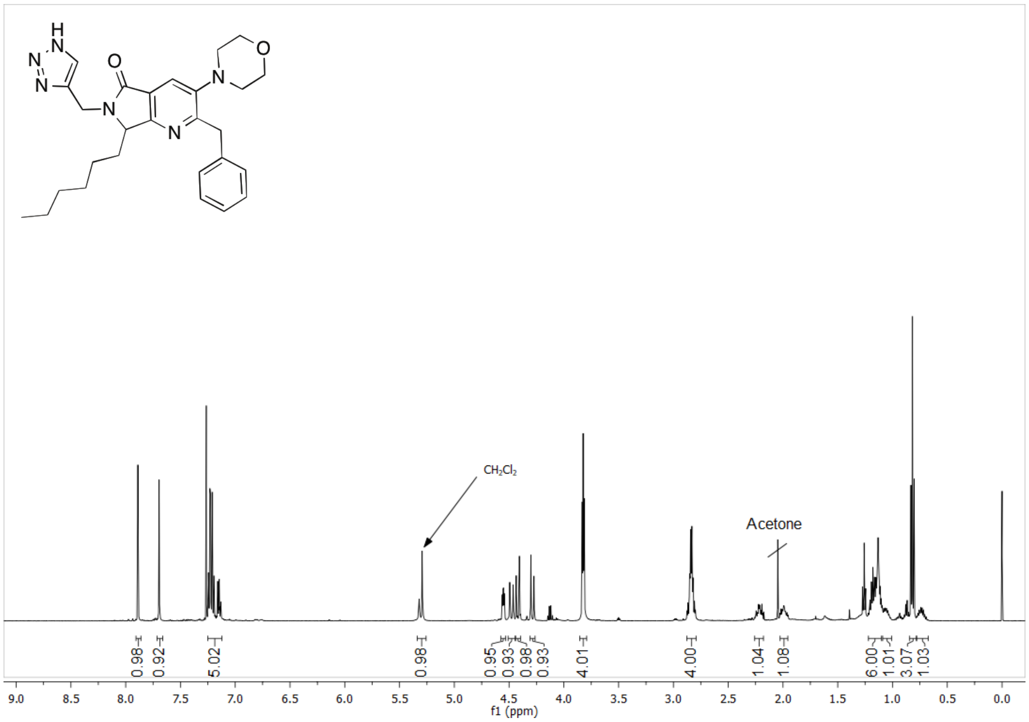

^{13}C NMR **9h**

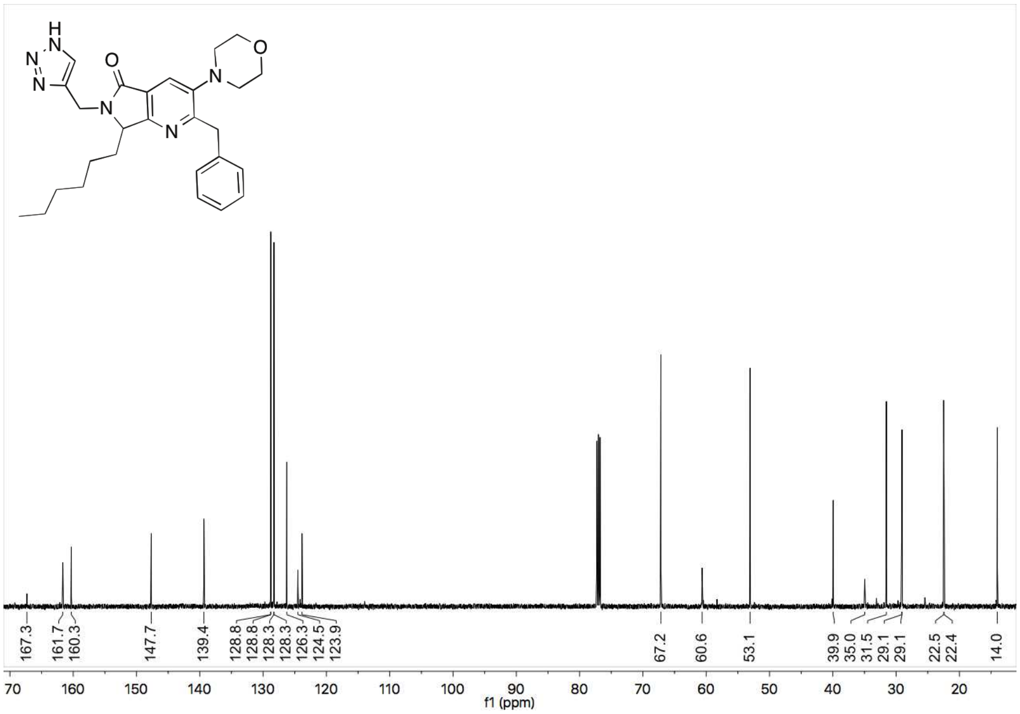

HRMS **9h**

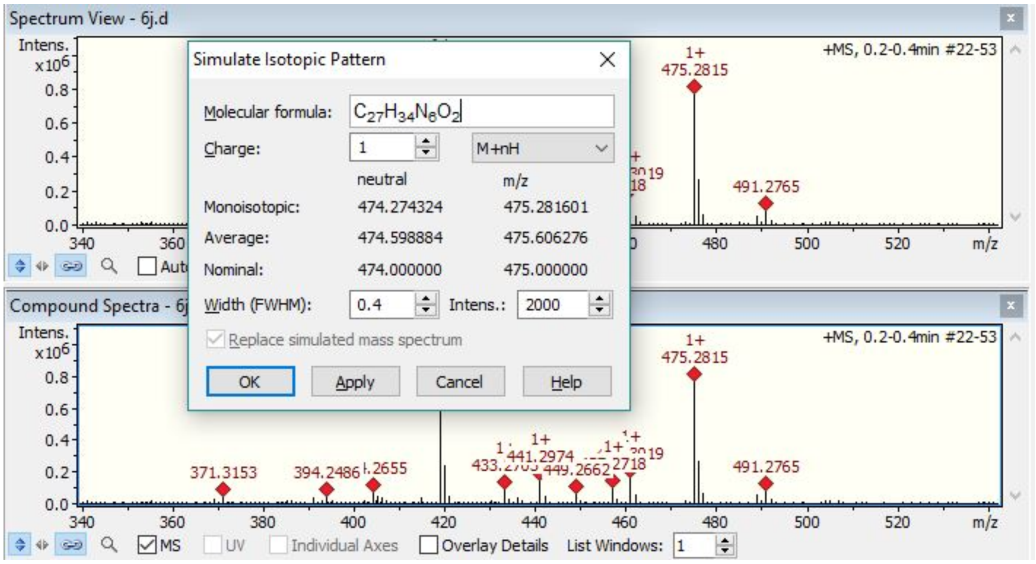

6-((1*H*-1,2,3-Triazol-4-yl)methyl)-2-benzyl-3-(diethylamino)-7-phenyl-6,7-dihydro-5*H*-pyrrolo[3,4-*b*]pyridin-5-one (9i): According to GP-2, pyrrolo[3,4-*b*]pyridin-5-one **6i** (200.0 mg, 0.488 mmol), $TMSN_3$ (129.0 µL, 0.976 mmol), and CuI (3.0 mg, 0.016 mmol), were reacted together in a mixture 0.5 M DMF/MeOH (9/1 v/v) to afford the 6-((1*H*-1,2,3-triazol-4-yl)methyl)-pyrrolo[3,4-*b*]pyridin-5-one **9i**. Yield 73% (164.0 mg); yellow gum; R_f = 0.14 (hexanes/EtOAc, 1:1); **FT-IR (ATR)** υ_{max}/cm^{-1} 1670 (C=O); **^{1}H NMR** (400 MHz, $CDCl_3$, 25 °C): δ = 0.80 (t, *J* = 7.1 Hz, 6H), 2.87 (q, *J* = 7.1 Hz, 4H), 4.01 (d, *J* = 15.5 Hz, 1H), 4.11 (d, *J* = 14.1 Hz, 1H), 5.23 (d, *J* = 14.1 Hz, 1H), 5.22 (d, *J* = 15.5 Hz, 1H), 5.47 (s, 1H), 6.93–7.07 (m, 7H), 7.17–7.22 (m, 3H), 7.41 (s, 1H), 7.86 (s, 1H); **^{13}C NMR** (101 MHz, $CDCl_3$, 25 °C): δ = 12.1, 34.9, 39.7, 47.7, 65.1, 123.5, 26.0, 126.1, 128.0, 128.3, 128.8, 128.9, 129.0, 134.9, 139.3, 146.9, 159.7, 163.8, 167.5; **HRMS (ESI$^+$):** *m/z* calcd. for $C_{27}H_{28}N_3O^+$ [M + H]$^+$ 453.2397, found 453.2407.

^{1}H NMR **9i**

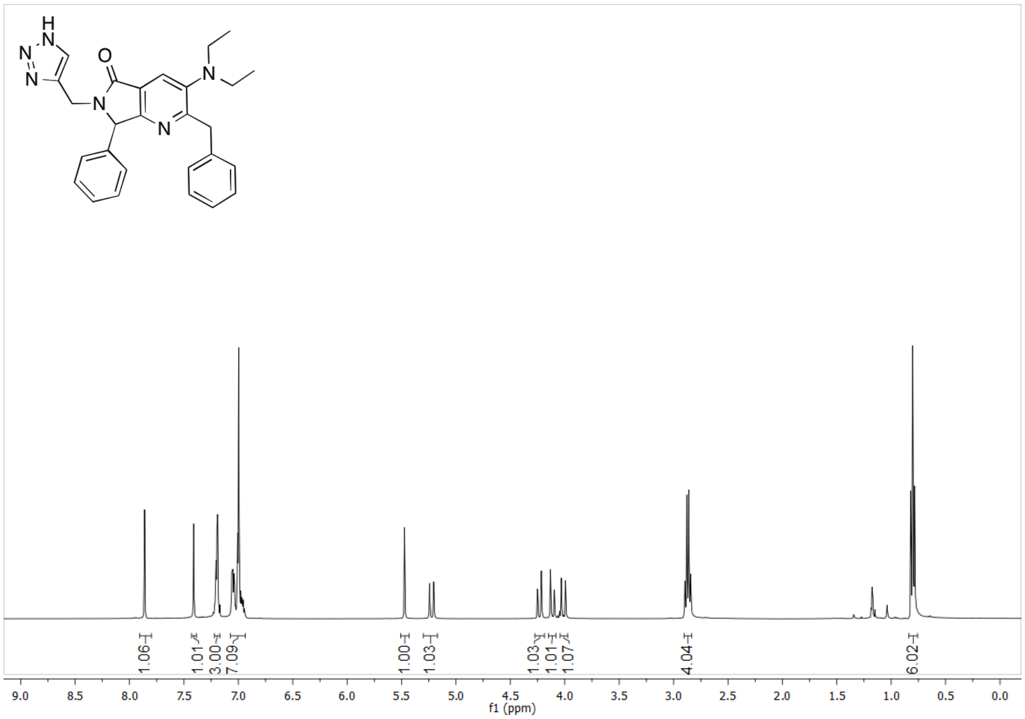

^{13}C NMR **9i**

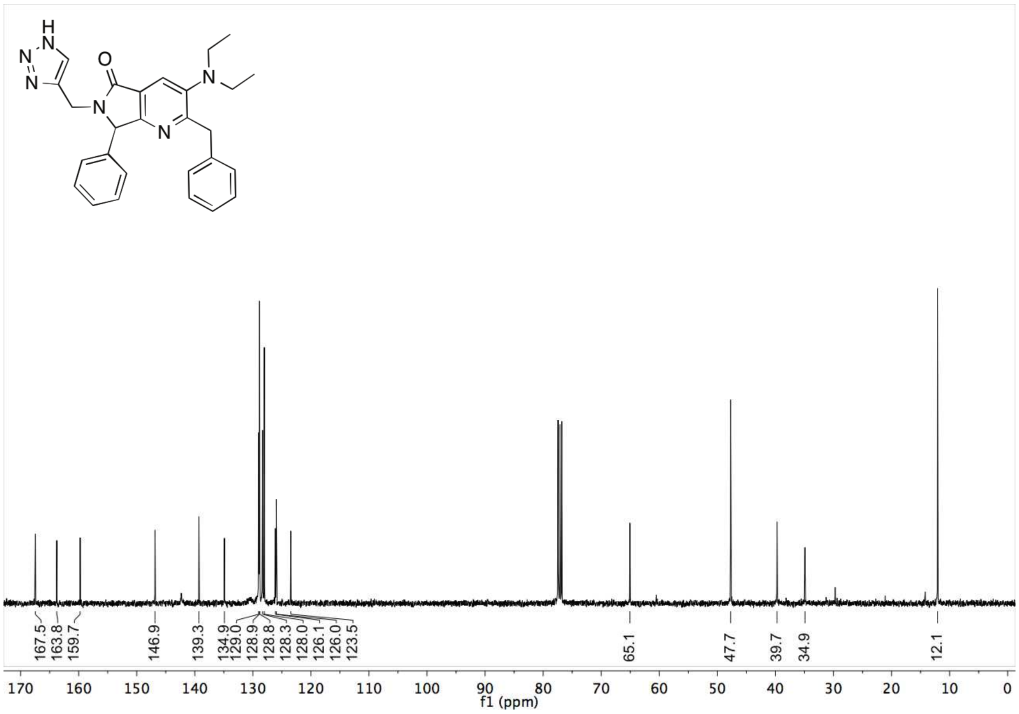

HRMS **9i**

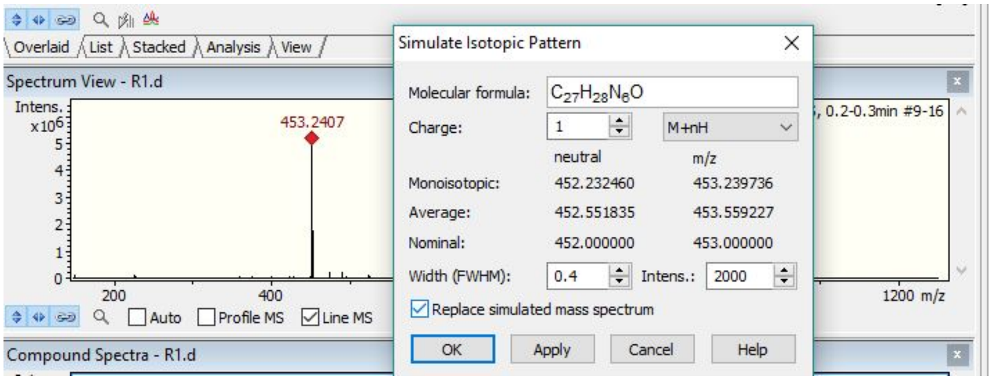

6-((1*H*-1,2,3-Triazol-4-yl)methyl)-2-benzyl-3-(diethylamino)-7-(3,4-dimethoxyphenyl)-6,7-dihydro-5*H*-pyrrolo[3,4-*b*]pyridin-5-one (9j): According to GP-2, pyrrolo[3,4-*b*]pyridin-5-one **6j** (200.0 mg, 0.425 mmol), $TMSN_3$ (113.0 µL, 0.851 mmol), and CuI (3.0 mg, 0.016 mmol), were reacted together in a mixture 0.5 M of DMF/MeOH (9/1 v/v) to afford the 6-((1*H*-1,2,3-triazol-4-yl)methyl)-pyrrolo[3,4-*b*]pyridin-5-one **9j**. Yield 70% **(**155.0 mg); yellow gum; $\boldsymbol{R_f}$ = 0.33 (hexanes/EtOAc, 3:7); **FT-IR (ATR)** υ_{max}/cm^{-1} 1667 (C=O); **^{1}H NMR** (500 MHz, $CDCl_3$, 25 °C): δ = 0.89 (t, *J* = 7.1 Hz, 6H), 2.96 (q, *J* = 7.2 Hz, 4H), 3.69 (s, 3H), 3.86 (s, 3H), 4.14 (d, *J* = 15.4 Hz, 1H), 4.24 (d, *J* = 14.1 Hz, 1H), 4.30 (d, *J* = 14.1 Hz, 1H), 5.30 (d, *J* = 15.4 Hz, 1H), 5.53 (s, 1H), 6.67 (s, 1H), 6.78 (d, *J* = 8.3 Hz, 1H), 6.83 (d, *J* = 8.4 Hz, 1H), 7.04–7.13 (m, 5H), 7.57 (s, 1H), 7.91 (s, 1H); **^{13}C NMR** (126 MHz, $CDCl_3$, 25 °C): δ = 12.0. 34.7, 39.8, 47.7, 55.8, 55.9 64.8, 110.7, 111.3, 121. 0, 123.3, 125.9, 126.0, 127.1, 127.9, 128.8 139.3, 146.7, 149.3, 159.7, 163.7, 167.2; **HRMS (ESI$^+$):** *m/z* calcd. for $C_{29}H_{33}N_3O_3^+$ $[M + H]^+$ 513.2609, found 513.2605.

^{1}H NMR **9j**

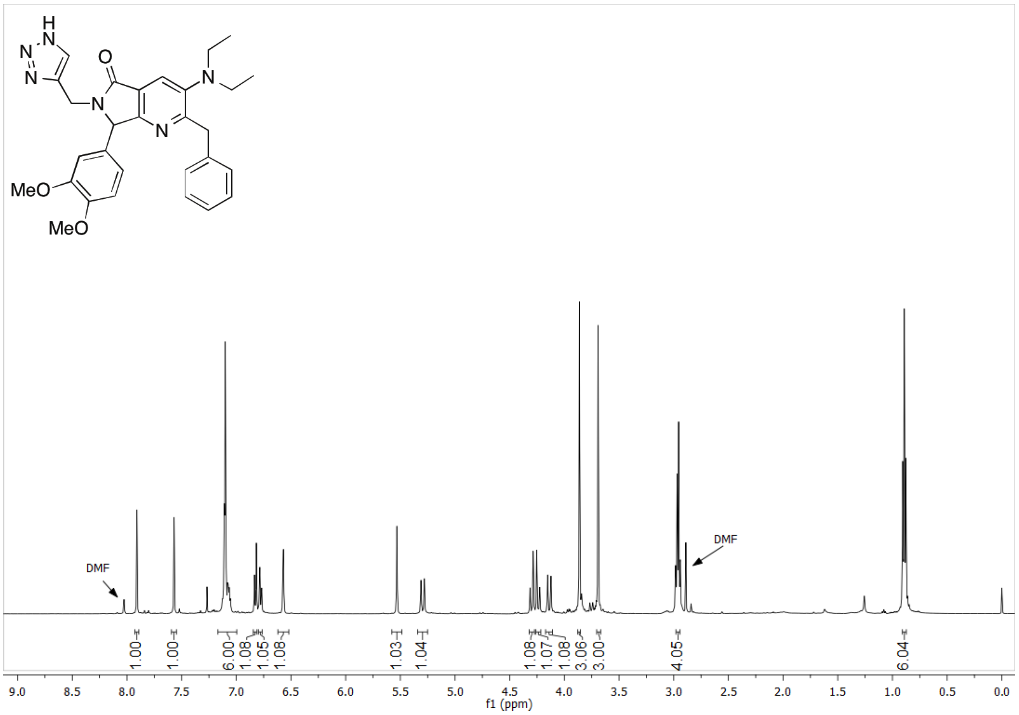

^{13}C NMR **9j**

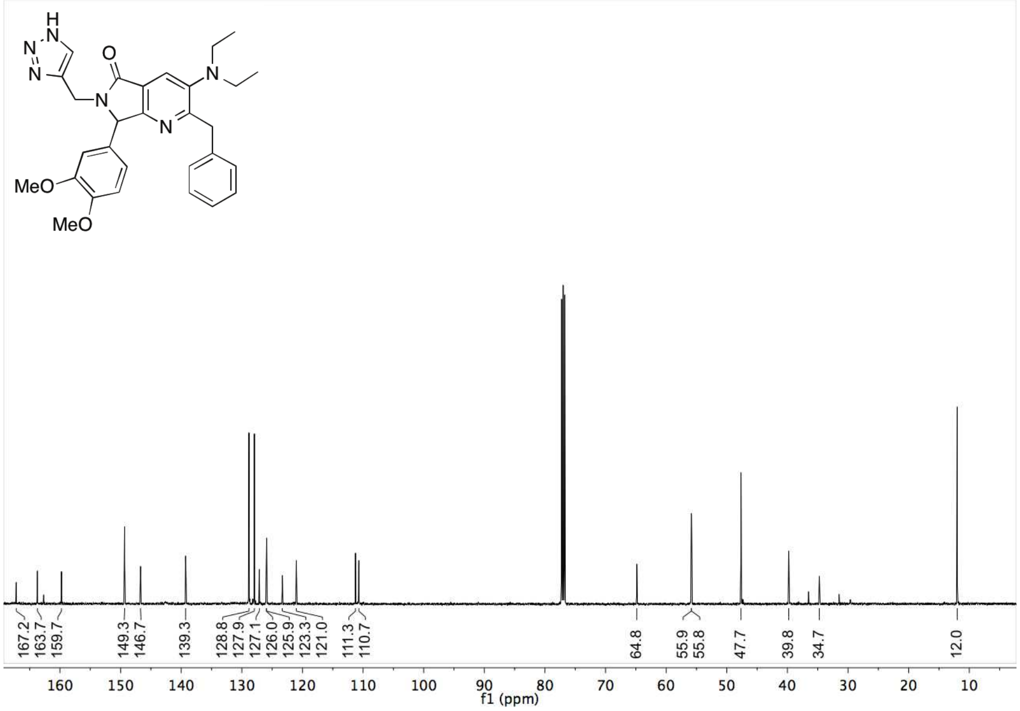

HRMS **9j**

Compound Spectrum SmartFormula Report

Analysis Info

Analysis Name	D:\Data\Monica Rincon\Eduardo_Gonzalez\20170228_6j_Pos.d	Acquisition Date	2/28/2017 2:10:38 PM
Method	tune_low_cres_110117MS.m	Operator	CBS_UAM_I
Sample Name	20170228_6j_Pos	Instrument	micrOTOF 213750.00410
Comment	20170228_6j_Pos		

Acquisition Parameter

Source Type	ESI	Ion Polarity	Positive	Set Nebulizer	3.0 Bar
Focus	Active	Set Capillary	4500 V	Set Dry Heater	210 °C
Scan Begin	50 m/z	Set End Plate Offset	-500 V	Set Dry Gas	10.0 l/min
Scan End	3000 m/z	Set Charging Voltage	0 V	Set Divert Valve	Source
		Set Corona	0 nA	Set APCI Heater	0 °C

+MS, 1.6-1.9min #98-116

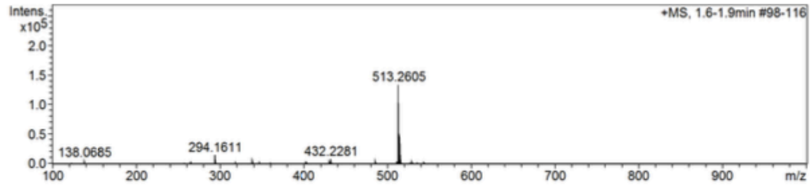

Meas. m/z	#	Ion Formula	m/z	err [ppm]	mSigma	# Sigma	Score	rdb	e⁻ Conf	N-Rule
294.1611	1	C18H20N3O	294.1601	-3.4	11.9	1	100.00	10.5	even	ok
432.2281	1	C26H30N3O3	432.2282	0.3	7.6	1	100.00	13.5	even	ok
485.2296	1	C27H29N6O3	485.2296	-0.0	16.7	1	100.00	16.5	even	ok
	2	C26H33N2O7	485.2282	-2.7	20.0	2	47.54	11.5	even	ok
513.2605	1	C29H33N6O3	513.2609	0.7	14.9	1	100.00	16.5	even	ok

6-((1*H*-1,2,3-Triazol-4-yl)methyl)-2-benzyl-3-(diethylamino)-7-(4-fluorophenyl)-6,7-dihydro-5*H*-pyrrolo[3,4-*b*]pyridin-5-one (9k): According to GP-2, pyrrolo[3,4-*b*]pyridin-5-one **6k** (200.0 mg, 0.467 mmol), $TMSN_3$ (124.0 µL, 0.935 mmol), and CuI (3.0 mg, 0.016 mmol), were reacted together in a mixture 0.5 M of DMF/MeOH (9/1 v/v) to afford the 6-((1*H*-1,2,3-triazol-4-yl)methyl)-pyrrolo[3,4-*b*]pyridin-5-one **9k**. Yield 78% (167.0 mg); yellow gum; $\boldsymbol{R_f}$ = 0.64 (hexanes/EtOAc, 3:7); **FT-IR (ATR)** υ_{max}/cm^{-1} 1673 (C=O); **^{1}H NMR** (400 MHz, $CDCl_3$, 25 °C): δ = 0.82 (t, *J* = 7.1 Hz, 6H), 2.89 (q, *J* = 7.1 Hz, 4H), 4.02 (d, *J* = 15.5 Hz, 1H), 4.13 (d, *J* = 14.1 Hz, 1H), 4.23 (d, *J* = 14.1 Hz, 1H), 5.23 (d, *J* = 15.5 Hz, 1H), 5.47 (s, 1H), 6.86–7.10 (m, 9H), 7.48 (s, 1H), 7.84 (s, 1H); **^{13}C NMR** (101 MHz, $CDCl_3$, 25 °C): δ = 12.1, 34.8, 39.7, 47.7, 64.3, 115.9, 16.1, 123.3, 126 (2), 128.0, 128.9, 130.0, 131.1, 130.7 (2), 139.2, 142.4, 146.9, 159.4, 161.7, 163.9, 164.1, 167.3; **HRMS (ESI$^+$):** *m/z* calcd. for $C_{27}H_{28}FN_6O^+$ $[M + H]^+$ 471.2303, found 471.2304.

^{1}H NMR **9k**

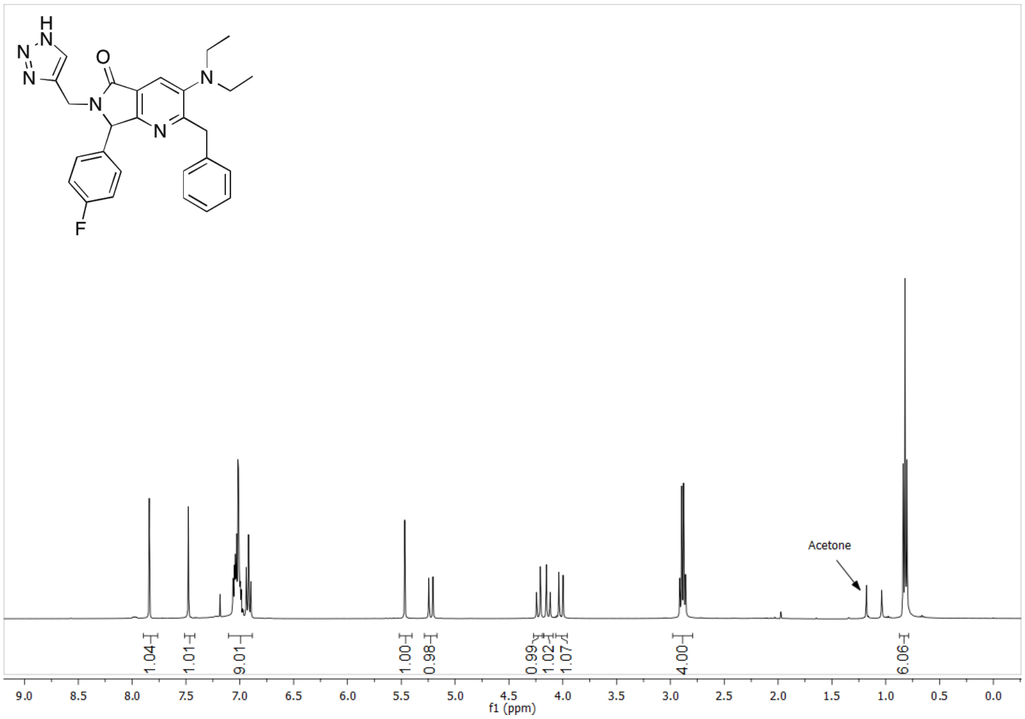

^{13}C NMR **9k**

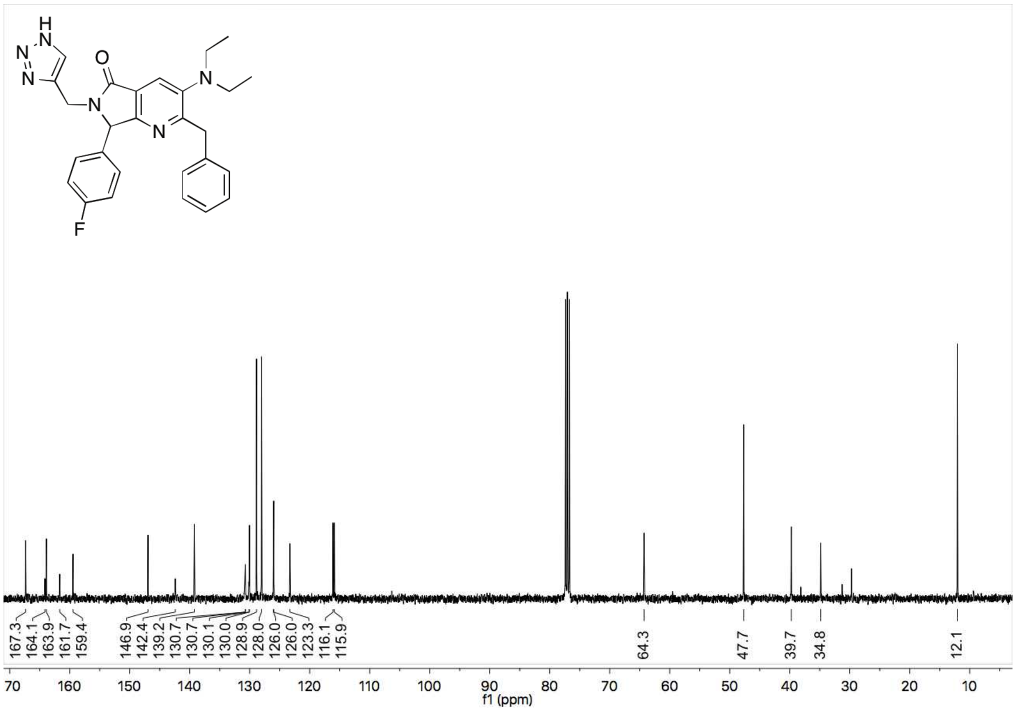

HRMS **6k**

Mass Spectrum SmartFormula Report

Analysis Info

Analysis Name	D:\Data\Monica Rincon\Eduardo_Gonzalez\20170228_6k_Pos.d	Acquisition Date	2/28/2017 2:21:40 PM
Method	tune_low_cres_110117MS.m	Operator	CBS_UAM_I
Sample Name	20170228_6k_Pos	Instrument	micrOTOF 213750.00410
Comment	20170228_6k_Pos		

Acquisition Parameter

Source Type	ESI	Ion Polarity	Positive	Set Nebulizer	3.0 Bar
Focus	Active	Set Capillary	4500 V	Set Dry Heater	210 °C
Scan Begin	50 m/z	Set End Plate Offset	-500 V	Set Dry Gas	10.0 l/min
Scan End	3000 m/z	Set Charging Voltage	0 V	Set Divert Valve	Source
		Set Corona	0 nA	Set APCI Heater	0 °C

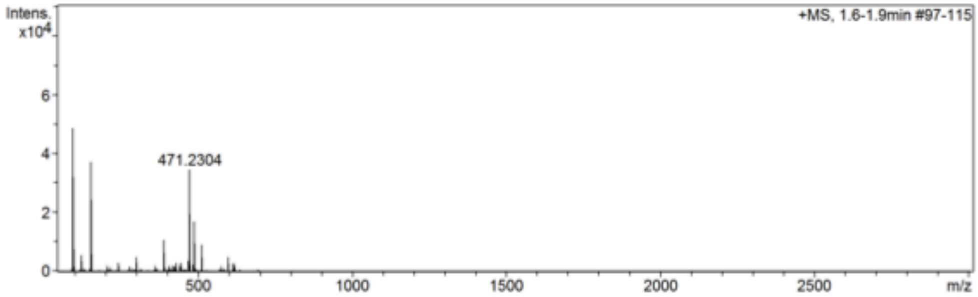

Meas. m/z	#	Ion Formula	Sum Formula	m/z	err [ppm]	mSigma	# Sigma
388.1822	1	C27H22N3	C27 H21 N3	388.1808	3.6	157.3	1
	2	C24H23FN3O	C24 H22 F N3 O	388.1820	-0.6	162.9	2
443.2045	1	C23H32NaO7	C23 H32 O7	443.2040	1.1	150.3	1
471.2304	1	C27H28FN6O	C27 H27 F N6 O	471.2303	0.1	4.8	1

6-((1*H*-1,2,3-Triazol-4-yl)methyl)-2-benzyl-3-(diethylamino)-7-hexyl-6,7-dihydro-5*H*-pyrrolo[3,4-*b*]pyridin-5-one (9l): According to GP-2, pyrrolo[3,4-*b*]pyridin-5-one **6l** (200.0 mg, 0.478 mmol), $TMSN_3$ (127.0 µL, 0.957 mmol), and CuI (3.0 mg, 0.016 mmol), were reacted together in a mixture 0.5 M of DMF/MeOH (9/1 v/v) to afford the 6-((1*H*-1,2,3-triazol-4-yl)methyl)-pyrrolo[3,4-*b*]pyridin-5-one **9l**.Yield 75% (165.0 mg); yellow gum; $\boldsymbol{R_f}$ = 0.35 (hexanes/EtOAc, 1:1); **FT-IR (ATR)** υ_{max}/cm^{-1} 1664 (C=O); ^{1}**H NMR** (500 MHz, $CDCl_3$, 25 °C): δ = 0.64-0.70 (m, 1H), 0.75 (t, *J* = 7.1 Hz, 6H), 0.85 (t, *J* = 7.1 Hz, 6H), 0.95–1.00 (m, 1H), 1.04–1.13 (m, 6H), 1.89–1.95 (m, 1H), 2.09–2.16 (m, 1H), 2.88–2.93 (m, 4H), 4.20 (d, *J* = 14.2 Hz, 1H), 4.35–4.41 (m, 1H), 4.45–4.48 (m, 1H), 5.24 (d, *J* = 15.5, 1H), 7.04–7.17 (m, 5H), 7.62 (s, 1H), 7.79 (s, 1H); ^{13}**C NMR** (126 MHz, $CDCl_3$, 25 °C): δ = 12.1. 14.0, 22.4 (2), 29.0, 29.1, 31.5, 34.8, 39.7, 47.9, 60.5, 124.0, 125.9, 126.0, 128.1, 129.0, 139.6, 146.3, 159.7, 163.2, 167.5; **HRMS (ESI$^+$):** *m/z* calcd. for $C_{27}H_{37}N_6O^+$ [M + H]$^+$ 461.3023, found 461.3021.

^{1}H NMR **9l**

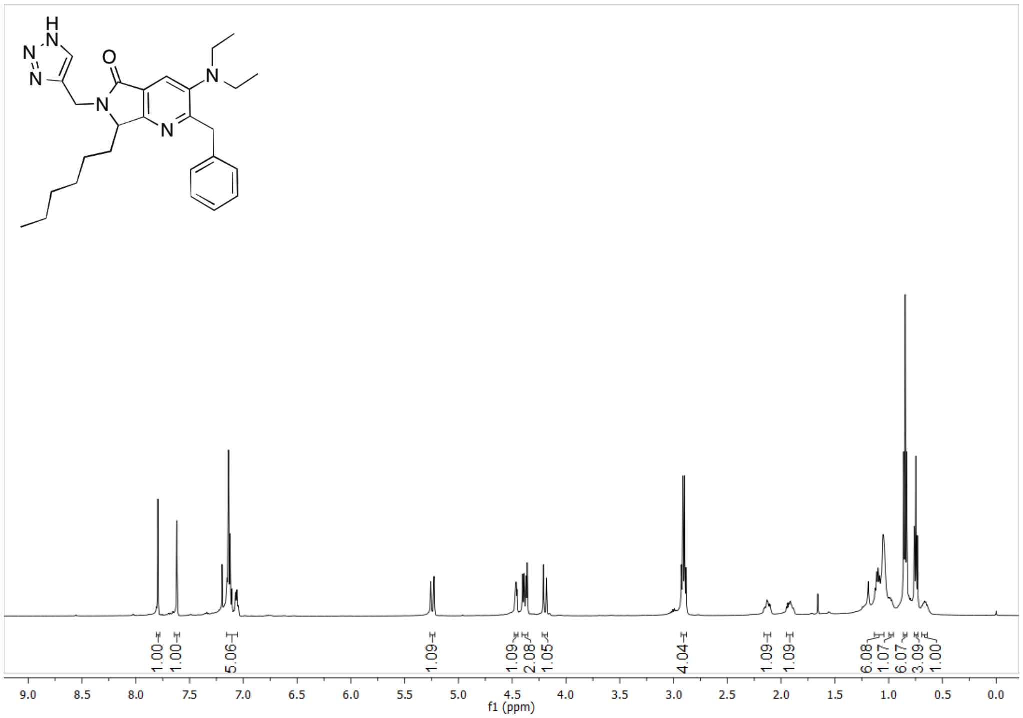

^{13}C NMR **9l**

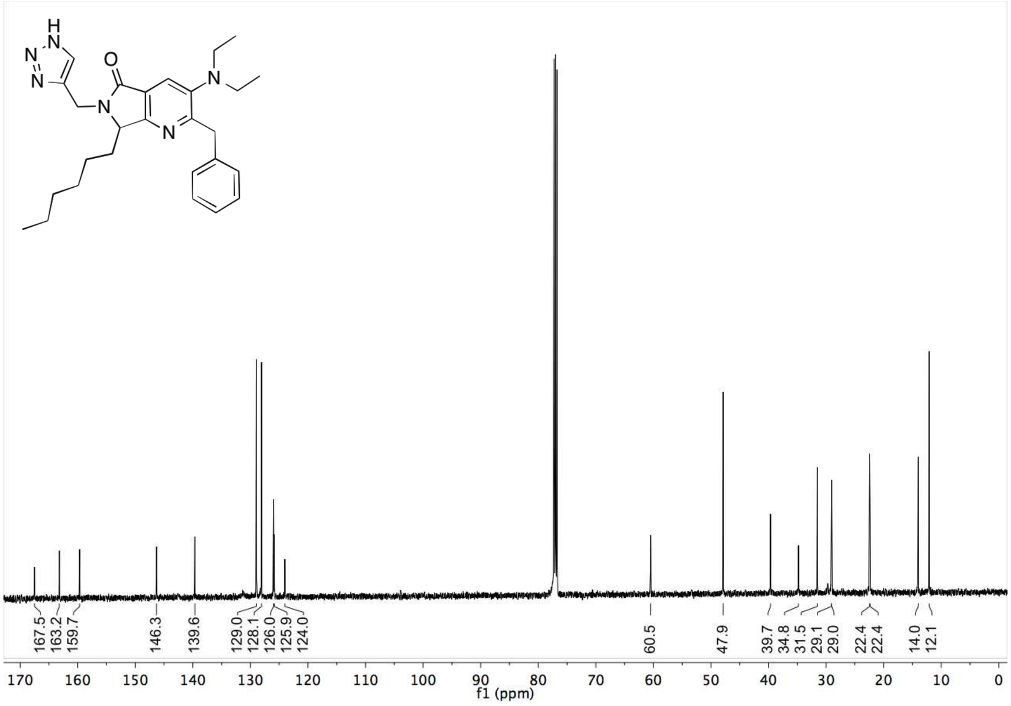

HMRS **9l**

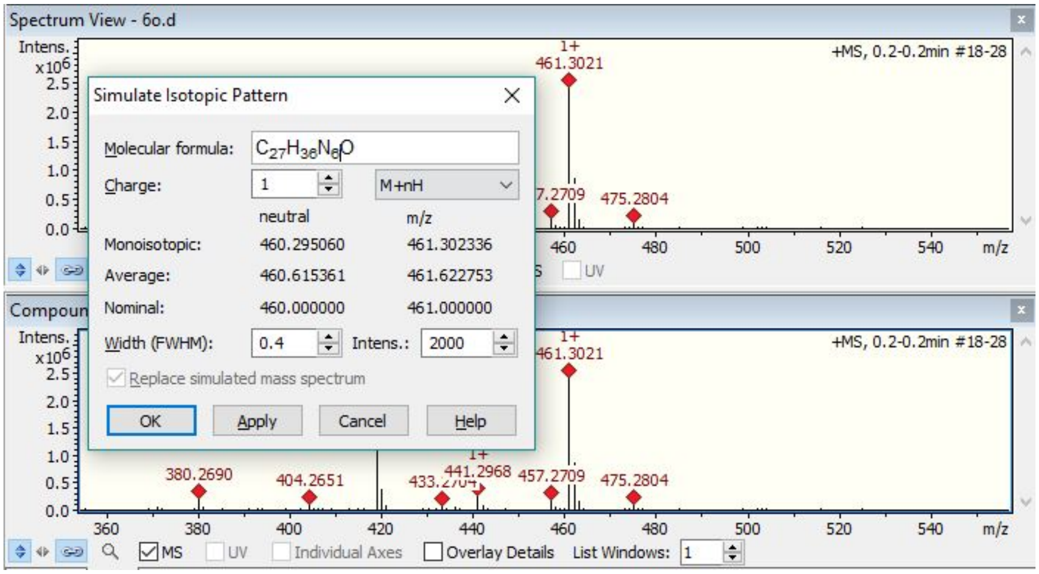

6-((1*H*-1,2,3-Triazol-4-yl)methyl)-2-benzyl-3-(diethylamino)-7-hexyl-6,7-dihydro-5*H*-pyrrolo[3,4-*b*]pyridin-5-one (9l): According to GP-2, pyrrolo[3,4-*b*]pyridin-5-one **6l** (200.0 mg, 0.478 mmol), $TMSN_3$ (127.0 µL, 0.957 mmol), and CuI (3.0 mg, 0.016 mmol), were reacted together in a mixture 0.5 M of DMF/MeOH (9/1 v/v) to afford the 6-((1*H*-1,2,3-triazol-4-yl)methyl)-pyrrolo[3,4-*b*]pyridin-5-one **9l**.Yield 75% (165.0 mg); yellow gum; R_f = 0.35 (hexanes/EtOAc, 1:1); **FT-IR (ATR)** υ_{max}/cm^{-1} 1664 (C=O); ^{1}**H NMR** (500 MHz, $CDCl_3$, 25 °C): δ = 0.64-0.70 (m, 1H), 0.75 (t, *J* = 7.1 Hz, 6H), 0.85 (t, *J* = 7.1 Hz, 6H), 0.95–1.00 (m, 1H), 1.04–1.13 (m, 6H), 1.89–1.95 (m, 1H), 2.09–2.16 (m, 1H), 2.88–2.93 (m, 4H), 4.20 (d, *J* = 14.2 Hz, 1H), 4.35–4.41 (m, 1H), 4.45–4.48 (m, 1H), 5.24 (d, *J* = 15.5, 1H), 7.04–7.17 (m, 5H), 7.62 (s, 1H), 7.79 (s, 1H); ^{13}**C NMR** (126 MHz, $CDCl_3$, 25 °C): δ = 12.1. 14.0, 22.4 (2), 29.0, 29.1, 31.5, 34.8, 39.7, 47.9, 60.5, 124.0, 125.9, 126.0, 128.1, 129.0, 139.6, 146.3, 159.7, 163.2, 167.5; **HRMS (ESI$^+$):** *m/z* calcd. for $C_{27}H_{37}N_6O^+$ [M + H]$^+$ 461.3023, found 461.3021.

^{1}H NMR **9l**

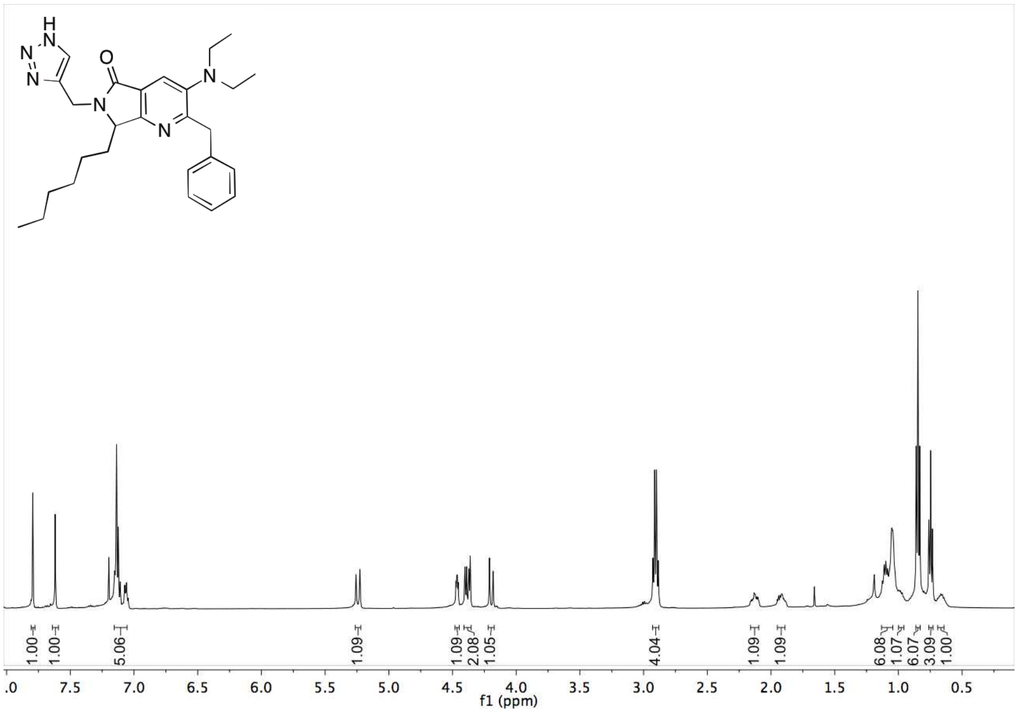

^{13}C NMR **9l**

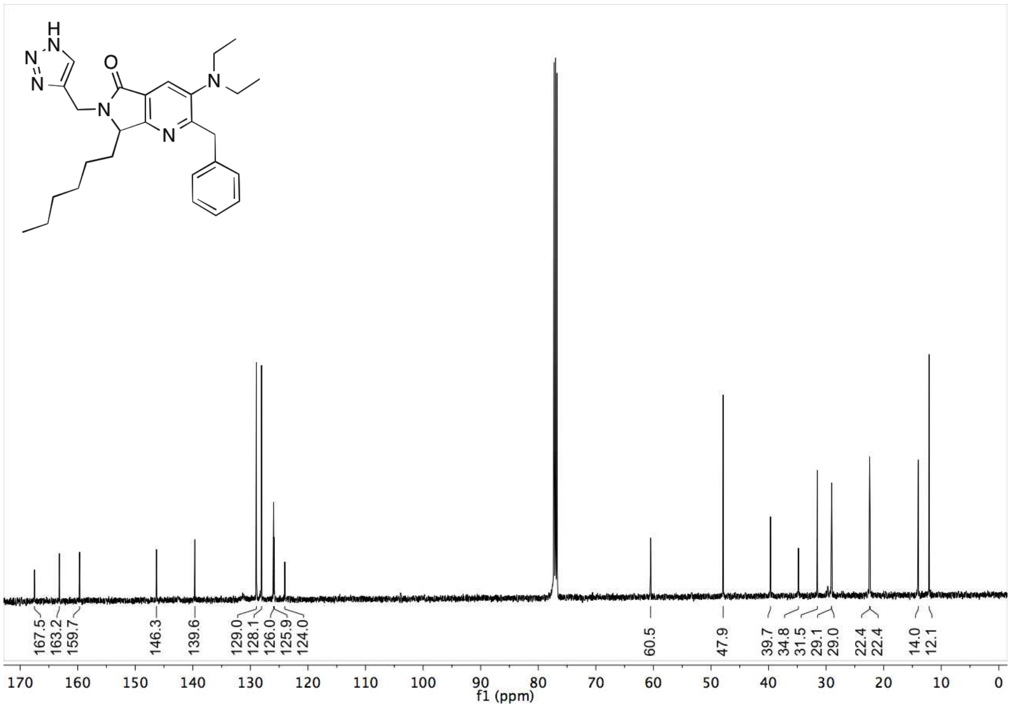

HMRS **9l**

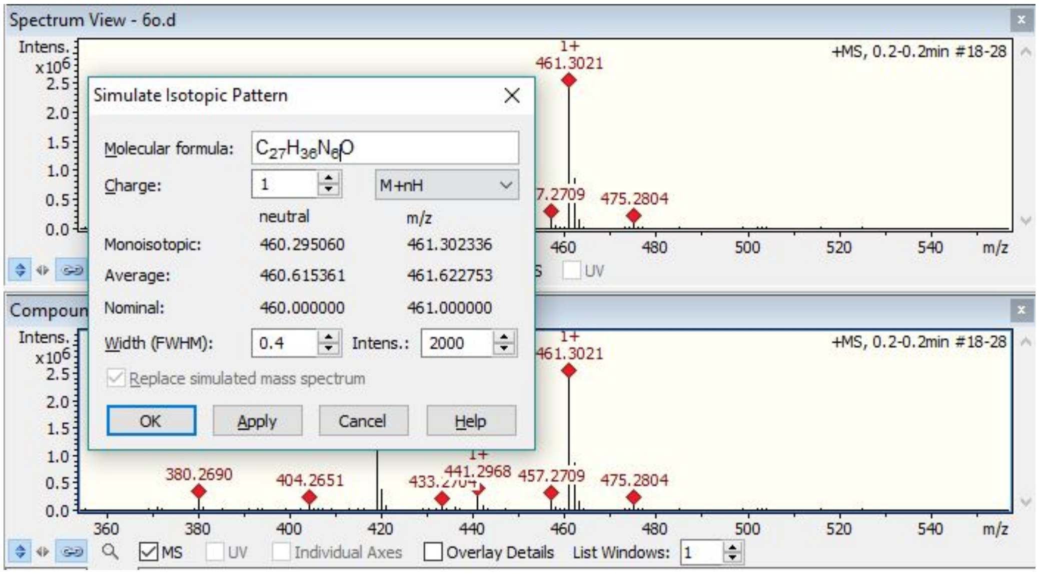

General procedure for the synthesis and characterization of 6-((1-substituted-1*H*-1,2,3-triazol-4-yl)methyl)-pyrrolo[3,4-b]pyridin-5-ones 19a-d (GP-3): To a 0.3 M solution of the pyrrolo[3,4-*b*]pyridin-5-one **6m** (1.0 equiv.) and the corresponding azide (**20a-d**) (1.0 equiv.) in *tert*-BuOH/H_2O (1:1 v/v) in a vial (10 mL) were added and sodium ascorbate (0.20 equiv.) and $CuSO_4$*5H_2O (0.05 equiv.) sequentially. Then, the vial was closed, and the reaction mixture was sonicated at room temperature and monitored by TLC. Once the starting material disappeared, the reaction mixture was diluted in water (5.0 mL) and extracted with dichloromethane (2 x 10 mL). The organic layer was washed with water (2 x 10 mL) and brine (2 x 10 mL). The new organic layer was dried over anhydrous Na_2SO_4 and concentrated under vacuum to afford the crude product. The residue was purified by flash chromatography to afford the corresponding 6-((1-substituted-1*H*-1,2,3-triazol-4-yl)methyl)-pyrrolo[3,4-*b*]pyridin-5-ones **19a-d.**

6-((1-(2-benzoyl-4-chlorophenyl)-1*H*-1,2,3-triazol-4-yl)methyl)-2-benzyl-7-(4-chlorophenyl)-3-morpholino-6,7-dihydro-5*H*-pyrrolo[3,4-*b*]pyridin-5-one (19a): According to GP-3, pyrrolo[3,4-*b*]pyridin-5-one **6m** (50.0 mg, 0.109 mmol), (2-azido-5-chlorophenyl)(phenyl)methanone (28.0 mg, 0.109 mmol), sodium ascorbate (6.5 mg, 0.033 mmol) and $CuSO_4$*5H_2O (3.0 mg, 0.011 mmol), were reacted together in a mixture 0.3 M of tert-BuOH/H_2O (1/1 v/v) to afford the 6-((1*H*-1,2,3-triazol-4-yl)methyl)-pyrrolo[3,4-*b*]pyridin-5-one **19a**. Yield 83% (65.0 mg); white solid; $\boldsymbol{R_f}$ = 0.25 (hexanes/EtOAc, 1:2); **m.p.** 102-104 °C; **FT-IR (ATR)** υ_{max}/cm^{-1} 1693 (C=O), 1671 (C=O); ^{1}**H NMR** (500 MHz, $CDCl_3$, 25 °C): δ = 2.82–2.87 (m, 2H), 2.88–2.94 (m, 2H), 3.83–3.86 (m, 4H), 3.94 (d, *J* = 15.4 Hz, 1H), 4.18 (d, *J* = 13.8 Hz, 1H), 4.39 (d, *J* = 13.8 Hz, 1H), 5.11 (s, 1H), 5.15 (d, *J* = 15.3 Hz, 1H), 7.06–7.12 (m, 5H), 7.14–7.17 (m, 1H), 7.19–7.24 (m, 4H), 7.33–7.36 (m, 2H), 7.56–7.51 (m, 3H), 7.65 (dd, *J* = 2.4, 0.5 Hz, 1H), 7.65 (dd, *J* = 8.5, 2.4 Hz, 1H), 7.73 (s, 1H), 7.89 (s, 1H); ^{13}**C NMR** (126 MHz, $CDCl_3$, 25 °C): δ = 34.7, 40.0, 53.1, 64.0, 67.1, 123.6, 124.0, 125.8, 126.3, 128.2, 128.3, 128.8, 129.1, 129.2, 129.6, 130.1, 131.6, 133.3,

133.5, 133.6, 134.7, 135.6, 135.9, 135.9, 139.2, 144.0, 147.9, 160.0, 162.5, 166.6, 192.8,;
HRMS (ESI⁺): *m/z* calcd. for $C_{40}H_{33}Cl_2N_6O_3^+$ $[M + H]^+$ 715.1986, found 715.1986.

^{1}H NMR **19a**

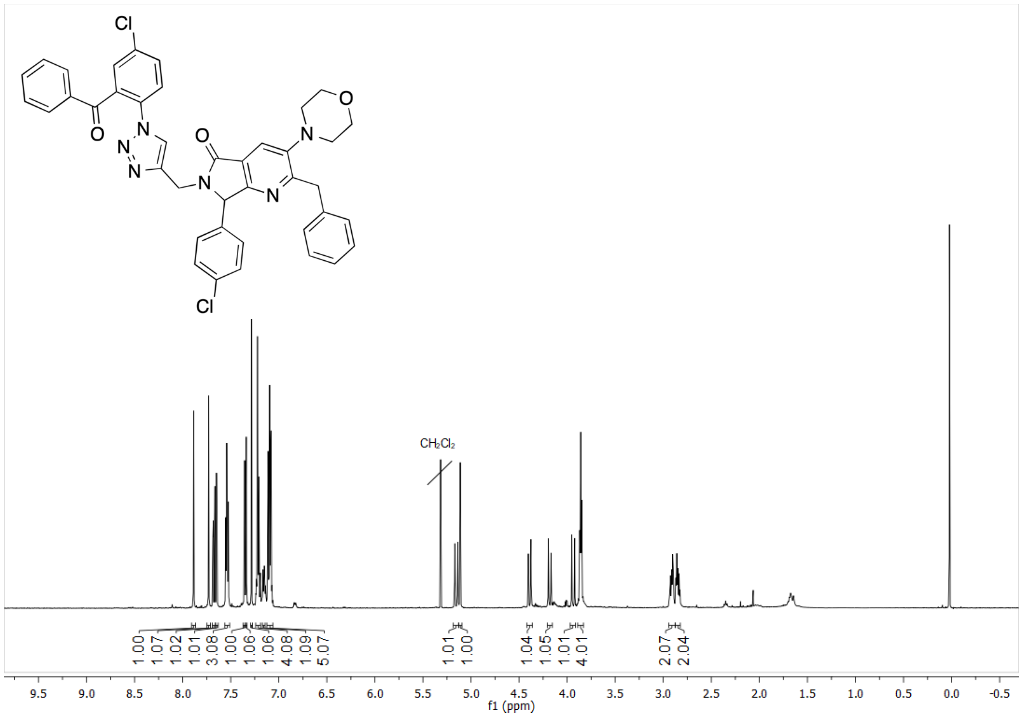

^{13}C NMR **19a**

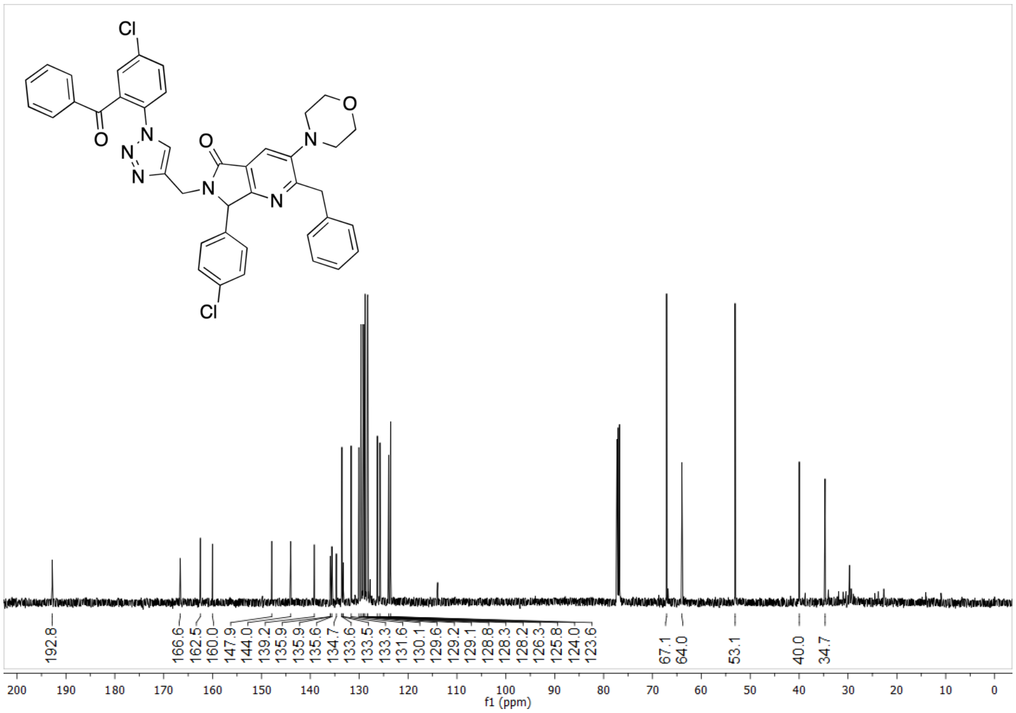

HMRS **19a**

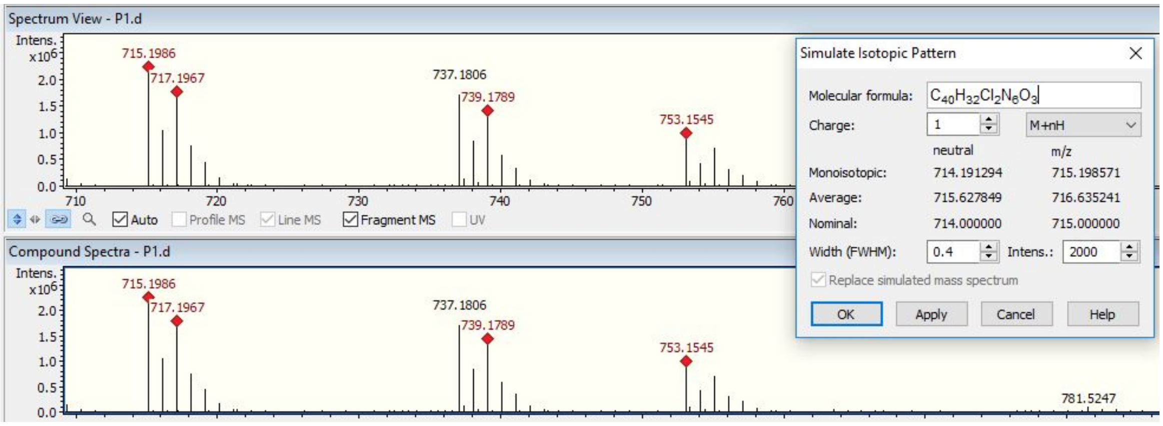

2-benzyl-7-(4-chlorophenyl)-6-((1-(4-chlorophenyl)-1*H*-1,2,3-triazol-4-yl)methyl)-3-morpholino-6,7-dihydro-5*H*-pyrrolo[3,4-*b*]pyridin-5-one (19b): According to GP-3, pyrrolo[3,4-*b*]pyridin-5-one **6m** (60.0 mg, 0.131 mmol), 1-azido-4-chlorobenzene (20.0 mg, 0.131 mmol), sodium ascorbate (7.8 mg, 0.039 mmol) and $CuSO_4*5H_2O$ (3.0 mg, 0.013 mmol), were reacted together in a mixture 0.3 M of tert-BuOH/H_2O (1/1 v/v) to afford the 6-((1*H*-1,2,3-triazol-4-yl)methyl)-pyrrolo[3,4-*b*]pyridin-5-one **19b**. Yield 89% (72.0 mg); white solid; $\boldsymbol{R_f}$ = 0.37 (hexanes/EtOAc, 1:2); **m.p.** 211-213 °C; **FT-IR (ATR)** υ_{max}/cm^{-1} 1698 (C=O); **^{1}H NMR** (500 MHz, $CDCl_3$, 25 °C): δ = 2.77–2.88 (m, 4H), 3.79–3.86 (m, 4H), 4.20 (d, *J* = 13.8 Hz, 1H), 4.24 (d, *J* = 15.4 Hz, 1H), 4.31 (d, *J* = 13.8 Hz, 1H), 5.30 (d, *J* = 15.4 Hz, 1H), 5.67 (s, 1H), 7.13–7.23 (m, 5H), 7.23–7.27 (m, 2H), 7.37–7.42 (m, 2H), 7.47–7.53 (m, 2H), 7.64–7.69 (m, 2H), 7.87 (s, 1H), 7.99 (s, 1H); **^{13}C NMR** (126 MHz, $CDCl_3$, 25 °C):

δ = 35.3, 40.1, 53.0, 64.6, 67.1, 120.9, 121.6, 123.6, 123.7, 126.2, 128.2, 128.8, 129.2, 129.7, 130.0, 133.8, 134.7, 134.7, 135.4, 139.1, 144.3, 147.9, 160.1, 162.5, 167.1; **HRMS (ESI⁺):** *m/z* calcd. for $C_{33}H_{29}Cl_2N_6O_3^+$ $[M + H]^+$ 611.1723, found 611.1722.

^{1}H NMR **19b**

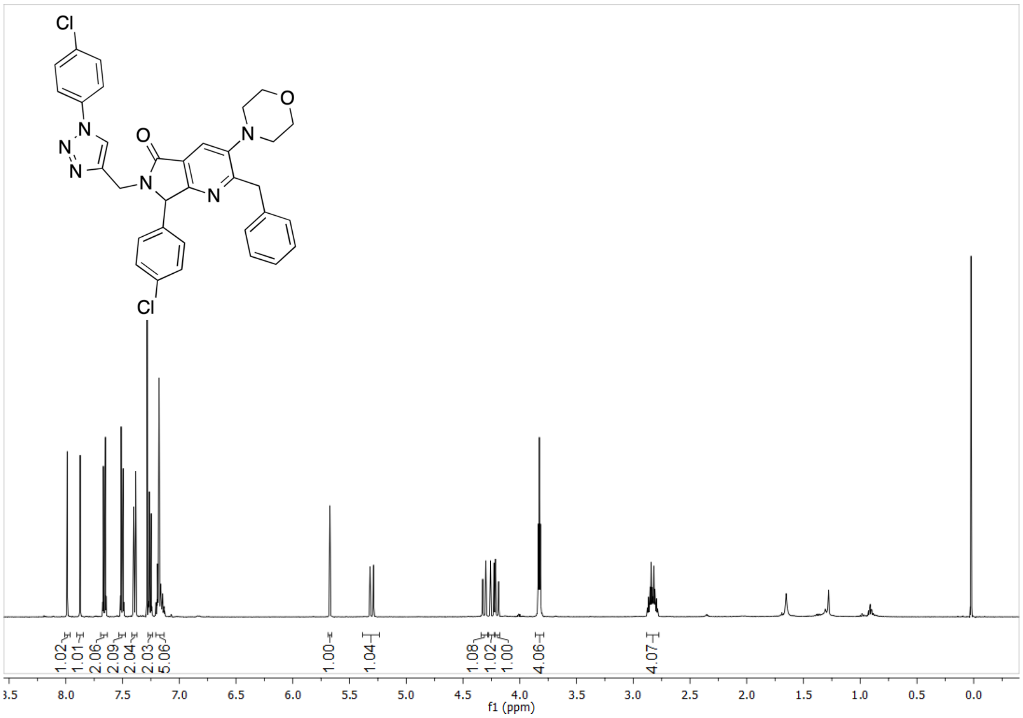

^{13}C NMR **19b**

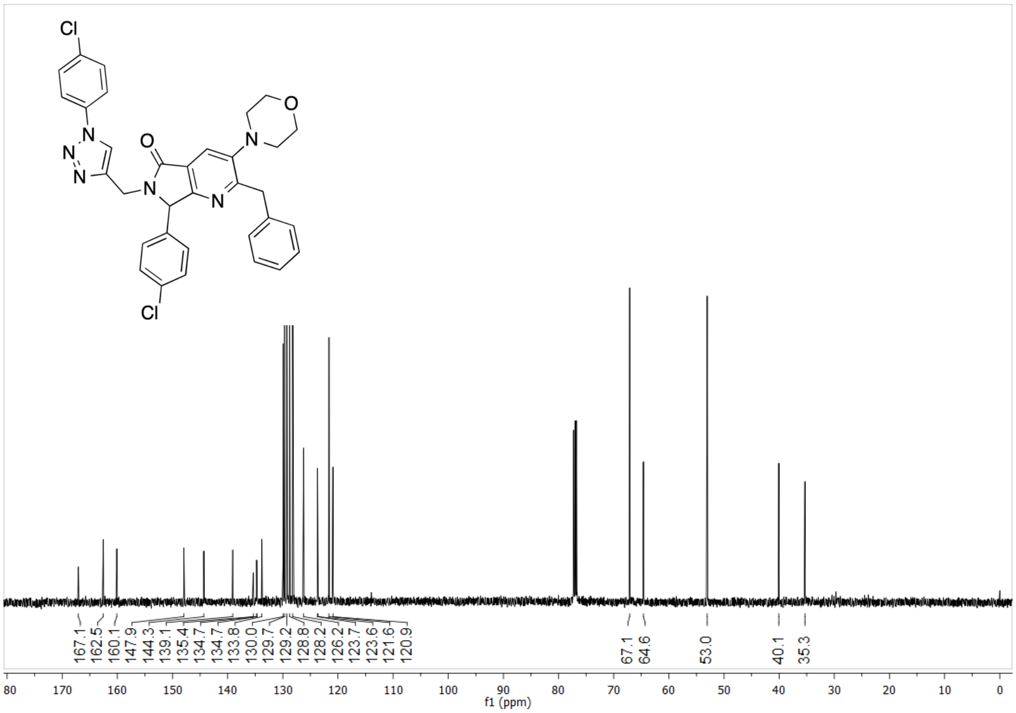

HMRS **19b**

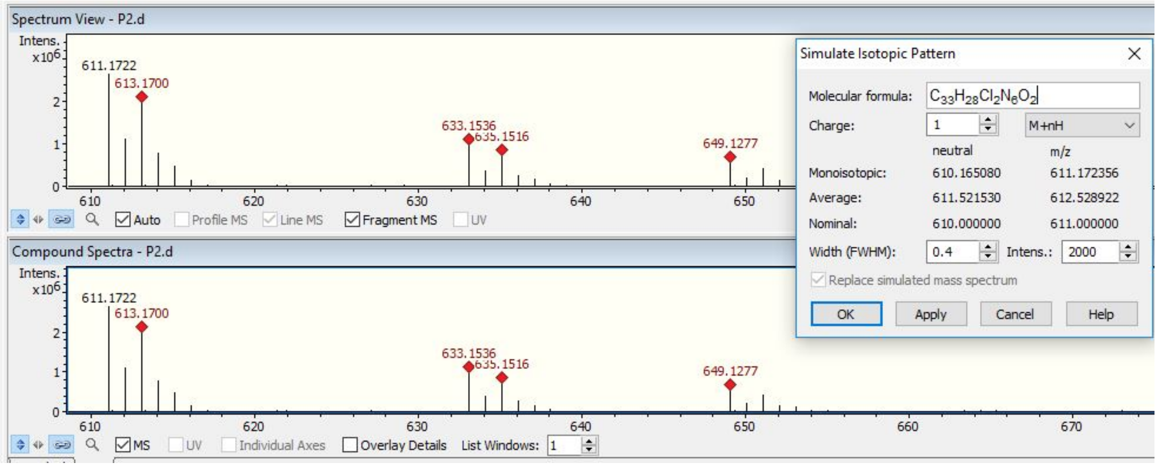

2-benzyl-7-(4-chlorophenyl)-6-((1-(3,4-dimethoxyphenyl)-1*H*-1,2,3-triazol-4-yl)methyl)-3-morpholino-6,7-dihydro-5*H*-pyrrolo[3,4-*b*]pyridin-5-one (19c): According to GP-3, pyrrolo[3,4-*b*]pyridin-5-one **6m** (60.0 mg, 0.131 mmol), 4-azido-1,2-dimethoxybenzene (24.0 mg, 0.131 mmol), sodium ascorbate (7.8 mg, 0.039 mmol) and $CuSO_4$*5H_2O (3.0 mg, 0.013 mmol), were reacted together in a mixture 0.3 M of tert-BuOH/H_2O (1/1 v/v) to afford the 6-((1*H*-1,2,3-triazol-4-yl)methyl)-pyrrolo[3,4-*b*]pyridin-

5-one **19c**. Yield 93% (78.0 mg); white solid; R_f = 0.12 (hexanes/EtOAc, 1:2); **m.p.** 171-173 °C; **FT-IR (ATR)** υ_{max}/cm^{-1} 1696 (C=O); **^{1}H NMR** (500 MHz, $CDCl_3$, 25 °C): δ = 2.78–2.87 (m, 4H), 3.81–3.84 (m, 4H), 3.95 (s, 1H), 3.96 (s, 1H), 4.18–4.25 (m, 2H), 4.31 (d, J = 13.8 Hz, 1H), 5.32 (d, J = 15.2 Hz, 1H), 5.68 (s, 1H), 6.95 (d, J = 8.7 Hz, 1H), 7.13–7.19 (m, 6H), 7.25–7.28 (m, 2H), 7.31 (d, J = 2.5 Hz, 1H), 7.38–7.42 (m, 2H), 7.88 (s, 1H), 7.94 (s, 1H); **^{13}C NMR** (126 MHz, $CDCl_3$, 25 °C): δ = 35.3, 40.1, 53.0, 56.2, 56.2, 64.5, 67.1, 104.9, 111.2, 112.4, 121.2, 123.7, 123.7, 126.2, 128.2, 128.8, 129.2, 129.7, 130.5, 133.8, 134.7, 139.1, 143.9, 147.9, 149.5, 149.8, 160.2, 162.5, 167.0; **HRMS (ESI$^+$):** m/z calcd. for $C_{35}H_{34}ClN_6O_4^+$ $[M + H]^+$ 637.2324, found 637.2328.

^{1}H NMR **19c**

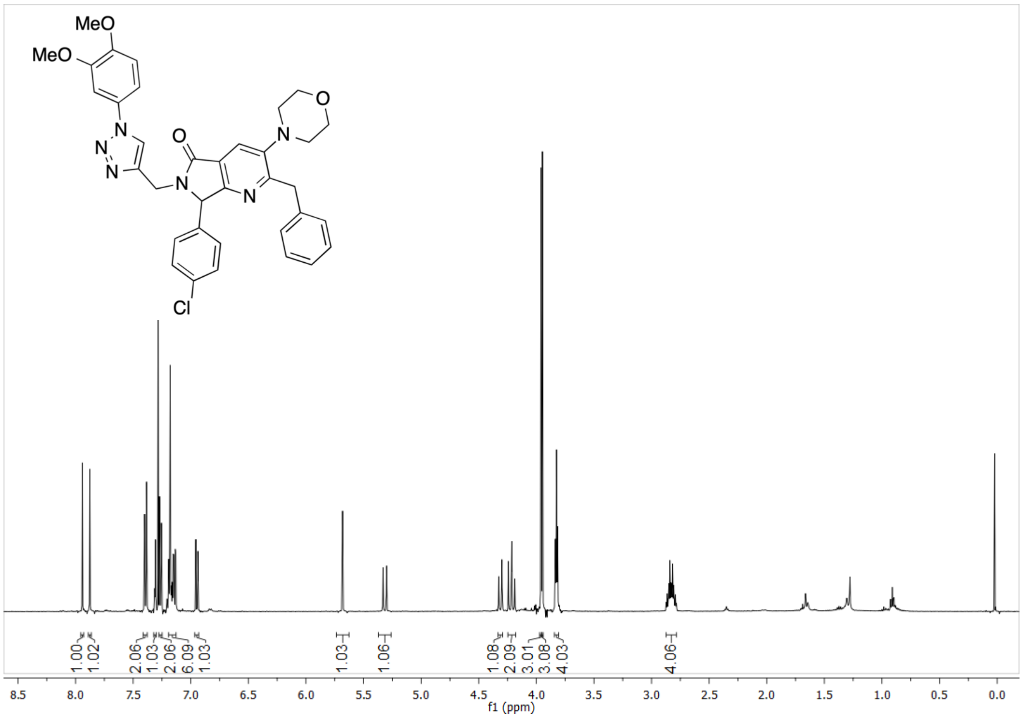

^{13}C NMR **19c**

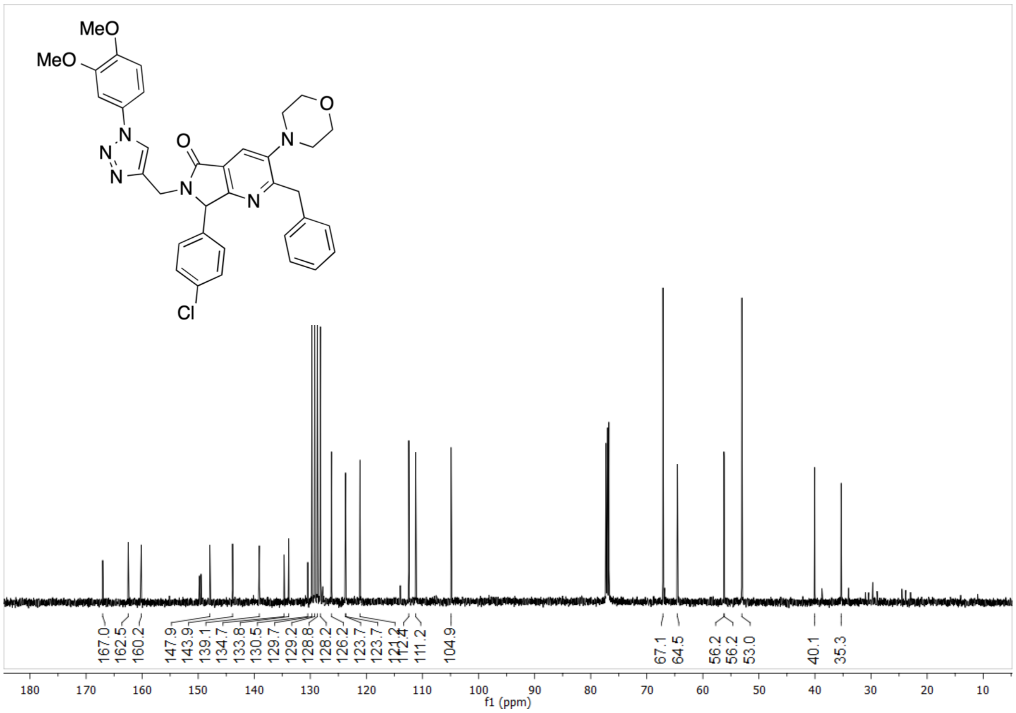

HMRS **19c**

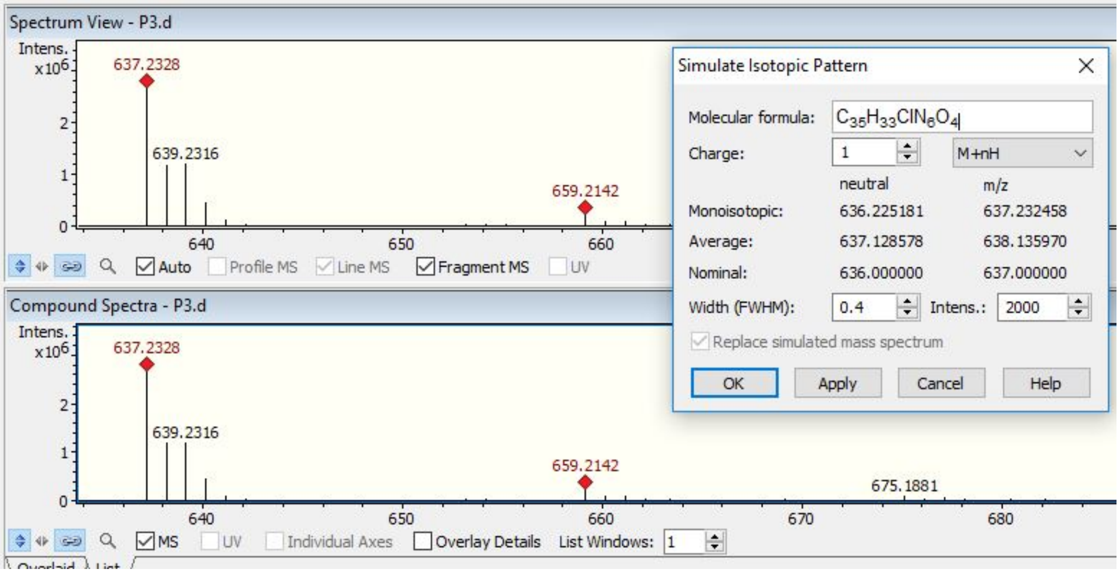

2-benzyl-7-(4-chlorophenyl)-3-morpholino-6-((1-(2-nitrophenyl)-1*H*-1,2,3-triazol-4-yl)methyl)-6,7-dihydro-5*H*-pyrrolo[3,4-*b*]pyridin-5-one (19d): According to GP-3, pyrrolo[3,4-*b*]pyridin-5-one **6m** (60.0 mg, 0.131 mmol), 1-azido-2-nitrobenzene (22.0 mg,

0.131 mmol), sodium ascorbate (7.8 mg, 0.039 mmol) and $CuSO_4$*5H_2O (3.0 mg, 0.013 mmol), were reacted together in a mixture 0.3 M of tert-BuOH/H_2O (1/1 v/v) to afford the 6-((1*H*-1,2,3-triazol-4-yl)methyl)-pyrrolo[3,4-*b*]pyridin-5-one **19d**. Yield 90% (74.0 mg); yellow solid; $\boldsymbol{R_f}$ = 0.10 (hexanes/EtOAc, 1:2); **m.p.** 93-95 °C; **FT-IR (ATR)** υ_{max}/cm^{-1} 1691 (C=O), 1525 (NO_2), 1366 (NO_2); **^{1}H NMR** (500 MHz, $CDCl_3$, 25 °C): δ = 2.77–2.89 (m, 4H), 3.80–3.86 (m, 4H), 4.22 (d, *J* = 13.8 Hz, 1H), 4.26 (d, *J* = 15.4 Hz, 1H), 4.31 (d, *J* = 13.8 Hz, 1H), 5.35 (d, *J* = 15.4 Hz, 1H), 5.68 (s, 1H), 7.13–7.21 (m, 5H), 7.22–7.26 (m, 2H), 7.37–7.41 (m, 2H), 7.56–7.61 (m, 1H), 7.70–7.76 (m, 1H), 7.78–7.82 (m, 1H), 7.87 (s, 1H), 7.89 (s, 1H), 8.08–8.14 (m, 1H); **^{13}C NMR** (126 MHz, $CDCl_3$, 25 °C): δ = 35.2, 40.1, 53.0, 64.6, 130.9, 67.1, 123.6, 123.8, 124.4, 125.6, 126.2, 127.9, 128.2, 128.8, 129.3, 129.7, 133.7, 133.8, 134.7, 139.1, 143.9, 147.9, 160.2, 162.5, 167.0; **HRMS (ESI$^+$):** *m/z* calcd. for $C_{33}H_{29}ClN_7O_4^+$ $[M + H]^+$ 622.1964, found 622. 1972.

^{1}H NMR **19d**

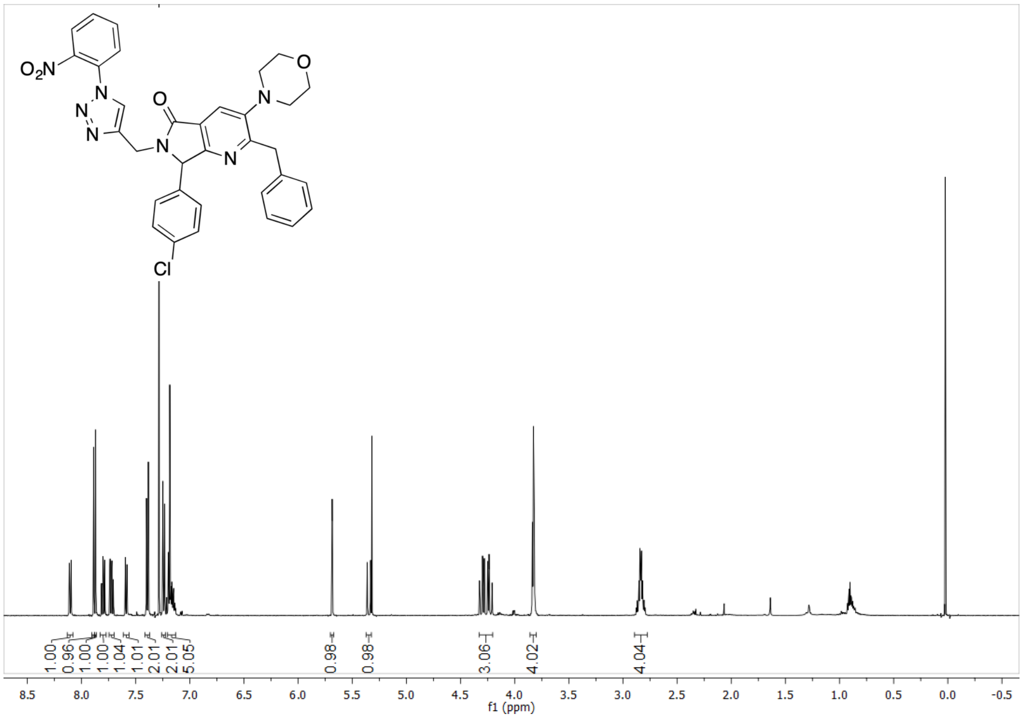

^{13}C NMR **19d**

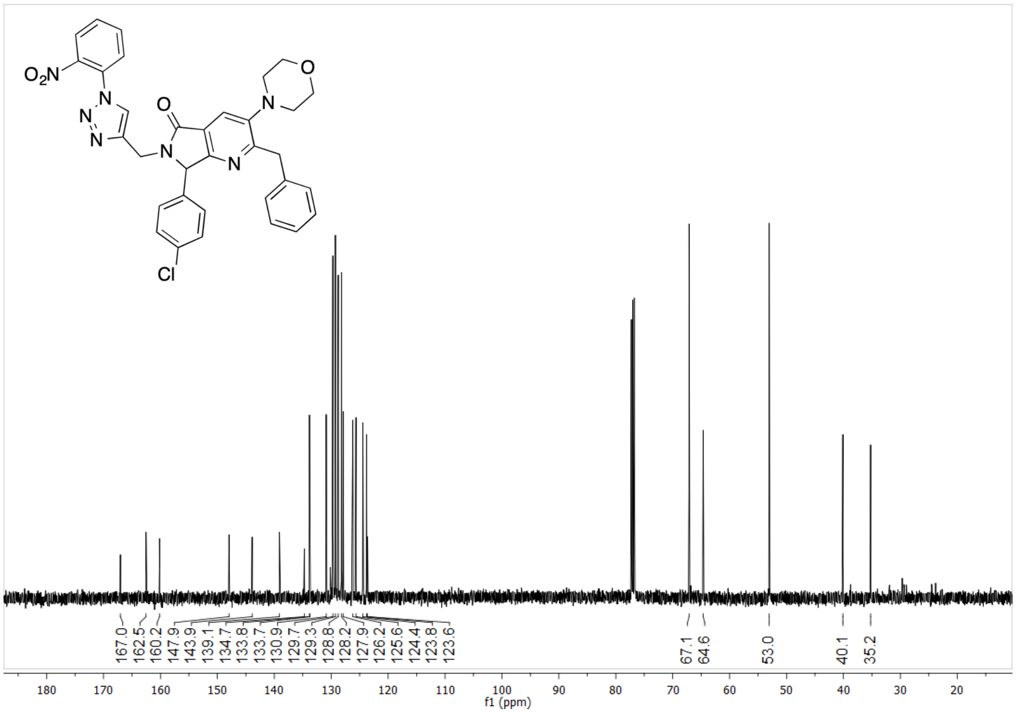
O2N
N
N
N
O
N
N
N
O
Cl
167.0
162.5
160.2
147.9
143.9
139.1
134.7
133.8
133.7
130.9
129.7
129.3
128.8
128.2
127.9
126.2
125.6
124.4
123.8
123.6
67.1
64.6
53.0
40.1
35.2
f1 (ppm)

HMRS **19d**

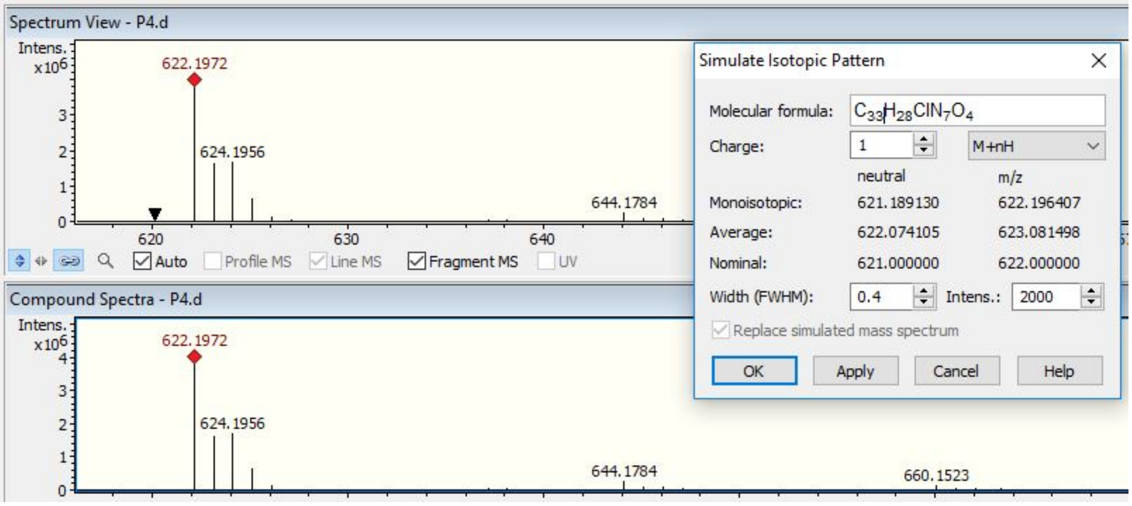
Spectrum View - P4.d
Intens.
x10^6
622.1972
624.1956
644.1784
Auto
Profile MS
Line MS
Fragment MS
UV
Compound Spectra - P4.d
622.1972
624.1956
644.1784
660.1523
Simulate Isotopic Pattern
Molecular formula: C33H28ClN7O4
Charge: 1
M+nH
neutral
m/z
Monoisotopic: 621.189130 622.196407
Average: 622.074105 623.081498
Nominal: 621.000000 622.000000
Width (FWHM): 0.4
Intens.: 2000
Replace simulated mass spectrum
OK
Apply
Cancel
Help
